# Supplementary material for: SAFE, a new therapeutic intervention for families of children with autism: a randomised controlled feasibility trial
Source: BMJ Open. 2020 Dec 31;10(12):e038411. doi: 10.1136/bmjopen-2020-038411 (PMC7780715; doi:10.1136/bmjopen-2020-038411)
Supplement: Supplementary data [file bmjopen-2020-038411supp001.pdf]

Appendix 1
Brief Summary of INTERVENTION MANUAL for SAFE (Systemic Autism-related Family Enabling): an early intervention for families of children with autism and Asperger Syndrome

Professor Rudi Dallos and Doctor Rebecca McKenzie

The intervention manual has been developed from the following bases of evidence and clinical experience:

1. Systemic Family Therapy.

Systemic Family Therapy focuses on promoting positive changes in the relationships within families rather than the isolated behavior of individuals in the family (Dallos & Draper, 2015). Difficulties are seen to be maintained or exacerbated by family dynamics including the organization of families, communicational patterns and repetitive interaction sequences of problems. The combination of these is encapsulated in the idea of the formation of ineffective attempted solutions to their problems which rather than offering solutions can aggravate their difficulties. Systemic Family Therapy embodies a range of approaches and techniques for helping families to explore and re-organise their understanding, relationship patterns, emotional connections and problem-solving abilities. It typically involves a therapist and a supervision team working with one family at a time with close monitoring, based on feedback during and at the end of each session exploring the relevance, helpfulness and applicability of the therapy. Where supervision teams are not available a more flexible model - 'in room consultation' can be employed whereby two therapists support each other in a structure where one therapist takes on the role of monitoring the family's reactions as therapy proceeds and periodically offering feedback as a form of live supervision. A development of this orientation is Multi-Family Therapy whereby groups of families work together to assist and facilitate change, effectively acting as consultants for each other. The SAFE program employs both these versions of Systemic Family Therapy with three single family sessions to explore in detail each family's needs; and two group sessions using concepts and techniques from Multi-Family Therapy

2. Multi-Family Therapy

Multi-Family Therapy is a recognised treatment approach which aims to provide a more empowering, flexible and intensive form of family intervention than single family therapy (Asen & Scholz, 2009). In Multi-Family Therapy similar techniques are employed to those used in single family therapy, but families are encouraged to work together, to be proactive in solving their own problems Multi-Family Therapy aims to help families rediscover their own resources by emphasising how families can take an active role in tackling dilemmas and assisting each other. At the same time families are encouraged to use the group setting to explore how problems have affected family life in consultation with other families and to share their solutions and competencies. This involves a power shift from the therapist-client relationship and encourages an empowering peer-support environment. The sharing of experiences and the dynamics of the group are important components of the treatment.

3. Family Models

The SAFE program incorporates a range of concepts and techniques by integrating systemic and attachment based family models, in particular The Circle of Security Intervention (Powell et al., 2014) and Attachment Narrative Therapy developed by co-applicant Professor Rudi Dallos (Dallos, 2006). In the SAFE intervention, these systemic and attachment models have been combined with an emphasis on visual materials and active, play-based approaches designed to be appropriate for families of children with autism.

McKenzie R, *et al. BMJ Open* 2020; 10:e038411. doi: 10.1136/bmjopen-2020-038411

4. Autism friendly
SAFE has been developed in collaboration with children with autism and their families to be autism friendly. Given the evidence in the literature for a visual processing style, communication difficulties and restricted interests among people with autism, the advice of families is in line with current research. SAFE incorporates a multi-sensory approach using visual materials which are integrated into therapeutic techniques and play-based activities. SAFE also acknowledges and works with the children's talents and areas of special interest. It also recognizes that parents are often extremely well-informed and prefer an approach which recognizes their competencies and helps them to feel empowered rather than de-skilled by therapy.

SAFE sessions and examples of activities:

SAFE involves 5 x 3-hour sessions outlined in a detailed manual. All sessions are facilitated by two therapists trained in the SAFE intervention model. Around 6 families receive the SAFE intervention as a cohort, supported by two therapists. Sessions 1 and 5 are based on Multi-Family Therapy and all parents from the 6 families attend. Sessions 2, 3 and 4 are for single families and all family members attend including siblings and/or grandparents if the family wish. So the 6 families meet each other at the beginning and the end but the in-between sessions are just for their own family.

SAFE is supported by a detailed manual setting out a structure for each session and also a toolkit of activities which can be used flexibly according to family need and the age and symptoms of the child.

Examples of activities include the following:

Tracking a circularity

Families explore the events of positive and problematic cycles of events, such as 'meltdowns' by breaking these down to explore in detail what happened, including actions, understandings, expectations and how people felt. E.g. what happened first, then what happened next? What did you do? What did Mum do? This can allow families to unpick problematic and successful cycles and initiate positive change.

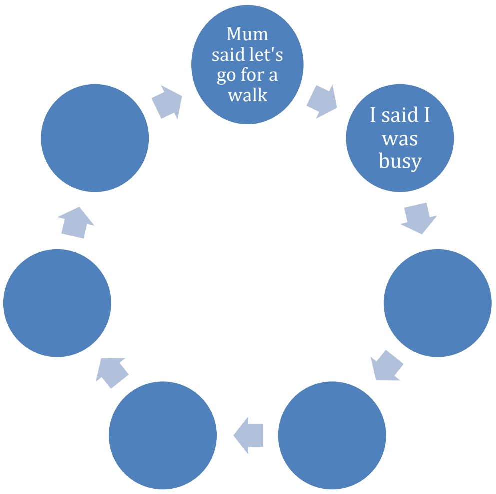

McKenzie R, et al. BMJ Open 2020; 10:e038411. doi: 10.1136/bmjopen-2020-038411

A family genogram

The families are asked to create a genogram (family tree) this is used to explore relationships, perceptions of autism, family narratives and avenues of support and conflict. E.g. who is most supportive to you? Who also has autistic traits? Who is most similar to you? What are the stories you have heard from grandparents etc.

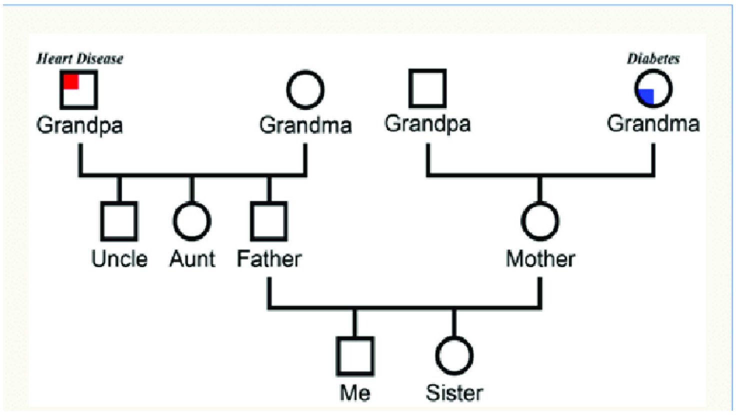

The Circle of Security (COS)

The COS intervention and the graphic designed around it are designed to help parents increase awareness of their children's needs and whether their own responses meet those needs. With increased awareness parents can expand their moment-to-moment parenting choices where needed. The model, therefore, encourages the potential to break the stranglehold of problematic patterns. Discussion can include: the parents as a secure base and haven, procedural memory, parents' attachment needs, implicit responding, children as miscuing.

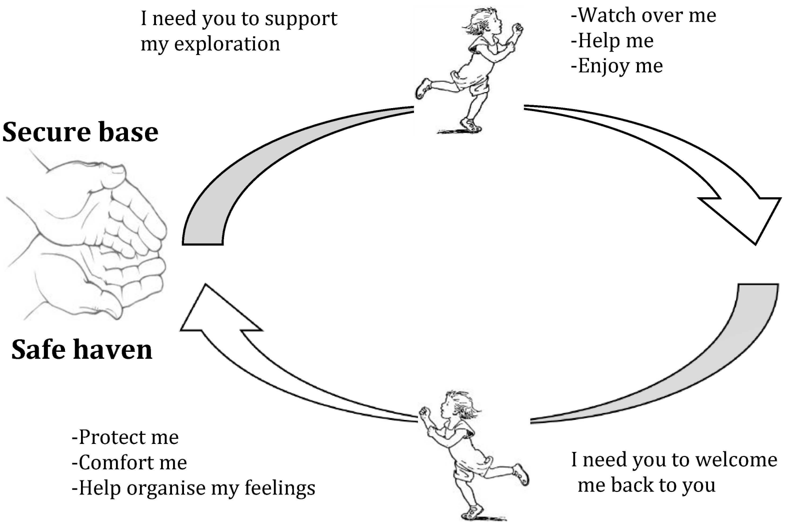

McKenzie R, *et al. BMJ Open* 2020; 10:e038411. doi: 10.1136/bmjopen-2020-038411

Externalising and Self-Autism Mapping (SAM)

Children may be invited to make ‘autism’ from PlayDoh. The parents discuss the child’s responses and elaborate with their own views of what they regard as typical as opposed to autistic and how the influence of the autism may alter in different contexts and reduce or expand as the child develops.

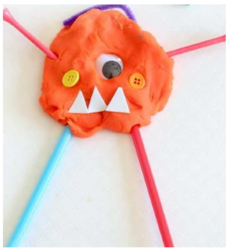

This is developed by inviting the child to engage in mapping the autism (SAM) which is initiated by introducing the child to two figures representing ‘Just me’ and “Me and autism’ Redrawing these figures can involve the child engaged in a discussion of the two figures in terms of what is, and is not affected by autism. At a later date a dotted circle is added and the children can identify aspects of themselves about which they are unsure.

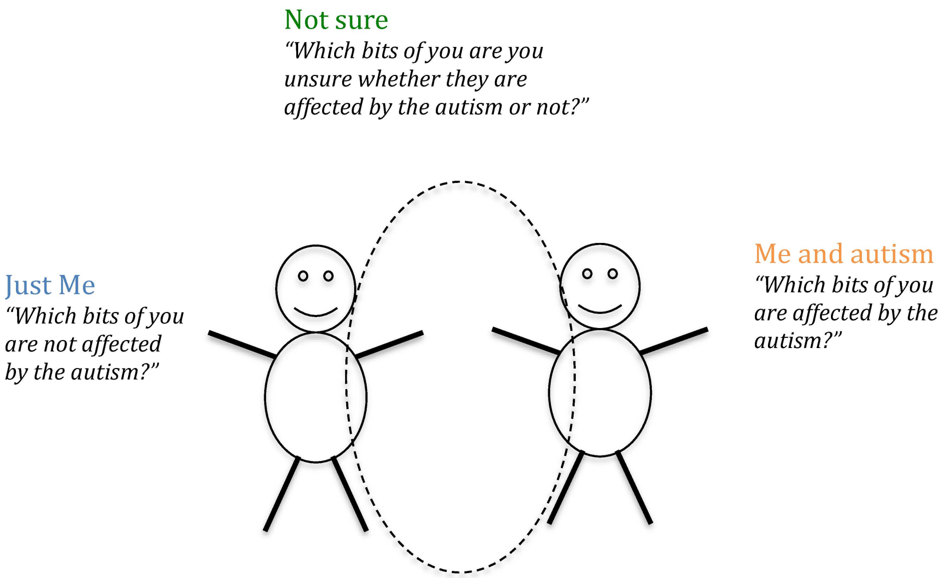

Area of Special Interest (ASI)

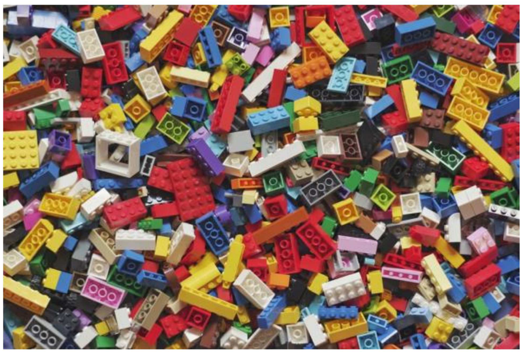

The Child with autism and the siblings are invited to bring an object or present their interest of special interest. This can take and form that the child is comfortable with. The ASI is used to raise the child’s confidence, model communication and explore relationships, perceptions and difficulties. E.g. if mum was a Lego character who would she be? Who would you be? How do the characters manage difficulties? etc.

Button Sculpt

Families use buttons to map and discuss relationships (how close they are to each other) before and after the diagnosis and how they predict it might be in the future. This helps to the families understand the relationships, feelings and perceptions at key times and also reflect on how these may change and develop.

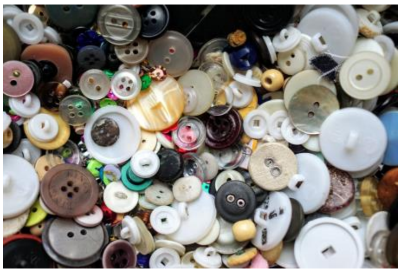

Additional activities

Role play
Often used with tracking to try out new approaches or rehearse successful interactions or strategies.

Reflective conversations
Therapists have a conversation about their thoughts on the family issues and dynamics. The family are invited to listen and then to discuss between themselves and the therapists what was of interest and relevant, what they agreed or disagreed with etc. This is an opportunity to build a sense of openness, support the therapeutic relationships, validate the family, praise successes and also to offer specific suggestions.

Drawing
Family members draw aspects of their life e.g. a typical day or a day at school. Drawing can also be used as part of SAM if the child wishes to draw autism or themselves as part of the activity.

Video feedback activities
Families watch video training materials of other families and use tracking techniques to analyse what is happening, make connections with their own experience and suggest ways forward. This is used in sessions 1 and 5 with the parents. They are asked to watch videos of a child and mum dealing with a meltdown and in the final session a young person with autism describing how he manages emotional and sensory difficulties. These activities are intended to help reflection in a non-challenging way and empower parents to act as consultants to others in similar positions.

Key references

Asen, E., and Scholz, M. (2009) *Multi-Family Therapy: Concepts and Techniques,* London: Routledge

Dallos, R. (2006) *Attachment Narrative Therapy: Integrating Attachment , Systemic and Narrative Therapies.* Maidenhead: Open Univ. Press/McGraw Hill

Dallos, R. and Draper, R. (2015 4th Edn) *An Introduction to Family Therapy*. Maidenhead: Open Univ. Press/McGraw Hill

Powell, B., Cooper, G., Hoffman, K. and Marvin, B. (2014). *The Circle of Security Intervention: Enhancing Attachment in Early – Child Relationships.* London: Guilford Press
