# Supplementary material for: SAFE, a new therapeutic intervention for families of children with autism: a randomised controlled feasibility trial
Source: BMJ Open. 2020 Dec 31;10(12):e038411. doi: 10.1136/bmjopen-2020-038411 (PMC7780715; doi:10.1136/bmjopen-2020-038411)
Supplement: Author's manuscript [file bmjopen-2020-038411.draft_revisions.pdf]

BMJ Open is committed to open peer review. As part of this commitment we make the peer review history of every article we publish publicly available.

When an article is published we post the peer reviewers' comments and the authors' responses online. We also post the versions of the paper that were used during peer review. These are the versions that the peer review comments apply to.

The versions of the paper that follow are the versions that were submitted during the peer review process. They are not the versions of record or the final published versions. They should not be cited or distributed as the published version of this manuscript.

BMJ Open is an open access journal and the full, final, typeset and author-corrected version of record of the manuscript is available on our site with no access controls, subscription charges or pay-per-view fees (http://bmjopen.bmj.com).

If you have any questions on BMJ Open's open peer review process please email info.bmjopen@bmj.com

BMJ Open

SAFE, a new therapeutic intervention for families of children with autism: a randomised controlled feasibility trial.

Journal:	*BMJ Open*
Manuscript ID	bmjopen-2020-038411
Article Type:	Original research
Date Submitted by the Author:	09-Mar-2020
Complete List of Authors:	McKenzie, Rebecca; University of Plymouth, Institute of Education<br>Dallos, Rudi; Department of Clinical Psychology, University of Plymouth<br>Stedmon, Jacqui; The University of Plymouth, Clinical Psychology<br>Hancocks, Helen<br>Vickery, Patricia; Plymouth University, Peninsula Clinical Trials Unit<br>Barton, Andy; South West Research Design Service,<br>Vassallo, Tara<br>Myhill, Craig<br>Chynoweth, Jade; University of Plymouth<br>Ewings, Paul; Research Design Service, Research Office
Keywords:	Child & adolescent psychiatry < PSYCHIATRY, MENTAL HEALTH, Community child health < PAEDIATRICS

SCHOLARONE™
Manuscripts

I, the Submitting Author has the right to grant and does grant on behalf of all authors of the Work (as defined in the below author licence), an exclusive licence and/or a non-exclusive licence for contributions from authors who are: i) UK Crown employees; ii) where BMJ has agreed a CC-BY licence shall apply, and/or iii) in accordance with the terms applicable for US Federal Government officers or employees acting as part of their official duties; on a worldwide, perpetual, irrevocable, royalty-free basis to BMJ Publishing Group Ltd ("BMJ") its licensees and where the relevant Journal is co-owned by BMJ to the co-owners of the Journal, to publish the Work in this journal and any other BMJ products and to exploit all rights, as set out in our licence.

The Submitting Author accepts and understands that any supply made under these terms is made by BMJ to the Submitting Author unless you are acting as an employee on behalf of your employer or a postgraduate student of an affiliated institution which is paying any applicable article publishing charge ("APC") for Open Access articles. Where the Submitting Author wishes to make the Work available on an Open Access basis (and intends to pay the relevant APC), the terms of reuse of such Open Access shall be governed by a Creative Commons licence – details of these licences and which Creative Commons licence will apply to this Work are set out in our licence referred to above.

Other than as permitted in any relevant BMJ Author's Self Archiving Policies, I confirm this Work has not been accepted for publication elsewhere, is not being considered for publication elsewhere and does not duplicate material already published. I confirm all authors consent to publication of this Work and authorise the granting of this licence.

For peer review only

SAFE, a new therapeutic intervention for families of children with autism: a randomised controlled feasibility trial

Rebecca McKenzie[1], Rudi Dallos[2], Jacqui Stedmon[2], Helen Hancocks[3], Patricia Jane Vickery[3], Andy Barton[4], Tara Vassallo[1], Craig Myhill[1], Jade Chynoweth[5], Paul Ewings[5]

Author affiliations

1. Institute of Education, University of Plymouth, Plymouth, UK
2. Department of Clinical Psychology, University of Plymouth, Plymouth, UK
3. Peninsula Clinical Trials Unit, University of Plymouth, Plymouth, UK
4. South West Research Design Service. Plymouth, UK
5. Research Office, Research Design Service, Taunton, UK

Correspondence to: Dr Rebecca McKenzie rebecca.mckenzie@plymouth.ac.uk

Key words: Family Therapy, autism, intervention, feasibility, mental health

Word count excluding title page, abstract, references, figures and tables: 4642

ABSTRACT

Objectives: To establish the feasibility of a definitive randomised controlled trial of SAFE, an intervention for families of children with autism.

Design: A randomised, controlled, multi-centred feasibility study.

Setting: Participants were identified from three NHS diagnosing centres in Plymouth and Cornwall and a community pathway.

For peer review only - http://bmjopen.bmj.com/site/about/guidelines.xhtml

Participants: 34 families of a child with a diagnosis of autism severity level 1 or 2 between 3-16 years. Four families were lost to follow up.

Interventions: Systemic Autism-related Family Enabling (SAFE) is a 16-week manualised Family Therapy-based intervention designed for families of children with autism. SAFE involves families attending five 3-hour sessions led by systemic practitioners.

Primary and secondary outcome measures: The proposed primary outcome measure was the Systemic CORE 15 (SCORE-15). Proposed secondary outcome measures: Patient Health Questionnaire (PHQ)-Somatic Anxiety Depressive Symptoms, the Coding of Attachment-Related Parenting for use with children with Autism (CARP-A), the Child Behaviour Checklist (CBCL), the Reflective Functioning Questionnaire (RFQ) and the Caregiving Helplessness Questionnaire (CGHQ).

Results: All Primary Caregivers retained in the study completed the SCORE-15 at both time points. 34 of the target of 36 families were recruited and 88% of families were retained. Training for therapists was effective. Feedback revealed willingness to undergo randomisation. There was 100% attendance at appropriate sessions for core family members. The SCORE-15 showed reduction in scores for families receiving SAFE compared to controls suggesting positive change. Qualitative data revealed that families found the study acceptable and families receiving SAFE experienced positive change. Feedback indicated that the SCORE-15 should be retained as a primary measure in a future trial, but secondary measures should be reduced.

Conclusions: This study indicates that a larger trial of SAFE is feasible. Findings suggest that SAFE can address current gaps in recommended care, can be confidently delivered by NHS staff and has potential as a beneficial treatment.

Trial registration number: ISCTRN83964946 IRAS 213527

For peer review only

STRENGTHS AND LIMITATIONS OF THIS STUDY

- The study addresses a gap in the available research data, and produces important feasibility information to inform a fully powered randomised controlled trial.
- The study explores the feasibility of using measures of family function and a range of mental health measures.
- Quantitative feasibility data are complemented by qualitative focus groups and interviews.
- The study explores the feasibility of economic analysis measures in a population, which includes adults and their children with developmental disorders.

For peer review only - http://bmjopen.bmj.com/site/about/guidelines.xhtml

- The participants are recruited from two NHS Trusts in adjacent counties in the South West of England, leading to potential bias. A future randomised controlled trial will extend to centres across the UK including Scotland and Wales.

For peer review only

INTRODUCTION

For peer review only - http://bmjopen.bmj.com/site/about/guidelines.xhtml

More than 1% of the UK population has a diagnosis of autism [1]. Families of children with a diagnosis of autism present complex needs. Children with autism have impairments in social interaction, communication, and behavioural flexibility [2]. Autism is widely accepted to have a genetic component and high autistic traits are disproportionally represented among family members [3]. Mental health problems are experienced by more than 70% of individuals with autism and more than 50% of their parents [4,5]. Parents of children with autism are more likely to be hospitalised for mental disorders than parents of typically developing children [6] and mothers of children with autism are reported to have higher unmet needs, more difficulties coping, and lower satisfaction with service interactions than mothers of children with other disabilities [7].

As families of children with autism often exhibit psychological morbidities alongside autism, costs of services to treat these problems are high [8,9]. Furthermore, untreated or unresponsive mental health problems impose societal costs making it hard for parents to interact effectively with services [10], potentially worsening outcomes for children and exacerbating the substantial economic burden of autism [9]. Explanations for high levels of affective disorders in these families include: stress associated with the condition of autism, genetic factors, and intergenerational family dynamics. Parenting children with autism involves stresses associated with challenging behaviour, communicative difficulties, isolation and atypical attachment behaviour displayed by children [11]. Parents of children with autism report that a consequent lack of psychological wellbeing exacerbates maladaptive behaviour in their children [12], which is likely to result in unhelpful cycles of distress and hopelessness.

Previous research demonstrates that experience of trauma and abuse among women is associated with elevated risk of autism developing in their subsequent offspring [13,14]. Hence mothers of children with autism are more likely than the general population to be coping with previous traumatic events. Families of children with autism can experience positive family life, cope well with difficulties and enjoy good relationships with their children [15], but they represent a high-risk group, for whom treatment is disjointed, costly and inadequate [16,17].

A more joined-up approach is required, focusing on communication within families, strategies for coping with challenging behaviour and associated problems and building on strengths and relationships to alleviate mental health difficulties. The current study should be placed in the context of international calls for improved services and care for families of children with autism at country level [18], alongside National Institute of Health and Care excellence (NICE) guidelines and recommendations [19,20], as well as developments regarding children's service provision proposed by the Munroe Report [21,22]. Families of children with autism themselves highlight the importance of professionals working therapeutically with children and the wider family [6].

SAFE (Systemic Autism-related Family Enabling) is a systemic family therapy approach designed to address autism related needs including problem solving, mental health difficulties and problematic behaviour [23]. Systemic Family Therapy is a well-recognised, evidence-based psychotherapeutic approach [24].

For peer review only - http://bmjopen.bmj.com/site/about/guidelines.xhtml

Despite evidence that family therapies can provide benefits to children with autism and their parents [25,26] its efficacy for treating this condition has not been subject to a randomised controlled trial. In addition, existing research overwhelmingly shows that families of children with autism want interventions which make real improvements to their daily life and sense of wellbeing [27,28].

Aims and Objectives

The aim of the study was to establish the feasibility of a definitive randomised controlled trial of SAFE.

Our objectives were to:

1. Demonstrate ability to identify, recruit and randomise eligible families
2. Verify that proposed outcome measures and follow-up are acceptable, and targets for loss to follow-up are achievable
3. Assess adherence of families to the intervention
4. Gather quantitative data on outcomes to inform the design (and sample size) of the future trial
5. Adapt existing resource use questionnaire and assess the feasibility of preference based instruments for this population to facilitate a future economic evaluation
6. As part of the feasibility of process evaluation, collect data on the families' experience of SAFE and the study itself
7. As part of the feasibility of process evaluation, ensure that proposed training arrangements are effective and scalable
8. Provide operational experience to manage the future trial

METHODS AND ANALYSIS

The study comprised a randomised, controlled, multi-centred feasibility study including families of children with a diagnosis of autism (CWA) [23]

Participants

Participants were identified from three NHS diagnosing centres in Plymouth and Cornwall via a search of clinical records or recruitment directly after diagnosis. A community recruitment pathway was added after commencement of the study to alleviate the burden on clinical staff and increase participation. Families were recruited by posters in local community venues.

Eligibility was determined from clinical records or diagnostic letter as well as discussion with the family. A member of the research team gained consent during the first home visit.

Eligibility criteria:

For peer review only - http://bmjopen.bmj.com/site/about/guidelines.xhtml

• Family includes CWA, aged 3-16 years
• Diagnosis of Autism Spectrum Disorder (ASD) severity level 1 or 2
• Diagnosed within 12 months of consenting to the study
• If other diagnoses are present, ASD must be primary diagnosis
• Family are willing to comply with study requirements

Exclusion criteria:
• Child with ASD severity level 3
• Child with ASD and intellectual impairment
• Serious concomitant illness in child or family, or other circumstances such that they are unable to comply with study requirements
• Families who may be a risk to safety of research staff
• Insufficient English language, or capacity for parent/child to consent/assent to the study

The intervention

SAFE is a manualised intensive programme of systemic family therapy designed to treat maladaptive autistic symptoms and mental health-related difficulties encountered by families of children with autism. SAFE lead therapists are qualified NHS systemic practitioners who received an additional 4-day training course in SAFE principles and delivery. SAFE provides an array of therapeutic activities based on attachment theory, established systemic practice and the known visual processing preferences of people with autism [29,30]. SAFE is best seen as a toolkit with a variety of activities which can be applied to family therapy flexibly. Activities include visual tasks, drawing, modelling, role-play and tracking circular patterns. Sessions are led by family need and the therapists and family work collaboratively, often in a playful way, using family resources, therapist expertise and the tools that SAFE provides. SAFE draws heavily from well-documented active and playful approaches in family therapy practice and literature [31]. Each therapy session will include two therapists with a minimum of intermediate family therapy level of qualification and 4 days training in SAFE principles. Between weeks 1 and 16, families allocated to the SAFE intervention attended five 3-hour SAFE therapy sessions. Sessions 1 and 5 are multi-family sessions and will take place in a community setting. Sessions 2, 3 and 4 were for individual families and took place in a community venue or the family home. Following completion of the therapy programme, families attended a group follow-up session at 24 weeks post-allocation. Trained support workers from local voluntary groups attended this follow-up session and gave the families information about continued support through existing networks.

Support as Usually Employed

Families were typically offered a post-diagnosis follow-up appointment with the diagnosing paediatrician. Parents of children whose symptoms were not severe tended to be directed to local authority parenting classes. In some cases families were also directed to relevant resources, for example, The National Autism Society, Gateway ASD and the NHS Child and Adolescent Mental Health Services. For families where a member was experiencing depression or anxiety, treatment varied and was not linked to autism-related

For peer review only - http://bmjopen.bmj.com/site/about/guidelines.xhtml

care. Referral was often through the GP with patients receiving cognitive behavioural therapy as part of the improved access for psychological therapies service and or medication.

Procedure

Families were recruited in four cohorts of between 6 and 12 families (two cohorts from Plymouth and two from Cornwall). Post-baseline, once sufficient families had been recruited to establish a cohort families were randomised 2:1 to the SAFE intervention plus Support as Usually Employed (SAFE+SUE) or Support as Usually Employed only (SUE). Twenty two families were allocated to SAFE+SUE and 12 families were allocated to SUE (See Figure 1). Clinical staff and research staff collecting outcome data were blinded to allocation. Those in the intervention arm received a preparatory home visit from a therapist.

Outcome measure data were collected at baseline and follow-up, 24 weeks post-randomisation. Additional data were collected from the intervention participants: post allocation, at each therapy session and at the feedback day (See Figure 1).

Feasibility outcome measures

- Ability to identify, recruit and randomise eligible families
- Acceptability of proposed outcome measures and follow-up schedule to participants, and whether targets for loss to follow-up are achievable
- Adherence of therapists and families to the intervention
- Ability to gather quantitative data on outcomes
- Appropriateness of resource use questionnaires and preference-based instruments for this population
- Effectiveness and scalability of training arrangements

Clinical outcome measures

The proposed primary outcome measure was, the Systemic CORE 15 (SCORE-15) [30]. This is a 15-item paper-based survey, which is the primary measure of family functioning employed in Children and Young People's Improving Access to Psychological Therapies national programme, and is the gold standard for assessing the impact of family therapy on quality of life in the UK [33]. Every able family member were asked to complete the SCORE-15 at baseline and 32 weeks.

The SCORE-15 is particularly well-suited as a primary measure for this study as it is a measure for the whole family and, like SAFE, it has a systemic framework based on the premise that relational dynamics are central to the wellbeing of all family members. In addition, the three dimensions of SAFE are well matched to the needs of this population in that The SCORE-15 is sensitive to change in aspects of mental health and problem solving defined by the three sub-categories measuring: family strengths, resilience and coping (*Strengths and Adaptability*); communication and understanding within the family (*Disrupted Communication*) and changes in ability to manage problems (*Overwhelmed by Difficulties*). The SCORE

For peer review only - http://bmjopen.bmj.com/site/about/guidelines.xhtml

also correlates with measures of parental satisfaction, mental health, strengths and difficulties and life satisfaction [34].

Scores on the proposed secondary outcome measures, which index changes in child behaviour, child-parent attachment, anxiety and depression:

- Patient Health Questionnaire (PHQ)-Somatic Anxiety Depressive Symptoms completed by parents. This comprises the PHQ-9 measuring depression and the generalised anxiety disorder, GAD-7 measuring anxiety [35].
- Adapted mutuality subscale of the Coding of Attachment-Related Parenting for use with children with Autism (CARP-A) [36] completed by the whole family. The CARP-A is a validated observational measure of a child with autism's attachment behaviour towards their carer.
- The Child Behaviour Checklist [37] completed by the Primary Caregiver. This is a 30-item paper-based survey, which detects emotional and behavioural problems.
- The Reflective Functioning Questionnaire [37] completed by parents, measures ability to understand own and others' mental states.
- Caregiving Helplessness Questionnaire (CGHQ) [38] completed by the parents. This is a 26-item questionnaire designed to assess aspects of disorganised caregiving.

Scores on proposed generic health economic outcome measures also provided data on the feasibility of cost-effectiveness analysis:

- EuroQol 5 dimensions, a standardised generic instrument for measuring health outcome.
- Child Health Utility 9 Dimensions, a paediatric generic preference-based measure of health-related quality of life.
- Resource Use Questionnaire (RUQ), a paper-based questionnaire completed by parent about his/her child's use of healthcare and social resources. The RUQ is designed to identify the NHS and social care resource use for the economic evaluation.

Proposed qualitative outcomes assessed via focus groups:

- Acceptability of SAFE and the trial process for participants and therapists
- Reasons for declining and withdrawing from the study

Additional process measures completed after each SAFE session by therapists and families allocated to SAFE:

- The Client Satisfaction Questionnaire (CSQ-8 completed by families)
- Helpful Aspects of Therapy Questionnaire - assessing what was helpful/unhelpful in each session (HAT completed by families)
- The Training Checklist and Questionnaire monitoring protocol adherence (TCQ completed by therapists)

For peer review only - http://bmjopen.bmj.com/site/about/guidelines.xhtml

Quantitative analysis

Prior to analysis a detailed statistical analysis plan was created and agreed by the research team. For each outcome measure, the relevant scores were calculated and presented descriptively by trial arm.
Where available, published guidelines were used to process, score and summarise the measures including, for example, the use of imputation in the event of missing items on a questionnaire. Summary measures were calculated as appropriate, for example, means and SD, medians and ranges, numbers and percentages in categories. The only analysis contrasting the two groups was an interval estimate in the form of a 95% confidence interval (CI) for the primary outcome, the SCORE-15.

Qualitative analysis

Prior to analysis a qualitative analysis plan was agreed. Focus group interviews were audio recorded and transcribed verbatim. Consequent qualitative data was managed using proprietary computer-assisted qualitative data analysis software, NVivo 10, and analysed thematically [39,40]. Summaries of process measures were compiled including quantitative data from Likert scales and thematic analysis of open-ended questions.

Place Figure 1 here *(Participant Flow Diagram -CONSORT)*

RESULTS

Recruitment and randomisation

Of 138 families screened, 106 were eligible and 32 ineligible. Forty-five eligible families expressed interest of which 34 were recruited (Figure 1). Since randomisation only occurred once a complete cohort (between 6 and 12 families at each site) was recruited, the time between consent (when baseline questionnaires were completed) and randomisation varied. Data on relevant dates were available for 33 families, for whom there was a median (range) of 21 days (2 to 111) between baseline questionnaire completion and randomisation, with a mean (SD) of 34 (32) days. Nineteen families completed the 24-week assessments for which dates were available. Six of the 19 dates were less than 22 weeks from randomisation due to time constraints, none were more than 26 weeks. Baseline demographics were similar for CWA in both groups (See Table 1)

Table 1. Summary statistics of baseline and demographic characteristics for CWA

	SAFE + SUE (n=22)	SUE (n=12)	Total (n=34)
Age			

For peer review only - http://bmjopen.bmj.com/site/about/guidelines.xhtml

Mean (SD)	9.5 (2.7)	9.4 (2.4)	9.5 (2.5)
Median (range)	*10 (5-14)*	*9 (6-13)*	*10 (5-14)*
Gender n (%)			
Male	17 (77)	9 (75)	26 (76)
Female	5 (22)	3 (25)	8 (24)
Ethnicity n (%)			
White	22 (100)	12 (100)	34 (100)

Acceptability of outcome measures and follow-up schedule and loss to follow-up

One SUE family was lost to follow-up and three SAFE+SUE families were lost to follow up (Figure 1). Mechanisms were in place to report and record serious adverse events (SAEs) related to mental health from consent until participants completed the follow-up or withdrew from the study. No SAEs were reported. For the purposes of the study the core family was taken to be the Primary Caregiver (all mothers) and CWA. In one case parents were joint Primary Caregivers. Families chose who else would attend sessions and complete data. At baseline, 34 families and 122 family members provided data. At the 24-week assessment, 30 families and 97 family members provided data. Different outcomes are completed by different combinations of family members. The Primary Caregiver provided data on all measures at baseline, and 22 of the 23 other caregivers (fathers) did likewise. There was a small number of caregivers who did not provide data at 24 weeks for some questionnaires. The completion of the SCORE by the CWA and siblings was optional. Reasons for non-completion by CWA were: child was too young (n=4), the visit could not be arranged (n=1), the child did not want to (n=1) and reason unknown (n=1) (see Figure 1).

Adherence of families to the intervention

Full engagement with the SAFE intervention involves five 3-hour therapy sessions. The first and last of which are multi-family sessions for the parents (children can attend if they wish) between weeks 1 and 16, with a final feedback day at 22 weeks. The Primary Caregiver was present for these sessions, except for one mother who was absent for session 1, but the father attended. The CWA was always present at the individual sessions 2-4. Five children were also present at parent sessions 1 and 5.

Quantitative data on outcomes

Results for the SCORE-15, including expressions of uncertainty (95% CI) for estimates by randomised groups are shown in Table 2.

Table 2. Values for SCORE-15, based on the “total” computed for each dimension and overall for each person. (reduction in values indicates positive change)

	Baseline	24 weeks post randomisation	Difference

For peer review only - http://bmjopen.bmj.com/site/about/guidelines.xhtml

	SAFE+SUE		SUE		SAFE+SUE		SUE				
	Mean	SD	Mean	SD	Mean	SD	Mean	SD	95% CI		N
	Strengths and adaptability										
CWA	11.4	3.3	12.8	3.9	12.1	4.0	12.6	4.8	-3.81	3.29	21
Primary CG	10.0	2.7	9.4	2.3	10.9	3.1	10.6	2.3	-1.96	1.92	30
Other*	11.0	3.6	10.2	3.0	11.1	3.9	11.2	4.6	-1.59	3.87	43
Whole family*	10.8	3.3	10.5	3.2	11.2	3.7	11.3	3.9	-0.89	2.07	94
	Overwhelmed by difficulties										
CWA	12.3	3.9	12.3	5.5	13.5	4.2	14.1	5.5	-2.89	5.58	21
Primary CG	12.4	3.5	12.1	4.0	11.5	2.7	12.7	4.1	-0.69	3.79	30
Other*	12.1	3.9	12.2	3.9	11.7	3.2	13.1	4.2	-0.63	3.86	43
Whole family*	12.2	3.7	12.2	4.2	12.1	3.4	13.2	4.4	-0.03	2.96	94
	Disrupted communication										
CWA	12.8	4.3	11.8	4.0	13.2	4.4	11.4	2.9	-5.02	3.08	21
Primary CG	11.9	4.8	9.6	2.8	10.2	2.6	9.6	2.5	-1.95	2.04	30
Other*	12.9	4.6	12.6	4.2	12.3	3.4	11.2	3.6	-2.91	1.59	43
Whole family*	12.6	4.5	11.5	3.9	11.9	3.6	10.7	3.1	-2.03	0.92	94
	Overall Score										
CWA	36.4	10.1	36.8	11.9	38.8	11.3	38.1	12.0	-9.68	11.22	21
Primary CG	34.3	9.7	31.1	7.6	32.5	7.2	33.0	7.9	-3.49	7.35	30
Other*	36.1	10.2	34.9	9.5	35.1	7.5	35.5	10.4	-3.81	7.24	43
Whole family*	35.7	9.7	34.1	9.5	35.2	8.6	35.2	9.8	-2.09	5.26	94

* scores averaged across all relevant family members

^ difference between SUE and SAFE + SUE at 24 weeks, adjusting for baseline in ANCOVA; based on individuals with complete data at both baseline and 24-week assessment; no allowance for hierarchy of data

CG = Caregiver
CWA = Child with a diagnosis of autism

For peer review only

Feasibility of using economic evaluation instruments for this population

Completeness of health economics data was achieved in intervention and control groups for the primary care based, community based and mental based services at 100% response rate at baseline dropping to 82% and 83% at 24 weeks in intervention and control groups, respectively. The outpatient based services had response rates of 68% and 67% at baseline, but completeness improved to 82% and 83% in intervention and control groups respectively at 24 weeks.

For EQ-5D-5L, at baseline the control group had a 100% response rate. At 24 weeks the response rates were 82% and 83% of participants in intervention and control groups, respectively.

As expected CHU-9D responses were largely provided by CWA (completed by the Primary Caregiver where necessary). Response rate in the intervention group fell from 77% to 73% for CWAs but remained constant with three (14%) Primary Caregiver responses at baseline and 24 weeks. In the control group, response rate fell from 83% to 58% in CWAs and 25% to 17% in Primary Caregivers (mothers) from baseline to week 24.

For peer review only - http://bmjopen.bmj.com/site/about/guidelines.xhtml

Despite indications for increased appropriate service use coupled with reduced cost over time for SAFE+SUE CWAs, the economic evaluation focused on CWA, consequently insufficient data was collected for whole family service use and costs.

The families' experience of the study and the SAFE intervention

Data on family experience of SAFE and of the study itself were collected via process evaluation measures, the Helpful Aspects of Therapy Questionnaire (HAT) and the Client Satisfaction Questionnaire (CSQ) completed by family members after each therapy session and via the family feedback day.

The family feedback qualitative data revealed that families did not find the completion of CARP-A Lego task and questionnaires onerous:

> *"didn't mind so much doing like the Lego task I mean like it's something that we like to do anyway … but um the questionnaires didn't have a problem with those either"*

Families felt that there were too many questionnaires, but the approachable nature of the research staff made the task easier:

> *"it was all fine … it was A [Research Assistant] that came in did it and she's so friendly and approachable that it made it easy…"*

Data suggested that families generally took part in the study for altruistic reasons. They wanted to be part of a study which could potentially increase support post-diagnosis. Limited feedback from SUE families suggests that this helped them to accept their role in the study.

> *" I thought actually this could be a way of helping future parents of diagnosed children to get the support and advice that wasn't there when I got the diagnosis"*

SAFE families found the intervention helpful with mean scores for all sessions on the HAT being rated by children and adults as helpful or very helpful. The multi-family sessions made families feel less alone and judged.

> *"Talking to other parents and feeling listened to and supported, "talking about daily issues and sharing these with the other parents, generally feeling that I am not alone"*

SAFE therapists were seen as allies and the activities helped with family problems leading to increased understanding between family members, improved mental health, feeling less isolated, being able to step back and reflect, being more confident in tackling problems.

For peer review only - http://bmjopen.bmj.com/site/about/guidelines.xhtml

"breaking down problem events and looking at causes, outcomes, choices made. compared a recent to past events and saw progress and maturity in decision making and actions"

"*actually being able to hear that I'm doing OK and actually not as bad as what I thought I was, was nice. It was something that helped me with my mental and emotional state"*

The focus group data also revealed that families felt sessions at home were too distracting, the sessions should be shorter but more of them and more activities for younger children were needed.

Effectiveness and scalability of training

The therapists completed the Training Checklist Questionnaire (TCQ) after each session. Focus groups and interviews were also conducted with the therapists after completion of the intervention. The therapists felt that SAFE was an inspirational and effective intervention, was non-pathologising and child centred. The training was motivational and thorough. In more than 70% of sessions therapists reported feeling confident, that the sessions were effective and the activities were easy to deliver. They felt supervision was very helpful. They highlighted that the gap between training and intervention was too long.

Operational experience moving forward.

The Chief Investigator (CI) kept the study on time and within budget despite multiple challenges. The support and advice of the Trial Steering Committee (TSC), the PenCTU and the research team facilitated the development of the skill set of the CI in preparation for a definitive trial.

Patient and Public Involvement

This study grew from the articulated needs of over 90 families surveyed and interviewed through the Plymouth Autism Network. The main aim was to develop a family-orientated support package and conduct a feasibility study, definitive trial and ultimately implement an intervention to be offered to all families after diagnosis. To achieve this aim in a sustainable manner we included families as partners at every stage of design and application.

Prior to commencement of the trial a pilot study was conducted with families acting as consultants. From the pilot we recruited interested families to form the SAFE Family Consultation Group. Their representative was a co-applicant on the initial bid and is employed as a research assistant. The consultation group were consulted at every stage of the trial including the initial application, developing, refining and administering the intervention, developing trial materials, recruitment, training and dissemination.

The family representative attended all trial management and research meetings and took part in all training. She disseminates to consultation group members and where appropriate, meetings with the wider group

For peer review only - http://bmjopen.bmj.com/site/about/guidelines.xhtml

have been held. The input from the families has been invaluable in engaging participants and ensuring their wellbeing.

Patient and Public Involvement (PPI) was an intrinsic part of this study and our continued positive engagement and partnership with families of children with autism is a strength. The research emerged from the difficulties articulated by families and we worked with them to develop solutions. We feel that this way of working made a substantial impact on the outcomes for the families receiving the intervention and the fact that the feedback from families was overwhelmingly positive. We also believe that PPI ensured a more ethical and thorough study which did not lose sight of the ultimate aim. The input of families throughout provided considerable motivation for the research team in delivering the study despite the challenges faced.

DISCUSSION

To our knowledge, this study is unique in evaluating the feasibility and efficacy of a Family Therapy-based intervention designed specifically for families of children with a diagnosis of autism. The results indicate that a larger trial of SAFE would be feasible. The findings also suggest that offering SAFE for families post-diagnosis is both feasible and potentially efficacious.

Progression to a definitive trial was supported based on pre-determined criteria met in the following ways: a) 34 of the target of 36 families were recruited. Family feedback revealed willingness to undergo randomisation as families felt the study had potential to extend services. b) 88% of families were retained. c) There was 100% attendance at appropriate sessions for core family members. d) For the SCORE-15, complete data were available at both time points and for every dimension for 88% Primary Caregivers, 62% CWA and 55% other family members. All Primary Caregivers retained in the study completed the SCORE-15 at both time points. e) Collecting health and social care resource utilisation data and health-related quality of life data for CWA was feasible. f) Process evaluation and feedback day showed that in more than 70% of sessions therapists felt confident, that sessions were effective and easy to deliver. g) The qualitative data from families indicates potential for efficacy as SAFE was rated as helpful or very helpful in addressing problems. Qualitative analysis revealed the following themes of positive change: therapist helping reflection, increased understanding, feeling closer, feeling more confident, more able to reflect and problem solve, improved communication and feeling less isolated. A common thread across all qualitative data was increased mental wellbeing among family members receiving SAFE. Although we acknowledge that the current study was not powered to detect a difference, the SCORE-15 showed marked reduction in scores for SAFE+SUE suggesting positive change. A reduction of this magnitude indicates potential proof of efficacy [32] (See Figure 2).

For peer review only - http://bmjopen.bmj.com/site/about/guidelines.xhtml
For peer review only

Place Figure 2 here *(Mean change in SCORE-15 subcategories for SAFE+SUE (SAFE) and SUE only - Control)*

Additional analysis of the SCORE-15 data revealed that the most marked indications of positive change were experienced by Primary Caregivers with high anxiety as measured by the GAD-7 scale. The relationship between anxiety scores and values on the SCORE-15 is important as SAFE seeks to alleviate mental health issues. An indication that a reduction in anxiety and improved family function can be captured by our proposed measures and that data suggests positive change for those participants with acute difficulties receiving the intervention is therefore promising (See Figure 3).

Place Figure 3 here *(SCORE-15 total mean change for Primary Caregivers with low and high anxiety - a reduction = positive change; high anxiety > 7 on GAD-7)*

The feasibility study alongside previous research [4,5,6,7] strongly suggests that these families are an at-risk group who experience very limited support post diagnosis. One of the primary aims will be to carry out a definitive trial in order to address international [18] and national recommended good practice in relation to children with autism and their families [NICE guidelines 19,20]. These recommend psychosocial interventions, which improve sensitivity, responsiveness and communication within families. They also call for interventions which prevent challenging behaviour by creating care packages which address co-existing mental health problems, and provide support for families. NICE guidelines associated with child mental health also ask for the provision of psychological therapies including family therapy; the need for parents' psychiatric problems to be treated, for children's mental health to improve; and management of developmental conditions to be in parallel with mental health interventions.

NICE research recommendations associated with autism call for randomised controlled trials exploring family interventions that are designed to reduce challenging behaviour, alleviate stress and improve quality of life [19]. Our long-term goals are also informed by the Munroe Report [41], which called for children's services to offer family-centred, therapeutic intervention rather than risk management. Additionally, the current implementation of the Improved Access to Psychological Therapies scheme for children [42] promotes proactive attachment-based and family therapy approaches. SAFE has the potential to address this gap by providing a family-based intervention which is sustainable, extends existing services and is beneficial to families.

For peer review only - http://bmjopen.bmj.com/site/about/guidelines.xhtml

LIMITATIONS

Use of the primary measure the SCORE-15 was limited for children below 7-years and an adapted more visual version will be developed so children below 7-years can contribute. The economic evaluation focused solely on the child with a diagnosis. To ensure sufficient data is collected, a subsequent trial should include longer-term service use and costs for all family members. Families felt there were too many measures and repetition. For any subsequent trial the outcome measures would be reduced and adapted. The Reflective Functioning Questionnaire (RFQ) replicated information from other measures and the Child Behaviour Checklist (CBCL) was lengthy and complex. These measures would not be used in a subsequent trial. We propose the SCORE-15 (measuring perceived change in family functioning) be retained as our primary outcome measure for Primary Caregiver alongside secondary measures being the adapted SCORE-15 (for other family members including the youngest children), PHQ-SADS (anxiety scale), CGHQ (measuring change in caregiver helplessness) and the CARP-A (measuring mutuality between family members).

Recruitment and organisation of SAFE sessions was challenging. Therapists and families preferred shorter sessions in community venues. A recruitment open day and appointments for SAFE sessions with predetermined shorter appointments are proposed. Due to the lack of ethnic diversity in Plymouth and Cornwall our participants were all white. This will need to be addressed in any future trial. Given that prevalence differs between ethnic groups [43,44,45] and beliefs and support networks may also differ, it is essential for appropriate implementation across the UK, and internationally, that any future trial reflects ethnic diversity. Any definitive trial will, therefore, include centres which have an ethnically diverse client base.

CONCLUSIONS

To our knowledge this is the first study of Family Therapy as an intervention for families of children with a diagnosis of autism. The proposed primary outcome measure, the SCORE-15, indicated potential benefits for the intervention group compared to controls in coping with problems, developing strengths and communicating within families. Qualitative data showed that family members felt the intervention improved understanding, communication, confidence, mental health, reflection, problem solving and sense of isolation. Attendance and completion of outcome measures was acceptable and NHS therapists were effectively skilled after receiving brief SAFE training. This study suggests that SAFE can address current gaps in recommended care post-diagnosis and can and should be subject to a definitive trial. The findings indicate that SAFE can be effectively and confidently delivered by existing NHS staff and has potential to be a feasible and beneficial treatment for these families.

For peer review only - http://bmjopen.bmj.com/site/about/guidelines.xhtml

For peer review only

Acknowledgements: The authors would like to thank the SAFE Family Consultation Group for their input and expertise and all of the participating families from whom we learnt so much. The authors would like to thank James Cook, Mary Hosken, Dr Ben Whalley, Dr Antonieta Medina-Lara and Professor Julian Archer for their help in developing and conducting this study. The authors would also like to give special recognition to the dedication, hard work and sensitivity of Helen Hancocks.

Contributors: RM, JV and HH were responsible for the overall development of the study and trial management. RM, HH, RD, CM, PE, AB, TV and the Family Consultation Group were involved in the conception, administration and conduct of the study. PE, JC and AB provided methodological expertise and advice on quantitative analysis, PE and JC provided statistical expertise and analysis. RD was the lead researcher, with the support of JS, on design of the intervention, supervision of therapists and qualitative analysis. TV, with the support of the Family Consultation Group, and CM advised on design, ethic and data collection particularly from the participant perspective. All authors made substantial contributions to drafting, revision and approval of this document.

Funding: This work was supported by the National Institute for Health Research (NIHR), grant number: PB-PG-0815-20058, and by Autistica, grant number: 7239

Competing interests: Contributors are co-applicants or employed research staff on the SAFE project, which receives funds from both NIHR and Autistica. Professor Rudi Dallos holds joint Intellectual Property rights for the SAFE intervention with the University of Plymouth.

REFERENCES

1. National Autistic Society, *Autism Facts and History*, 2018
2. *American Psychiatric Association. Diagnostic and Statistical Manual of Mental Disorders,* 5th edn. Washington, DC: American Psychiatric Association, 2013.
3. Sasson NJ, Lam KSL, Parlier M, et al. Autism and the broad autism phenotype: familial patterns and intergenerational transmission. *J Neurodev Disord* 2013;5:11-18.
4. Dykens EM, Fisher MH, Taylor JL, et al. Reducing Distress in Mothers of Children with Autism and Other Disabilities: A Randomized Trial. *Pediatrics* 2014; 2013-3164.
5. Hallett V, Ronald A, Colvert, E, et al. Exploring anxiety symptoms in a large-scale twin study of children with autism spectrum disorders, their co-twins and controls. *J Child Psychol Psychiatry* 2013;54:1176-1185.
6. Daniels JL, Forssen U, Hultman M, et al. Parental Psychiatric Disorders Associated With Autism Spectrum Disorders in the Offspring. *Pediatrics* 2008;121:1357-1162.

For peer review only - http://bmjopen.bmj.com/site/about/guidelines.xhtml

7. Siklos S, Kerns KA. Assessing Need for Social Support in Parents of Children with Autism and Down Syndrome. *J Autism Dev Disord* 2008;36:921-933.

8. McCrone P, Dhanasiri S, Patel A, et al. *The cost of mental health care in England to 2026*. Executive Summary, London: The King's Fund. 2008

9. Buescher AVS, Cidav Z, Knapp M, et al. Costs of Autism Spectrum Disorders in the United Kingdom and the United States. *JAMA Pediatr* 2014;e1-e8.

10. Osborne LA, McHugh L, Saunders J, et al. Parenting Stress Reduces the Effectiveness of Early Teaching Interventions for Autistic Spectrum Disorders. *J Autism Dev Disord* 2008;38:1092-1103.

11. Pisula E. *Parenting Stress in Mothers and Fathers of Children with Autism Spectrum Disorders,* A Comprehensive Book on Autism Spectrum Disorders, Dr. Mohammad-Reza Mohammadi (Ed.) 2011. Available from: http://www.intechopen.com/books/a-comprehensive- bookon- autism-spectrum-disorders/parenting-stress-in-mothers-and-fathers-of-children-with-autismspectrum-disorders

12. Nikmat AW, Ahmad M, Oon NL, et al. Stress and psychological wellbeing among parents of children with autism spectrum disorder. *ASEAN J of Psychiatr* 2008;9:65-72.

13. Roberts AL, Koenen KC, Lyall K, Ascherio A, Weisskopf MG. Women's posttraumatic stress symptoms and autism spectrum disorder in their children. *Res Autism Spectr Disord* 2014;8(6):608-616.

14. Roberts AL, Lyall K, Rich-Edwards JW, Ascherio A, Weisskopf MG. Maternal exposure to intimate partner abuse before birth is associated with autism spectrum disorder in offspring. *Autism* 2016;20(1):26-36.

15. Markoulakis R, Fletcher P, Bryden P. Seeing the glass half full: benefits to the lived experiences of female primary caregivers of children with autism. *Clin Nurse Spec* 2012;26:48–56.

16. Murphy M, Bolton PF, Pickles A, et al. Personality traits of the relatives of autistic probands. *Psychol Med* 2000;30:1411-1424.

17. Blackledge JT, Hayes SC. Using Acceptance and Commitment Training in the Support of Parents of Children Diagnosed with Autism. *Child Fam Behav Ther* 2006:28:1-18.

18. World Health Organization. WHA67. 8 Comprehensive and coordinated efforts for the management of autism spectrum disorders 2014.

19. NICE guidelines [CG170] https://www.nice.org.uk/guidance/cg170/chapter/patient-centred-care

20. NICE guidelines [CG128] https://www.nice.org.uk/guidance/cg128

21. Monroe E. *The Munroe Review of Child protection: Final report,* Dept. of Education, HM Stationary Office, UK 2011.

22. Goodman S, Trowler I. *Social work re-claimed: Innovative frameworks for child and social work Practice*. London: Jessica Kingsley 2012.

23. McKenzie R, Dallos R, Stedmon J, Hancocks H, Vickery PJ, Ewings P, Barton A, Vassallo T, Myhill C. SAFE, a new therapeutic intervention for families of children with autism: study protocol for a feasibility randomised controlled trial. *BMJ Open* 2019:9:5:e025006.

24. Stratton P. Report On The Evidence Base Of Systemic Family Therapy. *Association for Family Therapy* 2005.

25. Monteiro M. *Family Therapy and the Autism Spectrum: Autism Conversations in Narrative Practice*. London: Routledge 2016.

26. Estrada AU, Pinsof WF. The effectiveness of family therapies for selected behavioural disorders of childhood. *J Marital Fam Ther* 1995:21: 403-424.

27. Pellicano E, Dinsmore A, Charman T. What should autism research focus upon? Community views and priorities from the United Kingdom. *Autism, Special Issue Article* 2014:1-15.

For peer review only - http://bmjopen.bmj.com/site/about/guidelines.xhtml

28. Wallace S, Parr J, Hardy, A. *One in a hundred: Putting families at the heart of autism research*. London: Autistica 2013.
29. Buron KD, Wolfberg P. *Learners on the autism spectrum: Preparing highly qualified educators*. Shawnee: AAPC Publishing, 2008:114–37.
30. Grandin T, Scariano MM. *Emergence: Labeled autistic*. New York, NY: Warner Books, 1986.
31. Dallos R, Draper R. *An introduction to family therapy: Systemic theory and practice*: McGraw-Hill Education, 2015.
32. Stratton P, Bland J, Janes E, *et al*. Developing an indicator of family function and a practicable outcome measure for systemic family and couple therapy: the SCORE. *J Fam Ther* 2010;32:232–58.
33. SHIFT Self-Harm Intervention, Family Therapy: a randomised controlled trial of family therapy vs. treatment as usual for young people seen after second or subsequent episodes of self harm. Protocol Grant Number: 470772 HTA Reference Number: 07/33/01. 2015.
34. Stratton P, Lask J, Bland J, Nowotny E, Evans C, Singh R, Janes E, Peppiatt A. Detecting therapeutic improvement early in therapy: validation of the SCORE-15 index of family functioning and change. *J Fam Ther* 2014; 36:1:3-19.
35. Spitzer RL, Kroenke K, Williams JBW. Validation and utility of a selfreport version of PRIME-MD: the PHQ Primary Care Study. *JAMA* 1999;282:1737–44.
36. Blazey L. *Coding of Attachment-Related Parenting for use with children with Autism (CARP-A)*. Manchester: Manchester University, 2007.
37. Moss E, Bureau JF, Cyr C, *et al*. Correlates of attachment at age 3: construct validity of the preschool attachment classification system. *Dev Psychol* 2004;40:323–34.
38. Solomon J, George C, eds. *Disorganised Attachment and Caregiving*. Guilford: New York, 2011.
39. Braun V, Clarke V. Using thematic analysis in psychology. *Qual Res Psychol* 2006;3:77–101.
40. Green J, Thorogood N. *Qualitative Health Methods for health Research*. London: Sage Publications, 2004.
41. Munroe E. *The Munroe Review of Child protection: Final report,* Dept. of Education, HM Stationary Office, UK 2011.
42. Department of Health. *IAPT Implementation Plan: National Guidelines for Regional Delivery*. 2008. Available at www.iapt.nhs.uk
43. Keen DV, Reid FD, Arnone D. Autism, ethnicity and maternal immigration. *BJPsych* 2010;196:4:274-81.
44. Mandell DS, Wiggins LD, Carpenter LA, Daniels J, DiGuiseppi C, Durkin MS, Giarelli E, Morrier MJ,. Nicholas JS, Pinto-Martin JA, Shattuck PT. Racial/ethnic disparities in the identification of children with autism spectrum disorders *Am J Public Health* 2009;99:3:493-8.
45. Barnevik–Olsson M, Gillberg C, Fernell E. Prevalence of autism in children born to Somali parents living in Sweden: a brief report *Dev Med Child Neurol* 2008;50:8:598-601.

For peer review only - http://bmjopen.bmj.com/site/about/guidelines.xhtml

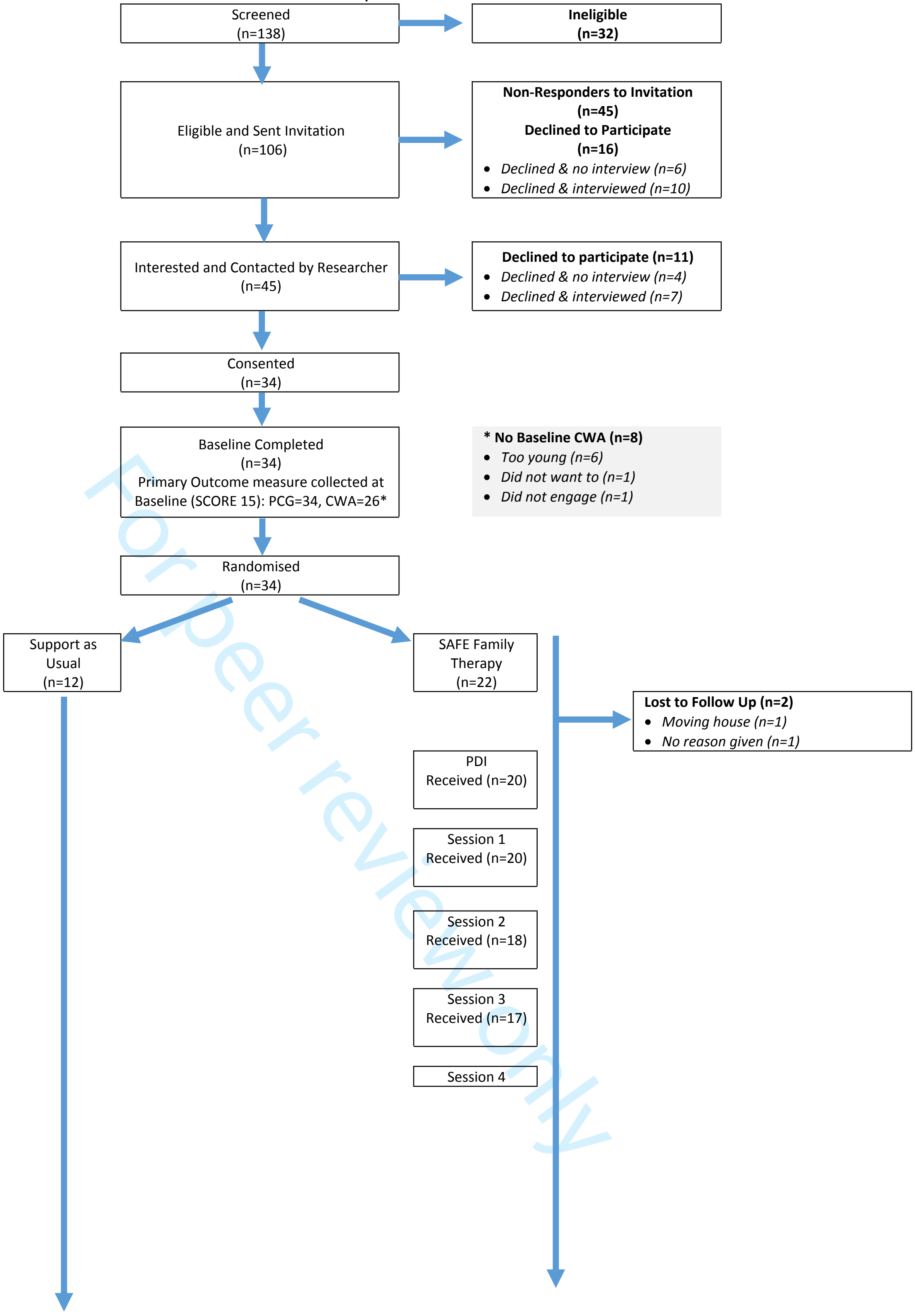

For peer review only

Figures

For paper entitled: SAFE, a new therapeutic intervention for families of children with autism: a randomised controlled feasibility trial. McKenzie et al., 2020

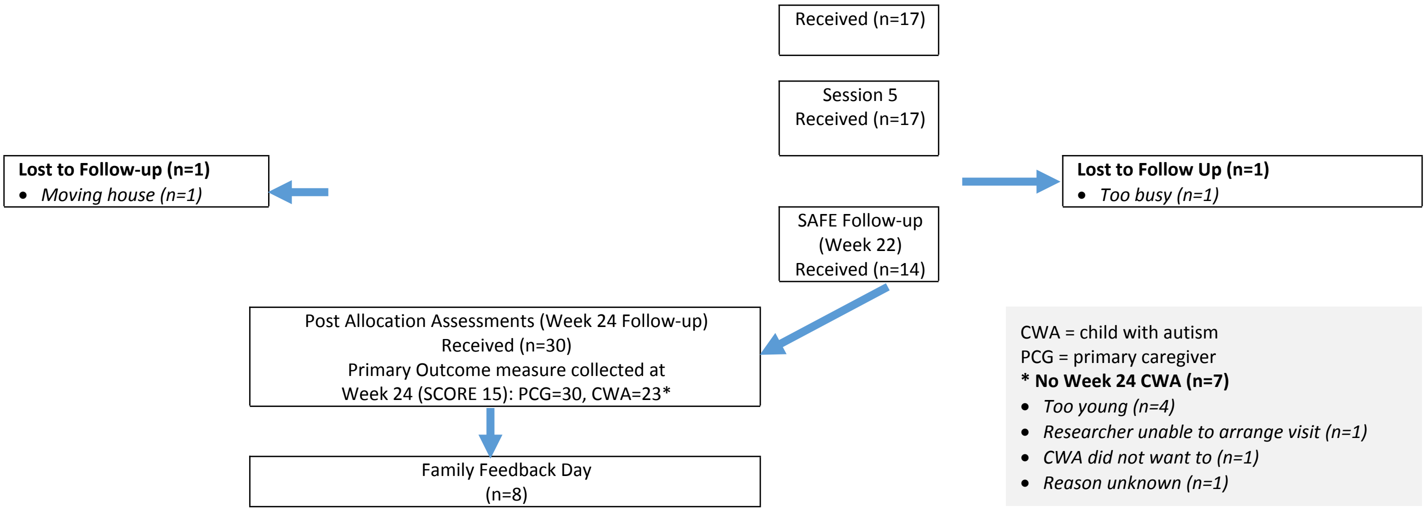

Figure 1. Participant Flow Diagram (CONSORT)

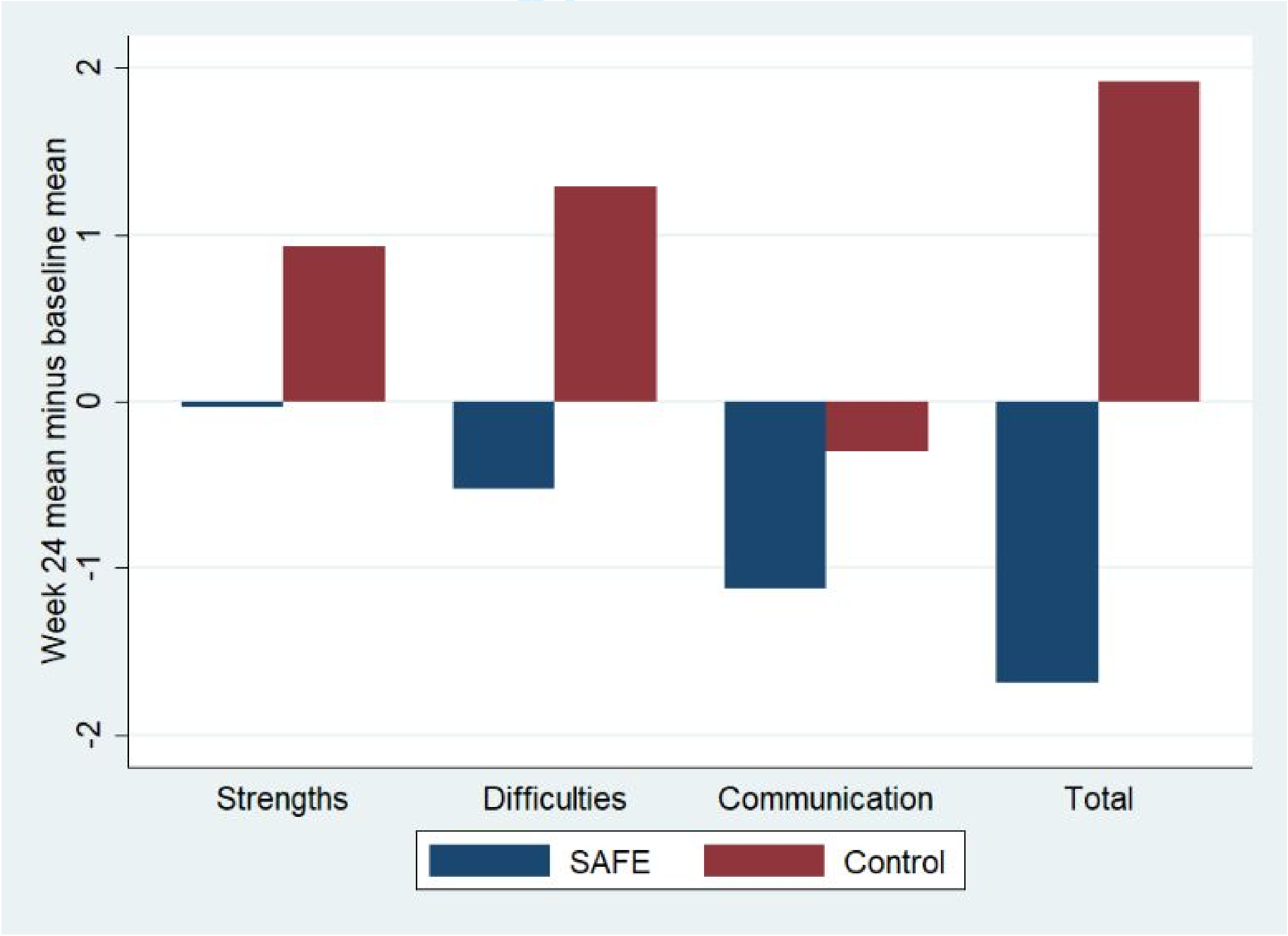

Figure 2. Mean change in SCORE-15 subcategories for SAFE+SUE (SAFE) and SUE only (Control)

For peer review only - http://bmjopen.bmj.com/site/about/guidelines.xhtml

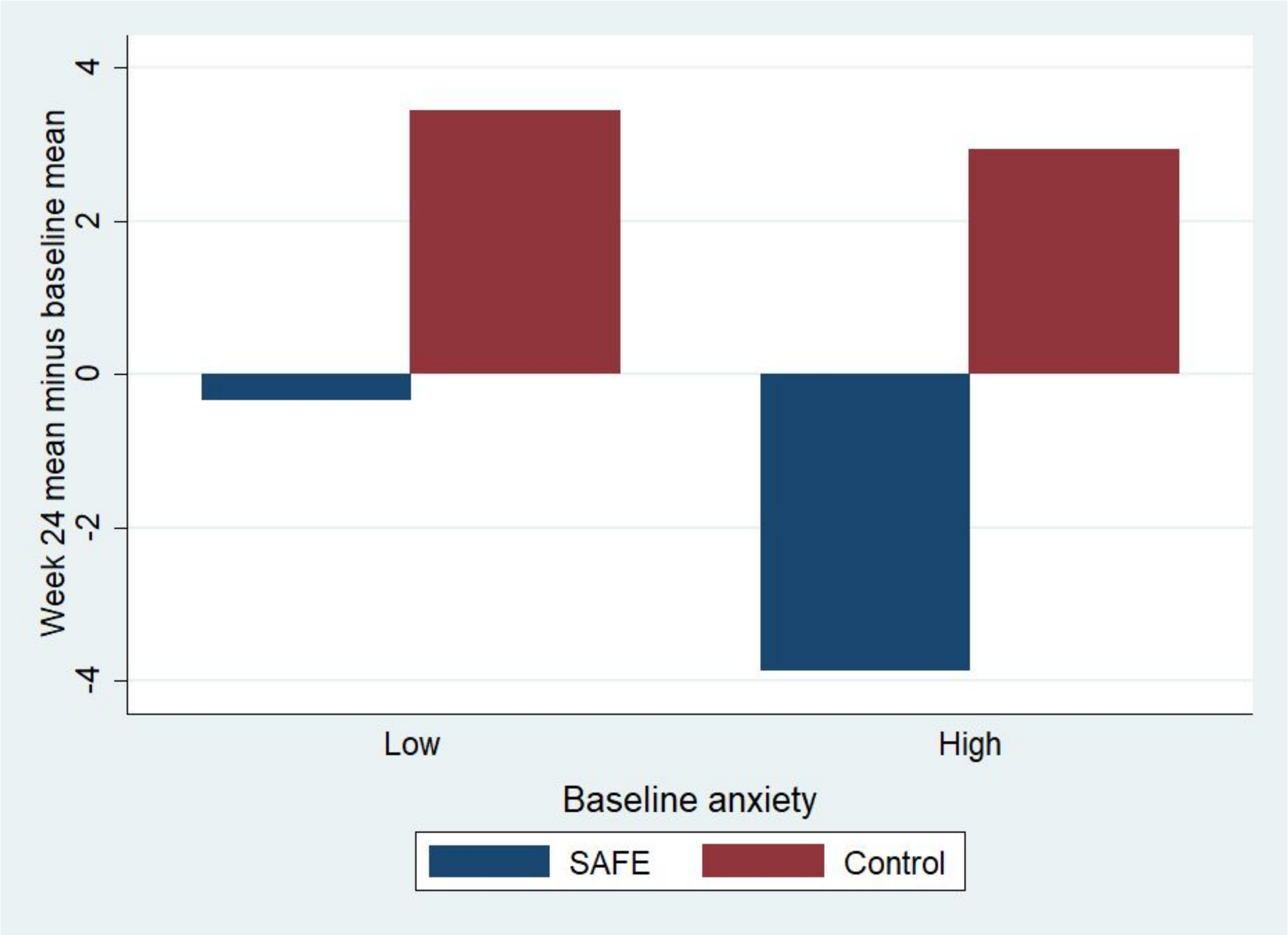

Figure 3. SCORE-15 total mean change for Primary Caregivers with low and high anxiety (a reduction = positive change; high anxiety > 7 on GAD-7)

review only

STUDY PROTOCOL

A multi-centred, randomised, controlled feasibility study comparing the SAFE intervention with support as usual for families of children with autism spectrum disorder

Systemic Autism-related Family Enabling (SAFE) study

Version 5

13th August 2018

Chief Investigator: Dr Rebecca McKenzie
University of Plymouth
Nancy Astor Building
Room 217
Plymouth
PL4 8AA

Study Sponsor: University Hospitals Plymouth NHS Trust
Research Office
Level 2, MSCP
Bircham Park Offices
1 Roscoff Rise
Plymouth
PL6 8FP

This protocol has regard for the HRA guidance

IRAS reference: 213527
REC reference: 17/SW/0192
ISRCTN: ISRCTN83964946

Sponsor reference: 17/P/064
Funder's reference: PB-PG-0815-20058 (NIHR RfPB)
Funder's reference: 7239 (Autistica)

For peer review only

TABLE OF CONTENTS

1 SIGNATURE PAGE 5
2 KEY CONTACT DETAILS 6
3 LIST OF ABBREVIATIONS 7
4 DEFINITIONS 8
5 STUDY SUMMARY 9
6 BACKGROUND AND RATIONALE 10
7 AIM AND OBJECTIVES 11
7.1 Aim 11
7.2 Objectives 11
8 TRIAL DESIGN 12
8.1 Study personnel 12
8.2 Figure 1: Study schema 13
9 STUDY OUTCOME MEASURES 13
9.1 Proposed primary outcome for main trial 14
9.2 Proposed secondary outcomes for main trial 14
10 PROCESS MEASURES 15
11 TRIAL PARTICIPANT SELECTION 15
11.1 Inclusion criteria 15
11.2 Exclusion criteria 16
11.3 Participant identification and recruitment 16
11.3.1 Participant identification 16
11.3.2 Recruitment pathways 16
11.3.3 Participant recruitment process 17
11.4 Consent 18
11.5 Baseline data capture (pre-allocation) 18
11.6 Table 1: Trial schedule 20
12 REGISTRATION, RANDOMISATION AND BLINDING 21
12.1 Registration 21
12.2 Randomisation 21
12.3 Blinding 21
13 TRIAL INTERVENTIONS 22
13.1 Post-allocation assessments (SAFE group only) 22
13.2 The SAFE intervention 22
13.3 Support as usually employed 23
14 FOLLOW-UP ASSESSMENTS 23
14.1 Week 24 post allocation assessments – all families 23
15 END OF THE STUDY 24
16 QUALITATIVE COMPONENT 24

For peer review only - http://bmjopen.bmj.com/site/about/guidelines.xhtml

16.1 Family focus group – experience of study participation (all families) ... 25
16.2 Family focus group – experience of SAFE (intervention only) ... 25
16.3 Family focus group – experience of SUE ... 25
16.4 Child focus group – experience of the study and SAFE and SUE ... 25
16.5 Therapist focus group ... 25
16.6 Interview - declined (all families) ... 25
16.7 Interview - withdrawn (all families) ... 26
16.8 Qualitative analysis ... 26
17 ECONOMIC EVALUATION ... 27
18 SAFETY REPORTING ... 27
18.1 Serious Adverse Events: definition and identification ... 27
18.2 Serious Adverse Events: reporting ... 28
19 PARTICIPANT WITHDRAWAL ... 28
20 DATA MANAGEMENT ... 28
20.1 Participant numbering ... 28
20.2 Data collection ... 29
20.3 Data entry ... 29
20.4 Source data ... 29
20.5 Case Report Form and questionnaire completion ... 29
20.6 Video and audio recorded data ... 29
20.7 Data confidentiality ... 29
20.8 Access to data ... 30
20.9 Archiving ... 30
21 STATISTICAL CONSIDERATIONS ... 30
21.1 Sample size ... 30
21.2 Statistical analysis ... 30
22 DATA MONITORING ... 31
22.1 Data monitoring plan ... 31
22.2 Quality assurance ... 32
22.3 Trial Management Group (TMG) ... 32
22.4 Trial Steering Committee (TSC) ... 32
22.5 Patient and Public Involvement (PPI) – Family Consultation Group ... 32
23 ETHICS APPROVAL ... 33
23.1 Protocol compliance ... 34
23.2 Notification of serious breaches of GCP and/or the protocol ... 34
24 STATEMENTS OF INDEMNITY ... 34
25 PROGRESSION TO A FULL TRIAL ... 34
26 DISSEMINATION POLICY ... 34
27 FINANCE ... 35
28 REFERENCES ... 35

For peer review only

29 APPENDICES 38
APPENDIX 1 – SAFE THERAPY SESSIONS 38
APPENDIX 2 – AMENDMENT HISTORY 41

For peer review only

For peer review only - http://bmjopen.bmj.com/site/about/guidelines.xhtml

1 SIGNATURE PAGE

Role	Name	Signature	Date
Chief Investigator	Dr Rebecca McKenzie		4th September 2018
Statistician	Professor Paul Ewings		4th September 2018
Sponsor's representative	Dr Chris Rollinson<br>(Research Governance Manager)<br>*on behalf of*<br>Corinna Mossop<br>(Sponsor's representative & RD&I Manager)		4th September 2018

For peer review only

For peer review only - http://bmjopen.bmj.com/site/about/guidelines.xhtml

2 KEY CONTACT DETAILS

**Chief Investigator**<br>Dr Rebecca McKenzie<br>Institute of Education<br>University of Plymouth<br>Nancy Astor Building<br>Plymouth, PL4 8AA<br>Tel: 01752 600600<br>Email: rebecca.mckenzie@plymouth.ac.uk	**Sponsor Representative**<br>Corinna Mossop<br>R&D Manager<br>University Hospitals Plymouth NHS Trust<br>R&D Office<br>Research Office<br>Level 2, MSCP, Bircham Park Offices<br>1 Roscoff Rise<br>Plymouth, PL6 8FP<br>Tel: 01752 431046<br>Email: corinna.mossop@nhs.net
**Funder (main study)**<br>National Institute for Health Research<br>NIHR Central Commissioning Facility<br>Grange House<br>15 Church Street<br>Twickenham<br>TW1 3NL<br>Tel: 02088 438057<br>Email: rfpb@nihr.ac.uk	**Funder (qualitative study)**<br>Autistica<br>St Saviour's House<br>39-41 Union Street<br>London, SE1 1SD<br>Tel: 02038 574340<br>Email: friends@autistica.org.uk
**Statistician**<br>Prof Paul Ewings<br>RDS South West<br>Research Office<br>The Old Building<br>Musgrove Park Hospital<br>Taunton, Somerset, TA1 5DA<br>Tel: 01823 342796<br>Email: paul.ewings@tst.nhs.uk	**Health Economist**<br>Dr Antonieta Medina-Lara<br>Health Economics Group<br>University of Exeter Medical School<br>St Luke's Campus<br>Heavitree Road<br>Exeter<br>EX1 2LU<br>Tel: 01392 726426<br>Email: a.medina-lara@exeter.ac.uk
**Lead Qualitative Researcher and Therapy Supervisor**<br>Prof Rudi Dallos<br>School of Psychology<br>8-11 Kirkby Place<br>University of Plymouth<br>Drake Circus<br>Plymouth, PL4 8AA<br>Tel: 01752 586656<br>Email: r.dallos@plymouth.ac.uk	
**Trial Manager**<br>Dr Helen Hancocks<br>Peninsula Clinical Trials Unit<br>N16, ITTC Building 1<br>Plymouth Science Park<br>Plymouth PL6 8BX<br>Tel: 01752 315252<br>Email: helen.hancocks@plymouth.ac.uk	**Assistant Trial Manager**<br>Elinor Pegg<br>Peninsula Clinical Trials Unit<br>N16, ITTC Building 1<br>Plymouth Science Park<br>Plymouth PL6 8BX<br>Tel: 01752 439830<br>Email: elinor.pegg@plymouth.ac.uk

For peer review only - http://bmjopen.bmj.com/site/about/guidelines.xhtml

3 LIST OF ABBREVIATIONS

**ADHD**	Attention Deficit Hyperactivity Disorder
**APA**	American Psychiatric Association
**APSA**	American Political Science Association
**ASD**	Autism Spectrum Disorder
**ASDAT**	Autism Spectrum Disorder Assessment Team
**ASI**	Area of Special Interest
**BSA**	Between Session Activity
**CAMHS**	Child and Adolescent Mental Health Services
**CBCL**	Child Behaviour Checklist
**CDC**	Child Development Centre
**CGHQ**	Caregiving Helplessness Questionnaire
**CHU-9D**	Child Health Utility 9 Dimensions
**CI**	Chief Investigator
**COS**	Circle Of Security Intervention
**CRF**	Case Report Form
**CSQ**	Client Satisfaction Questionnaire
**CTU**	Clinical Trials Unit
**CWA**	Child With Autism
**CYP**	Children & Young People
**DSM**	Diagnostic and Statistical Manual of Mental Disorders
**EQ-5D**	EuroQoL 5 Dimensions 5 Levels
**FGD**	Focus Group Discussion
**GP**	General Practitioner
**HAT**	Helpful Aspects of Therapy questionnaire
**IRAS**	Integrated Research Application System
**ISRCTN**	International Standard Randomised Controlled Trial Number
**NHS**	National Health Service
**NICE**	National Institute for Health and Care Excellence
**NIHR**	National Institute for Health Research
**PDI**	Parent Development Interview
**PenCTU**	Peninsula Clinical Trials Unit
**PHQ-SADS**	Patient Health Questionnaire – Somatic Anxiety Depressive Symptoms
**PPI**	Patient and Public Involvement
**QRA**	Qualitative Research Assistant
**QALY**	Quality Adjusted Life Year
**RA**	Research Assistant
**RCT**	Randomised Controlled Trial
**RCHT**	Royal Cornwall Hospitals NHS Trust
**REC**	Research Ethics Committee
**RFQ**	Reflective Functioning Questionnaire
**SAE**	Serious Adverse Event
**SAFE**	Systemic Autism-related Family Enabling
**SAM**	Self Autism Mapping
**SCORE**	Systemic CORE 15
**SN**	Specialist Nurse
**SUE**	Support as Usually Employed
**TCQ**	Training Checklist & Questionnaire
**TMG**	Trial Management Group
**TSC**	Trial Steering Committee

For peer review only

For peer review only - http://bmjopen.bmj.com/site/about/guidelines.xhtml

4 DEFINITIONS

Autism Spectrum Disorder (ASD)

For the purposes of the SAFE study Autism Spectrum Disorder (ASD) will be defined as a diagnosis according to the diagnostic and statistical manual of mental disorders, 5th edition: DSM-5 (edited by American Psychiatric Association, 2013)[1].

Family

The SCORE family assessment instrument defines a family as any group of people who care about each other and define themselves as such. For the purposes of this study, all references to 'family' mean those family members who are willing to participate in the study assessments and who would be willing to consent to take part in family therapy sessions (if randomised to the intervention group). The SAFE study will orient people towards thinking of household members, but invite them at baseline (prior to randomisation) to choose who they want to invite to consent to be included in study assessments and therapy sessions (if applicable). This will typically include the parent(s) of the child with autism spectrum disorder (henceforth Child With Autism, CWA), the CWA, the sibling(s) and additional family members that have a significant level of involvement with the CWA. The minimum number of family members that will be required to be present for a family therapy session (if the family is allocated to the intervention) is one parent and the CWA.

For peer review only - http://bmjopen.bmj.com/site/about/guidelines.xhtml

5 STUDY SUMMARY

**Study title**	A multi-centred, randomised, controlled feasibility study comparing the SAFE intervention with support as usual for families of children with autism spectrum disorder.
**Study design**	A randomised, controlled feasibility study.
**Study participants**	36 families of children with autism spectrum disorder (ASD).
**Intervention**	Systemic Autism-related Family Enabling (SAFE) + support as usually employed for 16 weeks.
**Control**	Support as Usually Employed (SUE) for 16 weeks.
**Follow-up**	24 weeks (±2 weeks) post-allocation.
**Setting**	University Hospitals Plymouth NHS Trust, Royal Cornwall Hospitals NHS Trust, and Cornwall Partnership NHS Foundation Trust. Community advertising.
**Aim**	To establish the feasibility of a definitive randomised controlled trial of SAFE for families of children with ASD.
**Objectives**	• Demonstrate ability to identify, recruit and randomise eligible families.<br>• Verify proposed outcome measures and follow-up schedule are acceptable to participants, and targets for loss to follow-up are achievable.<br>• Assess adherence of therapists and families to the intervention.<br>• Gather quantitative outcome data to inform design & sample size of future trial.<br>• Adapt existing resource use questionnaires, and assess the feasibility of preference-based instruments for this population, to facilitate a future economic evaluation alongside the trial.<br>• Collect data on families' experience of SAFE intervention and the study itself.<br>• Ensure that the training arrangements are effective and scalable, both for a larger RCT, and for adoption within the NHS.<br>• Develop the operational experience required to manage a future trial.
**Main outcomes**	Primary outcome measure (proposed for main trial):<br>• Global family function (SCORE 15)<br>Secondary outcome measures (proposed for main trial):<br>• Child behaviour (Child Behaviour Checklist, CBCL)<br>• Child-parent attachment (Coding of Attachment-Related Parenting for use with children with Autism - CARP-A)<br>• Anxiety & depression (Patient Health Questionnaire - PHQ-SADS)<br>• Reflective Functioning (RFQ)<br>• Caregiving Helplessness Questionnaire (CGHQ)<br>• Economic measures (EQ-5D-5L, CHU-9D, and resource use questionnaire)
**Inclusion criteria**	• Family includes child with ASD, aged 3-16 years<br>• Diagnosis of ASD, severity level 1 or 2<br>• Diagnosed within 12 months of consenting to the study<br>• If other diagnoses are present, ASD must be primary diagnosis<br>• Family are willing to comply with study requirements
**Exclusion criteria**	• Child with ASD severity level 3<br>• Child with ASD and intellectual impairment<br>• Serious concomitant illness in child or family, or other circumstances such that they are unable to comply with study requirements<br>• Families who may be a risk to safety of research staff<br>• Insufficient English or capacity for parent/child to consent/assent to study.
**Study timelines**	Set-up 4 months, recruitment, intervention and follow-up 14 months, analysis & write-up 6 months. 24 months total study duration.
**End of study**	Date that last family has completed the qualitative feedback.

For peer review only

For peer review only - http://bmjopen.bmj.com/site/about/guidelines.xhtml

6 BACKGROUND AND RATIONALE

More than 1% of the UK population has a diagnosis of autism and numbers are rising. Families of children with autism (CWA) present complex needs. Children with autism have impairments in social interaction, communication, and behavioural flexibility. Autism is widely accepted to have a genetic component and the Broad Autism Phenotype is disproportionally represented among family members [2]. Mental health problems are experienced by more than 70% of individuals with autism and more than 50% of their parents [3,4]. Individuals with autism suffer from high levels of anxiety and depression compared to those with other developmental conditions [5,6,7]. Families of children with autism have higher rates of depression, anxiety, and social phobia, than families with typically developing children, or children with other developmental disorders [8]. Parents of children with autism are more likely to be hospitalised for mental disorders than parents of typical children [9]; and mothers of children with autism are reported to have higher unmet needs, more difficulties coping, and lower satisfaction with service interactions than mothers of children with other disabilities [10].

As families of children with autism often exhibit psychological morbidity alongside autism, even conservative estimates for costs of services to treat these problems exceeds £4 billion per annum shared amongst 700,000 families [11,12]. Furthermore, untreated or unresponsive mental health problems impose societal costs making it hard for parents to interact effectively with services [13], worsening outcomes for children, and exacerbating the substantial economic burden of autism.

Explanations for high levels of affective disorders in these families include: stress associated with the condition of autism, genetic factors, and intergenerational family dynamics. Parenting children with autism involves stresses associated with maladaptive behaviour, lack of empathy, and atypical attachment behaviour displayed by children [14]. Studies exploring the medical histories of family members indicate, however, that onset of affective disorders predate the birth of the child [8,15,16] suggesting that mental health difficulties cannot be wholly accounted for by stress involved in parenting. Depression and anxiety among family members have been tentatively linked to genetic factors independent of the Broad Autism Phenotype [17]. Few studies explore the intergenerational presence of affective disorder associated with autism and more work is needed in this area [8,15,16]. In particular, there are few studies, which question whether affective disorders combined with autistic traits among parents, pose an environmental risk for the development of autistic symptoms in children. Limited work demonstrates, however, that a child's risk of developing autism is doubled if both parents have a personality or psychiatric disorder [9]; and parents of children with autism report that lack of psychological wellbeing exacerbates maladaptive behaviour in their children [18]. A related body of research exists in the attachment literature, which suggests that emotional problems displayed by the parents of children with autism may be trans-generationally transmitted through insecure attachment patterns in families. Our initial work [19] indicates that these parents have frequently experienced high levels of trauma and subsequent mental health problems. Unresolved trauma is known to impede parenting abilities and is associated with the development of severe forms of pathology in children.

Autistic symptoms, affective disorders and coping skills are interrelated on a variety of levels [15]. Many parents of children with autism also have parents with autistic traits and affective disorders; and this has implications for their own parenting models. For example, it has been suggested that difficulties in interpreting the needs of infants characterise very early attachments between children with autism and their parents, which initiate potentially difficult family relationships and contribute to autistic symptomology [20]. Poor communicative cues among children with autism [21] are also thought to arouse less insight and reflection among parents in narratives about their children, compared to parents of typical children or children with other developmental difficulties [22]. Hence, parents of children with autism may have difficulties responding to their children's needs. They may also

For peer review only - http://bmjopen.bmj.com/site/about/guidelines.xhtml

encounter difficulties communicating needs to external agencies [23], which may trigger existing tendencies for negative affect. This is borne out by studies, reporting that families of children with autism are often characterised by a palpable air of tension [24]. Families of children with autism represent a high-risk group, yet treatment for these families is disjointed, costly and inadequate [25,26].

A more joined-up approach is required which focuses on autism related need, coping with maladaptive behaviour and mental health difficulties by encouraging fundamental reflective functioning and improving family dynamics. The SAFE study should be placed in the context of NICE guidelines and recommendations [27,28] as well as developments regarding children's service provision proposed by the Munroe Report [29,30], and the 'Future in Mind' children and young people's mental health report [31]. The SAFE study also reflects recommendations by other researchers working in the field [32,33]. Families of children with autism themselves highlight the importance of professionals working therapeutically with children and the wider family, in contrast to parents of children with conditions such as Down Syndrome who tend to stress the support needs of their child within educational and community settings [10].

SAFE is a systemic family therapy approach designed by experts to address autism related needs including mental health difficulties and problematic behaviour. More than 90 families have contributed to the development of SAFE. Systemic Family Therapy is a well-recognized, evidence-based psychotherapeutic approach [34], which is recommended treatment for conditions such as Conduct Disorder, ADHD and Anorexia Nervosa [35]. Despite evidence that family therapies can provide benefits to CWA and their parents [36,37] its efficacy for treating this condition has not been subject to a randomised controlled trial. A comprehensive search of clinical trial registries revealed no on-going trials assessing Systemic Family Therapy as a treatment for autism and associated mental health problems. This is surprising given guidelines and recommendations for care; the successful use of family therapies for a range of conditions and reports documenting key areas of concern for the UK autism community [38,39], which overwhelmingly show that families of CWA want interventions which make real improvements to their daily life and sense of wellbeing.

To be confident that SAFE is an effective and cost-effective intervention for families of children with autism, a large multi-centre trial is planned. Before this can happen, there are important uncertainties that need to be addressed. This feasibility study, if successful, will help to inform a larger, randomised controlled, multi-centre, nationwide trial.

7 AIM AND OBJECTIVES

7.1 Aim

The overarching aim of this study is to establish the feasibility of a definitive randomised controlled trial of Systemic Autism-related Family Enabling (SAFE) therapy for families of CWA.

7.2 Objectives

The study objectives are to:

i) Demonstrate ability to identify, recruit and randomise eligible families.

ii) Assess whether proposed outcome measures and follow-up schedule are acceptable to participants, and whether targets for loss to follow-up are achievable.

iii) Assess adherence of therapists and families to the intervention.

iv) Gather quantitative data on outcomes to inform the design (and sample size) of a future trial.

v) Adapt existing resource use questionnaires and assess the feasibility of preference-based instruments for this population to facilitate a future economic evaluation alongside the trial.

For peer review only - http://bmjopen.bmj.com/site/about/guidelines.xhtml

vi) Collect data on the families' experience of the SAFE intervention and of the study itself.

vii) Ensure that training arrangements are effective and scalable for a larger RCT and for adoption within the NHS.

viii) Develop the operational experience required to manage a future trial.

8 TRIAL DESIGN

This is a randomised, controlled, multi-centred feasibility study including children with ASD and their families. Thirty-six families will be randomised in a 2:1 ratio to receive support as usually employed (SUE) plus a programme of Systemic Autism-related Family Enabling (SAFE) therapy, or SUE alone, for a period of 16 weeks. Outcome assessors will be blinded to allocation. All participants will be followed up at 24 weeks post-allocation (± 2 weeks) via a face-to-face visit, hence each family will participate in the SAFE study for approximately eight months. The end date for the trial will be the date on which the last family completes the qualitative feedback. An embedded qualitative study will collect information about the feasibility and acceptability of the intervention and the study itself. A schematic diagram of the trial is given in Figure 1.

8.1 Study personnel

There will be research assistants in this study, split between the centres.

Study therapists will be involved in the study, at each centre. The therapists will be centre specific to provide continuity for participating families. Therapists will be present at every SAFE therapy session.

All study personnel undertaking home visits will be asked to comply with their employers lone working policy, and will be required to have a Disclosure and Barring Service (DBS) check before working with children.

For peer review only

For peer review only - http://bmjopen.bmj.com/site/about/guidelines.xhtml

8.2 Figure 1: Study schema

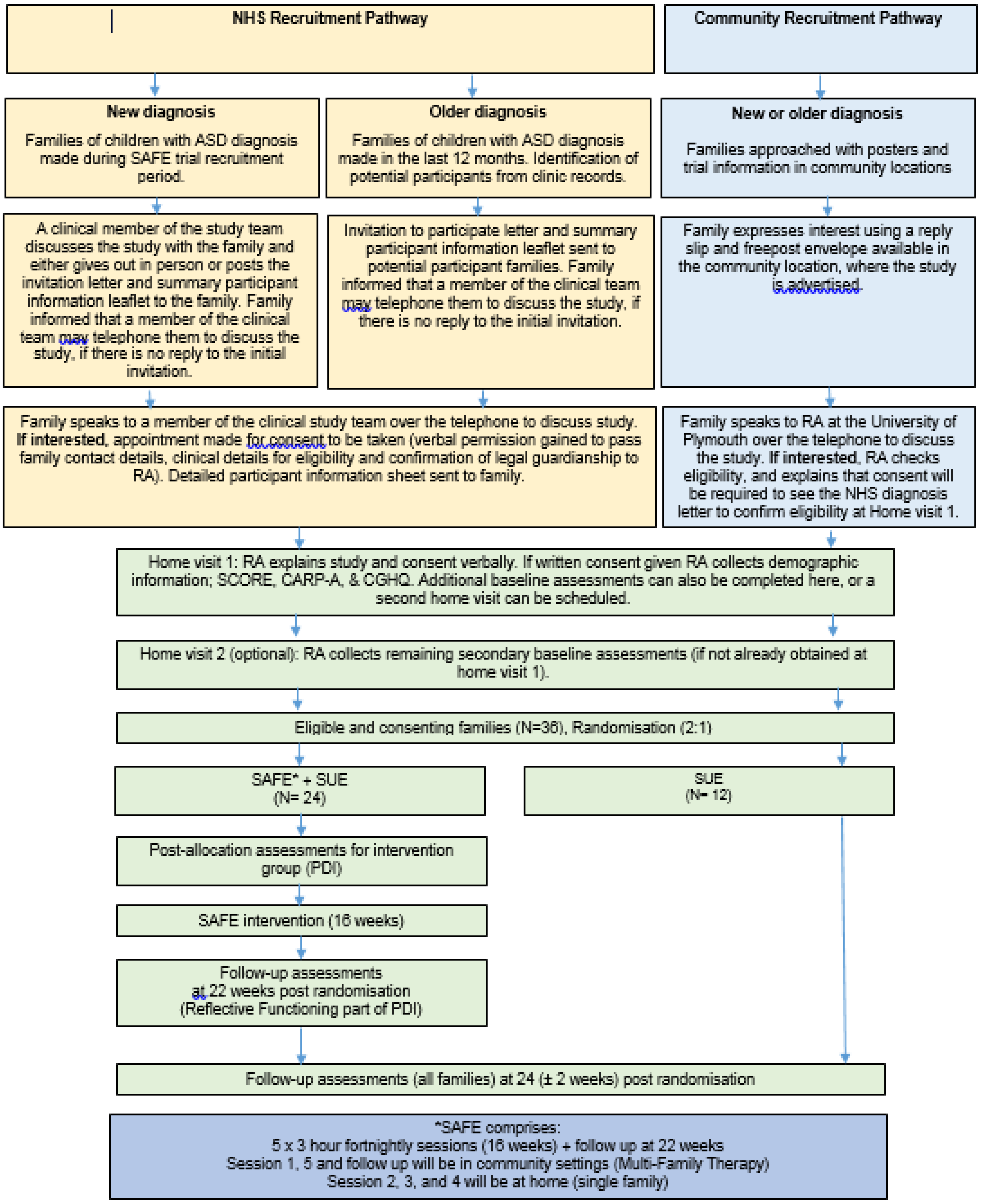

9 STUDY OUTCOME MEASURES

The suggested primary and secondary outcomes for a proposed definitive RCT are described below and will be administered to all families at baseline and 24 weeks (± 2 weeks) post-allocation.

Predicted change in the intervention group involves:

1. Improvements in overall family function
2. Reduced levels of psychological distress in parents
3. Improved attachment relationships between children and parents
4. Reduced levels of maladaptive behaviour in children
5. Improved understanding of self and others
6. Consequent improved sense of parenting competency

For peer review only - http://bmjopen.bmj.com/site/about/guidelines.xhtml

9.1 Proposed primary outcome for main trial

The proposed primary outcome for the main trial is **overall family function** (relating to prediction 1 above) measured with the Systemic CORE 15 (SCORE) [40]. This is a 15 item paper-based survey, which is a reliable, valid index of family functioning, and takes approximately 20 minutes to complete. SCORE is the primary measure of family functioning employed in CYP (Children and Young People's) Improving Access to Psychological Therapies national programme, and is the gold standard for assessing the impact of family therapy on quality of life in the UK[41]. Every able family member should complete the SCORE, and the same family members should complete the SCORE at baseline and 24 weeks.

9.2 Proposed secondary outcomes for main trial

The proposed secondary outcomes for a main trial index changes in child behaviour, child-parent attachment, anxiety, and depression. Measures of quality of life/health utility are included to support the economic evaluation of SAFE.

- Patient Health Questionnaire – Somatic Anxiety Depressive Symptoms (PHQ-SADS). This comprises the PHQ-9 measuring depression and the GAD-7 measuring **anxiety**[42] i.e. the PHQ-15 is not used. These are short, self-report questionnaires, sensitive to change, widely used in research and clinical practice, and with established correspondence to clinical diagnoses (prediction 2 above). The paper-based questionnaires relate to the parent(s) own health and should be completed at both baseline and 24 weeks. With 16 items in total, this takes approximately 10 minutes to complete. If both parents are participating, they should each complete a copy of the questionnaire; if not, then a single parent should complete it.

- Child-parent attachment (Coding of Attachment-Related Parenting for use with children with Autism - CARP-A) [43]. The CARP-A is an observational measure of **child's attachment behaviour towards their carer** (prediction 3 above), designed to provide a naturalistic measure of attachment behavior in children with developmental disorders, specifically children with ASD. An observational measure which involves a short play task between the child with ASD and their parent or carer, it takes approximately 10 minutes to complete, and will be video recorded. This should be completed by the RA, for the child with ASD and their parent or carer, at baseline and 24 weeks.

- The Child Behaviour Checklist (CBCL) [44]. This is a 30-item paper-based survey which detects emotional and **behavioural problems** (prediction 4 above) in children. One parent should complete the CBCL at both baseline and 24 weeks (this should be the same parent at both time points). Completion should take approximately 10 minutes on each occasion.

- The Reflective Functioning Questionnaire (RFQ) [45] measures **ability to understand own and others' mental states** (prediction 5 above). Impairments are implicated in a variety of psychological problems. This 8-item questionnaire should be completed at both baseline and 24 weeks, taking approximately 5 minutes to complete on each occasion. If both parents are participating, they should each complete a copy of the questionnaire; if not, then a single parent should complete it.

- Caregiving Helplessness Questionnaire[46] (CGHQ). This is a 26-item questionnaire designed to assess aspects of disorganised caregiving such as caregiving helplessness, role reversal, and frightened/frightening caregiving experiences (prediction 6 above). The CGHQ should be completed at both baseline and 24 weeks. If both parents are participating, they should each complete a copy of the questionnaire; if not, then a single parent should complete it.

For peer review only - http://bmjopen.bmj.com/site/about/guidelines.xhtml

Proposed health economic outcome measures:

- EuroQol 5 dimensions (EQ-5D-5L). This is a standardised generic instrument for measuring health outcome. A paper-based survey consisting of 5 items, it takes approximately 5 minutes to complete. If both parents are participating, they should each complete a copy of the questionnaire; if not, then a single parent should complete it, in relation to his/her own health.

- Child Health Utility 9 Dimensions (CHU-9D). The CHU-9D is a paediatric generic preference based measure of health related quality of life. One child or parent (for children ≤ 5 years) from each family will complete this nine item paper-based survey, which takes approximately 10 minutes. If parent-completed, ideally the same person will complete each time.

- Resource Use Questionnaire. A paper-based questionnaire that takes approximately 10 minutes to complete. One parent from each family will complete this in relation to his/her child use of health care and social resources.

10 PROCESS MEASURES

Families in the intervention arm and study therapists will complete a number of self-report questionnaires at the end of each SAFE therapy session. These are intended to capture information on the implementation of SAFE within the trial, specifically:

- Client Satisfaction Questionnaire (CSQ-8). This is a paper-based survey consisting of eight items and takes approximately 8 minutes to complete. The CSQ-8 is widely used in the measurement of client/patient assessment of satisfaction with services/clinical care. If both parents are participating they should each complete a copy of the questionnaire, but if not then a single parent should complete. The questionnaire should be completed after each therapy session.
- Training Checklist and Questionnaire (TCQ). This is a paper-based survey consisting of 10 items and takes approximately 10 minutes to complete. The TCQ will be completed by study therapists after each session and measures intervention protocol adherence including perceived competence, additional training needs, and perceived effectiveness of techniques and ease of delivery.
- Helpful Aspects of Therapy Questionnaire (HAT). This is a paper-based questionnaire consisting of 5 open-ended questions, taking approximately 10 minutes to complete. Each able family member, including children, will complete HAT, after each therapy session. HAT is widely used to measure the process of change over therapy sessions. Participants are asked to describe in their own words the most helpful event in the session, and to rate how helpful it was. They are also asked about other helpful or hindering events in the session.

11 TRIAL PARTICIPANT SELECTION

11.1 Inclusion criteria

Potential participant families must satisfy the following criteria to be enrolled in the study:

- Family includes child with ASD, aged 3-16 years
- Diagnosis of ASD, severity level 1 or 2
- Diagnosed within 12 months of consenting to the study
- If other diagnoses are present, ASD must be primary diagnosis
- Family are willing to comply with study requirements

For peer review only - http://bmjopen.bmj.com/site/about/guidelines.xhtml

11.2 Exclusion criteria

Potential participants meeting any of the following criteria will be excluded from study participation:

- Child with ASD severity level 3
- Child with ASD and intellectual impairment
- Serious concomitant illness in child or family, or other circumstances such that they are unable to comply with study requirements
- Families who may be a risk to safety of research staff
- Insufficient English language, or capacity for parent/child to consent/assent to the study.

11.3 Participant identification and recruitment

11.3.1 Participant identification

Participants will be identified from either NHS or community locations. Potential participants will be approached in a variety of different ways depending upon the circumstances. This may include, but is not limited to post, in-person, email, text message, posters, leaflets, newspaper advertisements, and online social media.

11.3.1.1 *NHS sites*

Participants will be identified from three study centres; University Hospitals Plymouth NHS Trust Child Development Centre, Cornwall Partnership NHS Foundation Trust Autism Spectrum Disorder Assessment Team (ASDAT), and Royal Cornwall Hospitals NHS Trust (RCHT).

University Hospitals Plymouth NHS Trust Child Development Centre is commissioned to provide Child Development services for children and young people residing in Plymouth. Diagnostic pathways are in place for pre-school and school-age children suspected of being on the autistic spectrum.

ASDAT is a multi-agency, multi-professional team providing an assessment pathway for children in Cornwall (up to 18 years) presenting with social communication difficulties, many of whom have ASD.

The paediatric team at RCHT are involved in the diagnosis of autism in pre-school age children.

11.3.1.2 *Community*

Participants that have received either a new diagnosis, or a diagnosis within the last 12 months will be approached through community groups, using a recruitment poster, invitation letter, reply slip, participant information leaflet, and freepost envelope. These participants will not necessarily have received their diagnosis from the NHS sites listed in section 11.3.1.1 above. These participants will be asked by a member of the local research team at the University of Plymouth to consent to providing the original NHS diagnosis letter, which will be used to determine eligibility to participate in the study, and legal guardianship at home visit 1.

11.3.2 Recruitment pathways

11.3.2.1 *NHS sites*

The pathways used to identify and recruit families in NHS sites will vary according to local practice, and the needs of the individual families being approached. Initial identification will depend upon the timing of the diagnosis of the CWA; some families will receive a diagnosis during the SAFE recruitment period (new diagnosis), and others will have been diagnosed before the SAFE study recruitment period starts (older diagnosis).

For peer review only - http://bmjopen.bmj.com/site/about/guidelines.xhtml

Families with a diagnosis during the SAFE recruitment period will be identified as potentially eligible for the study (and initial approach made) by the diagnosing paediatrician or another suitable member of the clinical team.

Families with a diagnosis before the SAFE study recruitment period (older diagnosis) will be identified as potentially eligible from clinic records by a suitably qualified member of the clinical team at each centre (e.g. research nurse).

11.3.2.2 *Community*

Families who express interest from the recruitment poster will be directed to the local research team at the University of Plymouth. Consent for the RA to use the NHS letter of diagnosis will be taken in order to confirm eligibility and legal guardianship at home visit 1.

11.3.3 Participant recruitment process

Where possible, for continuity, clinical staff who have had prior contact with the family during the diagnostic pathway will be involved in the recruitment process for the study. Initial contact with families, prior to consent being taken, will be through the local clinical team, such as the diagnosing paediatrician, or a specialist nurse who is already known to potential participants.

Participants will be recruited in four cohorts of 9 families, 36 families, in total. Once the full complement of nine families per cohort has been recruited, randomisation can proceed (see section 12).

11.3.3.1 Initial information

All potential participant families will receive a participant information leaflet including an invitation to take part. This will either be given out in person, posted or emailed to the potential participant family by a clinical member of the local team, or RA if the participant expresses interest through a community recruitment pathway. The participant information leaflet will contain information about the study in plain English. Parents will be asked to explain the information to younger children in a way that is appropriate for their child, and suggestions for how to do this will be contained in the leaflet.

The leaflet will contain contact details for a member of the local clinical team, such as a specialist nurse for participants recruited through the NHS. All interested families recruited from NHS sites will therefore be able to speak to a member of the clinical team to discuss the study and have any questions answered. For participants recruited through the NHS a member of the clinical team will telephone participants if they do not respond to the initial invitation letter. Families recruited through the community will be given contact details for the RA at the University of Plymouth, who will be able to discuss the study in more detail with them, and where possible, indicate a clinician that the family can discuss the study with.

11.3.3.2 Telephone contact and home visit

For the NHS recruitment pathway described above, if the potential participant family expresses interest by telephone they will be asked to confirm that they are happy to have their contact details, clinical details for eligibility and confirmation of legal guardianship forwarded to a member of the study team at the University of Plymouth, such as the RA and/or QRA, so that the consent process can be arranged.

A checklist will be provided to the clinical team to ensure that verbal consent to forward these details to the research team at the University of Plymouth is formally documented.

For the community recruitment pathway described above interested families will return the reply slip for the study, which gives written consent from the family to be contacted by the research team. The RA will complete a form with the family that gives consent for the RA to view the NHS letter of

For peer review only - http://bmjopen.bmj.com/site/about/guidelines.xhtml

diagnosis at home visit 1, to confirm eligibility and legal guardianship.

During the telephone call the first home visit (section 11.5 below) will be arranged by a member of the study team for those who express interest in participating. Consent will be taken at the first home visit one (described below).

During the telephone call, any families who have declined to participate will be asked if they would be happy to participate in an interview, as part of the qualitative part of the study (described in section 15.6).

Following the telephone call and before the consent meeting a member of the study team will send the detailed participant information sheet to all interested families. Ample time will be provided for the family to be able to read and discuss the information sheet ahead of the consent meeting.

The first home visit will be conducted by two research assistants (RA and qualitative RA), or other members of the study team, suitably trained to answer questions about the study, at the family home. If the family is eligible and willing to participate in the study, the RAs will obtain written consent at this visit from relevant family members (see section 11.4), and undertake the baseline assessments. All of the baseline assessments can be completed at home visit one, but if the family prefer, some of the assessments (SCORE, CARP-A and CGHQ) should be completed as a priority, and the remainder completed at a second home visit. It is important that all of the family members who will be participating in the study attend the consent meeting and complete the baseline assessments.

A screening log must be maintained at each centre to document all participants considered for the trial, including those subsequently excluded, to enable population of a CONSORT flowchart for the study. Where possible, the reason for non-entry to the trial should be documented.

11.4 Consent

As described above, all potential participant families will be provided with a participant information leaflet, and a more detailed participant information sheet, and allowed sufficient time for consideration of participation. The study information may be given out in person or sent by post or email depending on individual circumstances. Written, informed consent will be obtained at the first home visit by the RAs, or other suitably trained member of the study team, once the details and implications of the study have been explained, and families have had the opportunity to ask questions. Consent must be obtained prior to any study procedures being undertaken.

Parent(s)/carer(s) will be asked to complete a consent form for themselves and also give consent for the child with ASD, and any additional siblings that are <16 years of age. Additional adult family members will be asked to sign a consent form to participate in the study. Children who are <16 will be given the option of completing a separate assent form if they wish. As part of the consent process, families will be asked to agree to the GP of their child with ASD being informed of their involvement in the study.

A pseudo-anonymised copy of the completed consent/assent form must be forwarded to CTU for central monitoring.

11.5 Baseline data capture (pre-allocation)

The trial schedule is given in Table 1.

Home visit 1 (approximately 2 hours): Following receipt of valid informed consent/assent, the RAs or other members of the research team will record relevant family history and demographic details on a study specific case report form (CRF). At the same visit, the RAs will support all study families to complete the following baseline assessments:

- Systemic CORE 15 (SCORE)

For peer review only - http://bmjopen.bmj.com/site/about/guidelines.xhtml

- Coding of Attachment-Related Parenting for use with children with Autism (CARP-A)
- Caregiving helplessness Questionnaire (CGHQ)

If it is not possible to complete all of the baseline assessments in the first home visit the RAs will then arrange a second home visit with the family.

Home visit 2 (approximately 2 hours) – if required:
In a second home visit the RAs will support the families to complete:

- Child Behaviour Checklist (CBCL)
- Patient Health Questionnaire – Somatic Anxiety Depressive Symptoms (PHQ-SADS)
- Reflective Functioning Questionnaire (RFQ)
- EuroQoL 5 Dimensions 5 Levels (EQ-5D-5L)
- Child Health Utility 9 Dimensions (CHU-9D)
- Resource Use Questionnaire

All of the above baseline data will be captured prior to randomisation. All baseline measures should be completed at one or two baseline visits, and the questionnaires returned by the RAs to the Clinical Trials Unit (CTU), according to an agreed procedure. Randomisation may be delayed if baseline assessments have not been received by CTU.

For peer review only

For peer review only - http://bmjopen.bmj.com/site/about/guidelines.xhtml

11.6 Table 1: Trial schedule

		Baseline assessments (pre-allocation) 2 home visits	Day 0	Post-allocation assessments	SAFE intervention sessions Week 1 - 16					Week 22	Week 24
					SAFE 1 Group setting (3h)	SAFE 2 Home setting (3h)	SAFE 3 Home setting (3h)	SAFE 4 Home setting (3h)	SAFE 5 Group setting (3h)	SAFE follow-up Group setting (3h)	Post-allocation assessments
**All families**											
**Whole family**	Informed consent	X									
	Randomisation		X								
	Family history	X									
	Demographics	X									
	SCORE	X									X
**Parent**	CBCL	X									X
	PHQ-SADS	X									X
	RFQ	X									X
	CGHQ	X									X
	EQ-5D-5L	X									X
	CHU-9D	X									X
	Resource use questionnaire	X									X
	CARP-A (observational)	X									X
**Child**	CARP-A (observational)	X									X
	CHU-9D	X									X
**SAFE intervention families only**											
**Whole family**	HAT				X	X	X	X	X		
	BSA				X	X	X	X	X		
	SAFE follow-up session									X	
**Parent**	PDI (full interview)			X							
	PDI (mentalisation)								X	X	
	CSQ-8				X	X	X	X	X		
**Therapists**											
**Therapist**	TCQ				X	X	X	X	X		

For peer review only

For peer review only - http://bmjopen.bmj.com/site/about/guidelines.xhtml

12 REGISTRATION, RANDOMISATION AND BLINDING

12.1 Registration

Following consent, the RA or other suitably trained member of the study team will enter the study centre, name, address and contact details of the consented family onto the SAFE study database. It is anticipated that it may take 8-9 weeks to recruit and register nine families at a single centre.

12.2 Randomisation

The study aims to enrol four groups of nine families over the recruitment period, hence the randomisation procedure will be performed four times in all. Once nine families have consented at a study centre (Plymouth or Cornwall) and completed the baseline assessments they will be randomised en bloc to receive SUE + SAFE or SUE alone in a 2:1 (SUE + SAFE: SUE) ratio. The requirement for there to be nine families in each cohort at each centre may need to be flexible depending on the recruitment situation at each centre. The minimum number of families required in a cohort before randomisation can take place is six and the maximum is twelve, allowing an even number of families to be allocated to the SAFE intervention (preferred for delivery of the family therapy sessions). Prior to randomisation, the QRA or other suitably trained member of the study team will maintain contact with the nine families to remind them about the study. If families decide to withdraw at this point more families will be recruited to replace them to make up the total of at least six families.

Randomisation schedules will be produced by a member of the CTU programming team, and allocation will be determined by means of a 24-hour web-based system created by the Peninsula Clinical Trials Unit (CTU). The system will be developed in conjunction with a statistician independent from the trial team, and will use random permuted blocks with stratification by study centre.
Advantages of 2:1 allocation include:

- Increased appeal for families deciding whether to consent to randomisation
- Increased ability to test training of therapists, and ability to deliver high-fidelity treatment
- Minimal reduction in statistical power for between-groups comparisons in a full-scale evaluation
- Increased ability to recruit required number of families within an area before randomising; which will be closer to the figure needed if and when the intervention is implemented. A group of 6 families are ideally needed to deliver the intervention, but the artificial situation of a trial requires more to be recruited before randomisation – a 1:1 allocation would require 12 families, whereas the 2:1 allocation requires 9.

The CTU will inform the CI and relevant QRA and therapist(s) by means of an automated email process of which families have been allocated to the intervention group, and which to SUE. Families will be informed of their allocation by letter or email from the CTU. If allocated to the intervention arm the QRA or other unblinded member of the study team will telephone the family to arrange the SAFE post allocation assessments.

The CTU programming team will run checks before and during the trial to verify the integrity of the randomisation system.

12.3 Blinding

It is not possible to blind families to their allocated group. However, the study team (including the RA and trial mangers) will remain blind to the allocation of each family when collecting data. It is possible that participants will disclose if they have received the study intervention prior to or during such an appointment. Family therapists and the QRA will also obviously be aware of the participant's

For peer review only - http://bmjopen.bmj.com/site/about/guidelines.xhtml

allocation to trial arm, and they will be discouraged from communicating with other researchers about this.

13 TRIAL INTERVENTIONS

Families will be allocated to undertake either SUE + SAFE or SUE for a period of 16 weeks. The aim is to schedule the first SAFE session within a fortnight after allocation, but this will depend on family availability.

13.1 Post-allocation assessments (SAFE group only)

Following randomisation, a suitably qualified member of the research team, such as the QRA will contact the families allocated to SAFE, and arrange a suitable time to visit them with a study therapist. At this home visit, which is expected to take approximately two hours, the QRA will explain the intervention schedule and answer questions. The therapist will conduct the full PDI interview with each parent in preparation for the SAFE therapy sessions. The Parent Development Interview (PDI) is an audio recorded interview consisting of 20 items and taking approximately 40 minutes to complete. The full interview schedule provides background information on the relationship between the parents and the child, which will inform sessions, but also contains a measure of mentalisation (4 items) particular to the parent/child relationship, which is sensitive to contextual change (e.g. the nature of therapeutic input). The full interview will be completed prior to commencement of the SAFE sessions and the mentalisation elements will be repeated at two further points as part of session 5 of the intervention and as part of the 22 week follow-up session. If both parents are participating they should each be interviewed, but if not then a single parent should be interviewed.

The QRA will also check during this visit that families can attend the first group session, and answer any questions.

13.2 The SAFE intervention

SAFE is an intensive programme of systemic family therapy designed to treat ASD and mental health related difficulties encountered by families of CWA. Each therapy session will include two therapists. Between weeks 1 and 16, families allocated to the SAFE intervention will attend five 3 hour SAFE therapy sessions. Further details about the content of the sessions can be found in Appendix 1. Sessions 1 and 5 are multi-family sessions and will take place in a community setting with appropriate insurance for the activities undertaken. Children, including the CWA do not need to be present at these sessions. Where group sessions are not possible, for example if one family is not available on a given date, group sessions may be replaced with home visits. Sessions 2, 3 and 4 will take place in the family home. The minimum number of family members that need to be present for a home session to take place is one parent and the child with autism. Where home visits are not possible, for example where there is a resident sick relative, home visits may be replaced with sessions in a community setting. The therapists will facilitate sessions which will be video recorded, as is usual practice for therapy sessions. The videotapes (and transcribed excerpts of the footage) will not be part of the reported study data, they will only be used by the therapists in supervision sessions, in preparation for subsequent sessions, therapy process research and for training. The videotapes will be kept in a locked cupboard at the University of Plymouth by the therapist supervisor.

Appointments will be arranged at times to suit the participant families, and visit reminders will be sent out to participants as appropriate. Where families do not attend or cancel sessions, these will be rescheduled. To encourage compliance with the programme, families will be given 16 weeks to complete all therapy sessions. All sessions, whether completed or missed, will be tracked and recorded within the study database. Following completion of the therapy programme, families will

For peer review only - http://bmjopen.bmj.com/site/about/guidelines.xhtml

attend a group follow-up session at 22 weeks (±2 weeks) post-allocation. Trained support workers from local voluntary groups will attend this follow-up session and be invited to give the families information about continued support for families of CWA through existing networks.

Each session will include the following assessments will be given to families to complete:

- Client Satisfaction Questionnaire (CSQ-8) – Described in section 10
- The Helpful Aspects of Therapy Questionnaire (HAT) – Described in section 10

A Between Session Activity (BSA) homework activity will be given to families after each session. Families are given a pro-forma with key elements of the intervention as prompts. Families track strengths and difficulties in response to SAFE ideas. Families bring the completed activity to the following session. Therapists respond to the activity in the session and reflect on it in relation to the intervention process during supervision. Families are expected to spend at least 30 minutes every fortnight completing this activity.

In addition, there will be email or telephone conversation between therapists and family ("check-in time") in order to provide reassurance, collect feedback and plan home visits. At the end of each session the therapist will complete a training checklist and questionnaire (TCQ).

13.3 Support as usually employed

Families will typically attend a post-diagnosis follow-up appointment with the diagnosing paediatrician. Families of children with severe ASD and associated learning difficulties may receive support from a psychiatric nurse, including some behavioural advice. Parents of less severe children may be directed to local authority parenting classes. Classes such as 'Timeout for Autism' (Cornwall and the Isles of Scilly) and 'Understanding Autism' (Plymouth) focus on the features of ASD, instructional parenting techniques and issues associated with education. Psycho-education may also be offered, with families being directed to relevant resources for e.g. The National Autism Society, Gateway ASD, and the NHS Child and Adolescent Mental Health Services (CAMHS). For families where a member is experiencing depression or anxiety, treatment varies and is not linked to autism-related care. Initial referral is often through the GP. Patients may receive Cognitive Behavioural Therapy as part of the Improved Access for Psychological Therapies service. They may also receive medication and in extreme cases a period of in-patient hospital treatment.

14 FOLLOW-UP ASSESSMENTS

14.1 Week 24 post allocation assessments – all families

All families will be followed up at 24 weeks post-allocation (± 2 weeks) via a face-to-face visit with the RA or other suitably qualified member(s) of the research team. The visit will take place in the family's home, or at another location convenient and acceptable to both the family and the RA, or other member(s) of the research team. All study families will be asked to complete the following post-treatment assessments:

- Systemic CORE 15 (SCORE) questionnaire
- Child Behaviour Checklist (CBCL)
- Coding of Attachment-Related Parenting for use with children with Autism (CARP-A)
- Caregiving Helplessness Questionnaire (CGHQ)
- Reflective Functioning Questionnaire (RFQ)
- Patient Health Questionnaire – Somatic Anxiety Depressive Symptoms (PHQ-SADS)
- EQ-5D-5L
- Child Health Utility 9 Dimensions (CHU-9D)

For peer review only - http://bmjopen.bmj.com/site/about/guidelines.xhtml

- Resource Use Questionnaire

The SCORE, CARP-A and CGHQ measures should be completed at the visit, and returned by the RAs to the Clinical Trials Unit (CTU) according to an agreed procedure. Participants may ask to complete the remaining week 24 assessments in their own time, and return them in a prepaid envelope to CTU. If they decide to do this families will be given a contact number to call should they have need help with questionnaire completion, and advised to return them within the next 14 days. A reminder letter will be sent to participants if the questionnaires are not returned within the 14 day period.

15 END OF THE STUDY

The end date for the study is the date that last family completes the qualitative feedback.

16 QUALITATIVE COMPONENT

The qualitative component will investigate four key aspects of the study experience, including; families' experiences of the study (including intervention), therapists' experiences of the intervention, reasons for eligible families declining, and reasons for families withdrawing from the study, as follows:

- Investigate participants' responses to candidate clinical outcomes, and explore acceptable participant reported outcomes in relation to the SAFE intervention.
- Explore participants' experience of and adherence to the SAFE intervention.
- Explore participants' experience of Support as Usually Employed (SUE), including any support and interventions they may have received
- Explore therapists' experiences of taking part in the study, including training and implementation of the intervention and acceptability of a future RCT.
- Identify reasons why eligible patients decline to take part in the study and reasons for early withdrawal from the study.
- Families' experience of the SAFE intervention
- The perceived benefits and challenges of using SAFE techniques
- The families' responses to SAFE sessions, materials and specific activities
- The use of SAFE techniques at home, between sessions

The qualitative data collection will attend to the objectives of the study and will include focus group sessions that will be audio-recorded and transcribed verbatim. Participants will be able to review the transcript. The families that received SAFE + SUE and the families that received SUE will attend separate focus groups to avoid discussion of topics such as the SAFE intervention, which is not relevant to the experience of all participants. Participants will be given a qualitative study information sheet at the week 24 home visit to consider before agreeing to take part. Consent/assent will be taken for the qualitative study by a suitable qualified member of the research team either at the week 24 home visit, or on the day of qualitative study.

After the week 24 post allocation assessments have been completed, participating families that have consented to participate in the qualitative focus groups will be given details of the time and location for the family feedback day by the QRA. The family feedback day will involve several separate focus group sessions organised to take place over the period of a morning or an afternoon at a local venue for each centre, for ease of access to participants. If participants are keen to provide feedback, but are unable to attend the family feedback day, a separate interview may be arranged with the QRA. Parents in each family will attend a general focus group relating to the study as well as a specific focus group relating to their experience of SAFE + SUE or SUE only. While the parents are engaged

For peer review only - http://bmjopen.bmj.com/site/about/guidelines.xhtml

in the two focus groups the children will also be asked to participate in two separate focus groups: to explore their experiences of the study and of SAFE + SUE or SUE. The focus groups will require input from two QRAs and two members of the SAFE research team. The Focus Group Discussion (FGD) topic guides will include questions about recruitment, study outcome measures, experiences of the SAFE intervention, and additional comments to help improve the study design for the main study. The focus groups will take place at a local venue for each centre, for ease of access to participants.

16.1 Family focus group – experience of study participation (all families)

The QRA at each centre will invite all families (SAFE + SUE and SUE) from each cohort to attend focus groups to discuss their experience of participating in the study. There will be separate focus groups for the SAFE + SUE and SUE groups. The groups will be for the parents and separate concurrent focus groups will be conducted for the children (section 15.4). These focus groups will include topics that are relevant to all families, such as the participant identification process and the acceptability of randomisation and outcome measures.

16.2 Family focus group – experience of SAFE (intervention only)

The QRA at each centre will invite the parents in all of the six families that have participated in the SAFE intervention + SUE from each cohort to participate in focus groups investigating their experience of the intervention, the perceived efficacy of the SAFE intervention and its applicability. They will also be asked to describe any support they had received outside SAFE and how it complemented SAFE or otherwise.

16.3 Family focus group – experience of SUE

Following the discussion of their general experience of participating in the study the families who received SUE will be invited by the QRA to discuss their experience of support as usual over the course of the trial. This will include an exploration of what support they have received within and outside the NHS, for example any input regarding their child, advise, information, educational support

16.4 Child focus group – experience of the study and SAFE and SUE

The children's experience of SUE and SAFE is extremely important and focus groups will be conducted in two separate groups to try to capture children's experience. This will include an exploration of their experience of the child-focused activities employed in the study. Following this they will be asked to consider their experience of SUE or SAFE + SUE in terms of the people that were involved with them, including professional and therapists and any support that they received. For the SAFE + SUE group there will be specific questions regarding their experience of aspects of the intervention. These child focus groups will involve visual/drawing as well as verbal discussion of their experiences.

16.5 Therapist focus group

About 1 to 2 weeks after completion of each of their cohorts the RA will invite all four of the study therapists to attend a focus group interview session to discuss their overall experience of taking part in the study, carrying out the SAFE sessions and the training and supervision. Therapists will be given an information sheet and consent will be taken by a suitably qualified member of the research team, before therapists are interviewed. The RA will lead the focus group process, which will take place either in an appropriate venue.

16.6 Interview - declined (all families)

If an eligible family declines to participate in the study they will be invited at the point of decline to engage in a short focussed telephone interview with a member of the study team to discuss their decision not to participate.

For peer review only - http://bmjopen.bmj.com/site/about/guidelines.xhtml

16.7 Interview - withdrawn (all families)

If a family withdraws from the study, a member of the study team will invite the family at the point of withdrawal to engage in a short focussed telephone interview to discuss the reasons for their decisions, and how the main trial can be designed to address these issues.

16.8 Qualitative analysis

The qualitative analysis will focus on families' experiences of the study (SAFE + SUE or SUE), therapists' experiences of the intervention, reasons for eligible families declining, and reasons for families withdrawing from the study. There are two elements to the qualitative analysis: i) to investigate both SAFE + SUE and SUE group participants' experience of study procedures in order to inform the design of the main trial, ii) to explore the SAFE + SUE group's experiences of intervention and of the SUE group's experience of any support and interventions that they may have received during the course of the study. This is in order to gain information about what specific aspects of the interventions are perceived to be more or less helpful. In addition the qualitative analysis will separately examine the experience of the adults and the children in the families. This is to ensure that the experience of the children are fully explored and that this is recognised as a priority. Ultimately, the aim of analysis is to understand families' experience in order to refine the intervention and prepare for future funding.

There are different varieties of qualitative analyses, but they share a number of important perspectives: an interest in focussing in detail on the nature of people's experience of therapy. This includes their understanding, but also the emotional impact of the intervention. There is recognition that people's experience is idiosyncratic and unique and that researcher's engage in an interpretative process in trying to understand their experience[42-45]. The analysis, therefore, involves a reflexive component in that the researcher is required to attend to their own assumptions and experiences, which may colour their interpretation of the data. As an example parents of a child with autism frequently report experiences of shame, humiliation and anger in people's misperceptions of them as inadequate parents. The researcher's own experiences as a parent or being parented will inevitably influence their analysis. A structure of personal reflection in the analytical process and supervision throughout analysis is essential and this will be facilitated by the researchers engaging in 'bracketing interviews', where they engage with a research supervisor in an in-depth reflective interview [47].

Alongside an emphasis on the unique features of family's experience the form of qualitative analysis employed in this study draws on social constructionism in recognising that wider culturally shared understandings or discourses, such as definitions of autism, 'normal' parenting, 'normal' child development and family roles shape their experiences and actions. Hence the qualitative analysis will explore which discourses are salient in family's accounts of the impacts of SAFE and in turn the specific understandings they have of these discourses, for example explanations of the causes and prognosis of autism.

In addition, the qualitative analysis employed recognises that SAFE will provoke thoughts and feelings in families which may at time be emotionally challenging for them. Hence the analysis will also focus on aspects of the experience that are more implicit or defended for the families. The earlier example of a sense of shame and anger that many parents have experienced may be expressed more indirectly, for example, more by their tone of voice, hesitation and expressions than explicit statements.

Focus group interviews will be audio recorded and transcribed verbatim. Data will be managed using proprietary computer assisted qualitative data analysis software, for example, Nvivo 10, and analysed thematically. Rigour of analysis will include 'respondent validation', whereby participants

For peer review only - http://bmjopen.bmj.com/site/about/guidelines.xhtml

are provided with a copy of their transcript and analysis so that they can assess whether the interpretations being made about the data, accurately represent them. In addition, a second qualitative researcher will conduct an independent analysis of a subset of half of the focus group transcripts. Researchers will then meet to discuss and agree the findings, which will then be presented to the Family Consultation Group for discussion. Any significant differences of opinion will be discussed with the Chief Investigator.

17 ECONOMIC EVALUATION

This feasibility study will enable the development of a framework for subsequent cost-effectiveness analysis alongside a future RCT. In particular, economic evaluation methods will be developed and tested regarding the collection of resource use, costs and outcome data (EQ-5D-5L & CHU-9D). The objectives of the economic work will be to:

- Identify the resource use associated with delivering SAFE to determine the cost per family/per therapy hour.
- Use the Resource Use Questionnaire for this population to identify the NHS and Social Care resource use. Any missing data in the Resource Use Questionnaire will be recorded. To check if any resource use categories are misunderstood, Resource Use Questionnaire completion will be matched with medical records for a subgroup of families, which will help to develop strategies to minimise missing data in the future definitive trial.
- Assess the feasibility of family self-completing the EQ-5D-5L and the CHU-9D in order to estimate Quality Adjusted Life Years (QALYs). These steps will facilitate the full cost-effectiveness analysis in a definitive randomised controlled trial.

18 SAFETY REPORTING

The risks associated with participating in this study are considered minimal, with no adverse events anticipated in any participant. For those in the intervention group, there is a slight chance that the SAFE family therapy sessions could lead to an initial increase in family disagreements as family members learn how to change the way they solve problems and talk with one another. However, the purpose of the intervention is ultimately to equip families with skills to handle these difficulties by learning how to change the way they solve problems and talk with one another, and the SAFE family therapists will be available to provide support and will be trained to handle any emerging problems. Should any issues arise the SAFE family therapists will have access to a consultant clinical psychologist to provide further support and advice. There are no requirements to report non-serious adverse events for this study, only serious adverse events relating to mental health will be reported in this study, for each participant, and these will be monitored as below.

18.1 Serious Adverse Events: definition and identification

A serious adverse event (SAE) is defined as an untoward occurrence that:
(a) results in death;
(b) is life-threatening;
(c) requires hospitalisation or prolongation of existing hospitalisation;
(d) results in persistent or significant disability or incapacity;
(e) consists of a congenital anomaly or birth defect; or
(f) is otherwise considered medically significant by the investigator.

SAEs may be volunteered by the participant or discovered by the therapists, QRAs, or other member of the research team during the SAFE family therapy sessions, or as a result of direct reporting (e.g.

For peer review only - http://bmjopen.bmj.com/site/about/guidelines.xhtml

by telephone) by a family member, independent clinician or other informant. In addition, at the 24 week follow-up face-to-face meeting, the RA or other qualified member of the research team will prompt the family to report any serious mental health issues satisfying the SAE criteria (in the CWA or any participating family member). Serious adverse events should be recorded from the time of consent until the date the participant completes the 24 week follow-up or withdraws from the study. Any such SAE, whether thought to be related to the trial or not, must be reported to the CTU on the study specific SAE form (regardless of whether all relevant information is available) by the local site lead or another member of the research team by fax (01752 315254), or email (penctudata@plymouth.ac.uk) within 24 hours of the research team becoming aware of it.

18.2 Serious Adverse Events: reporting

Completion of the SAE form must include the CI's assessment of causality i.e. whether there is a reasonable causal relationship between the SAE and the trial. If incomplete information is available at the time of reporting, all appropriate information relating to the SAE should be forwarded to the CTU as soon as possible. Completed SAE forms with CI signature and assessment of relatedness (if relevant) should be sent to the CTU within seven days.

If the CI considers that the SAE is not, or is unlikely to be, related to the trial, the CTU will obtain a second assessment of causality from the independent assessor. Since no adverse events are expected, any SAE which in the opinion of either adjudicator is possibly related to the trial will be reported by the CTU to the Research Ethics Committee within 15 days of the local research team having become aware of the event, using the SAE report form for research other than clinical trials of investigational medicinal products (non-CTIMPs), published on the Health Research Authority (HRA) website. The report will be copied to the Sponsor and TSC.

All SAEs will be followed until either stabilised if chronic conditions or resolved. The CTU will routinely notify the CI by email of all reported SAEs as they occur and will report all SAEs to the TSC and Sponsor on a quarterly basis. The CTU will be responsible for the preparation and submission of an annual safety report to the REC.

19 PARTICIPANT WITHDRAWAL

A family may withdraw from the intervention or the study itself at any time without giving a reason, and without it affecting the child's clinical care. Families will be asked to give a reason for withdrawal from the intervention or study but do not have to provide one. Families who wish to withdraw from the SAFE intervention sessions will be invited to continue with follow-up as usual. Those who withdraw completely from study intervention or follow-up will not be replaced. Participants that withdraw should be reported to CTU by completing the withdrawal CRF. Participants that withdraw should also be invited to participate in the qualitative interview (described in section 15.7 above). The CTU data management team will ensure that participants who formally withdraw from the study are not contacted for any subsequent follow-up data collection (aside from any partial follow-up arrangements made with individual families). Data collected prior to withdrawal will be included in the study analysis unless a family specifically requests that their data are removed from the database.

20 DATA MANAGEMENT

20.1 Participant numbering

Each family participating in the study will be allocated a unique study number via the study management website following registration of key details (section 12.1) which will subsequently be recorded on all baseline forms prior to return to CTU. Relevant members of the study team will be

For peer review only - http://bmjopen.bmj.com/site/about/guidelines.xhtml

notified of this number by CTU via email, when the family is randomised into the study. This study number, and the initials of the child with ASD will thereafter identify families in all study-related documentation. A record of names, addresses, telephone numbers and email addresses of relevant family members, linked to the family study number will be stored securely on the study database at CTU for administrative purposes.

20.2 Data collection

Researcher-collected data will be recorded on paper-based study-specific case report forms (CRFs). All persons authorised to collect and record study data will be listed on the study site delegation log. Parents will complete self-reported outcome measures on paper-based forms during face-to-face meetings with a member of the research team at baseline and 24 weeks. The researchers will return these completed questionnaires and completed CRFs to the CTU.

20.3 Data entry

Completed paper CRFs will be checked and signed by the relevant RA before being sent to the CTU. Original CRF pages will be posted to the CTU at agreed time points for double-data entry on to a password-protected database, with copies retained at the study site. Forms will be tracked using a web-based trial management system. Double-entered data will be compared for discrepancies using a stored procedure. Discrepant data will be verified using the original paper data sheets.

20.4 Source data

For the purposes of this study, source data will include the NHS hospital records of the child with ASD as well as parent-completed self-report outcome measures. All data not routinely captured in the hospital notes but recorded straight into the CRF will also be classified as source data. Source data will be made available for the purposes of on-site monitoring visits, audits, and regulatory inspections.

20.5 Case Report Form and questionnaire completion

The site will be provided with study-specific CRFs and questionnaires for completion by family members. CRF data must be recorded by the study research team member or designee, as accurately and completely as possible. They are responsible for the timing, completeness, legibility, accuracy and signing of the CRFs. Pre-paid envelopes will be provided as required for return of completed CRFs and questionnaires to the CTU.

20.6 Video and audio recorded data

Video data will be collected at baseline and 24 weeks for all study participants, and at each intervention visit. Audio data will be collected at the focus groups and therapist interviews. These data will be transferred to a password protected University of Plymouth computer of the research team (not PenCTU) immediately upon return from the visit. Video and audio data will not be left stored on recording devices. Audio data will be transcribed, anonymised and deleted as soon as is practicable. Those participants who have not given their consent for their video data to be used for subsequent supervision and training purposes, will have their video files deleted at the end of the study.

20.7 Data confidentiality

The research team will ensure that participants' pseudo-anonymity is maintained on all documents. Data will be collected and stored in accordance with the current legal and regulatory documentation.

Electronic study records will be stored in a SQL server database, stored on a restricted access, secure server maintained by the University of Plymouth. Data will be entered into the database via a bespoke web-based data entry system encrypted using SSL. Access to electronic data will be

For peer review only - http://bmjopen.bmj.com/site/about/guidelines.xhtml

permission based, and at the discretion of the CTU data management team. Data entered onto the database will be backed up according to PenCTU SOPs.

Within the CTU, anonymised paper-based study data will be stored in locked filing cabinets within a locked office. Copies of study data retained at the lead study site will be securely stored for the duration of the study prior to archiving.

20.8 Access to data

The CTU data team will have access to study data, including identifiable data. Other members of the study team and the CTU will have restricted access to pseudo-anonymised study data. Access will be granted to the Sponsor and host institution on request, to permit study-related monitoring, audits and inspections. Access to the database will be overseen by the CTU data manager and trial manager.

20.9 Archiving

Following completion of data analysis and submission of the end of study report, the Sponsor will be responsible for archiving the study data and essential documentation in a secure location for a period of five years after the end of the trial. No trial-related records should be destroyed unless or until the Sponsor gives authorisation to do so.

21 STATISTICAL CONSIDERATIONS

21.1 Sample size

In this feasibility trial no formal statistical testing of between group differences is planned. Sample size has been selected heuristically with the goal of i) demonstrating that participants can be recruited at a rate sufficient to run a full scale evaluation of SAFE at a later date, ii) demonstrating that it is possible to train therapists and deliver SAFE to patients within the study treatment settings, and iii) demonstrate that the data collection procedures are effective, and that the data collection is acceptable to families, and not overly burdensome.

21.2 Statistical analysis

A detailed statistical analysis plan will be developed, and approved by the Trial Steering Committee, prior to final database lock and analyses.

Data will be analysed and presented as is appropriate for a feasibility study, in particular concentrating on descriptive analyses and undertaking no formal comparisons between groups. Reporting will follow the principles of the CONSORT Statement (http://www.consort-statement.org), using the checklist and flowchart as recommended in the CONSORT extension for Randomized Pilot and Feasibility Trials (http://www.consort-statement.org/extensions/overview/pilotandfeasibility.) [48].

The flowchart will provide detail about the number of families approached, number eligible, number consenting, number randomised, number receiving allocated intervention and number assessed for outcome data at each time point. As appropriate, details will be given for individual members of the family, for example, how many family members there are and how many completed each questionnaire. Wherever possible, detailed reasons will be given for exclusions, loss to follow-up, non-completion of outcome measures etc.

Numbers will also be provided by centre and group, to inform the logistics of recruiting nine families prior to randomisation and following them up after randomisation.

For peer review only - http://bmjopen.bmj.com/site/about/guidelines.xhtml

For those randomised to the SAFE intervention, adherence will be reported according to the number of group sessions attended and participation of individual family members at each of the therapy sessions.

Completeness of data will be reported for each outcome measure at each relevant time point. Again this will be reported for individual family members as appropriate.

For each outcome measure, the relevant scores will be calculated and presented descriptively by trial arm. Where available, published guidelines will be used to process, score and summarise the measures including, for example, the use of imputation in the event of missing items on a questionnaire. Summary measures will be calculated as appropriate – e.g. means and standard deviations, medians and ranges, numbers and percentages in categories. These measures will be presented both for baseline and for the final follow-up.

In a future definitive trial, covariates may be included in comparative analyses, in particular the baseline values of any particular outcome, plus any prognostic variables used to stratify randomisation (although none are expected beyond centre). In addition, clustering according to any group effect and/or therapist effect will need to be taken into account. The small numbers involved in this feasibility study preclude any sensible assessment of the size of these random effects, which will need to be estimated from suitable literature in planning that future trial.

The only analysis contrasting the two groups will be an interval estimate in the form of a 95% confidence interval for the primary outcome, so that the plausibility for the effect size used in the sample size calculation for the full trial can be assessed. For this purpose the baseline values will be used in an Analysis of Covariance, with acknowledgment that no effects are included for group or therapist.

22 DATA MONITORING

22.1 Data monitoring plan

The CTU trial manager and data manager will devise a risk-based monitoring plan specific to the study which will include both central monitoring strategies and study site visits (usually by the trial manager) as appropriate. The monitoring plan will be agreed by the Sponsor and reviewed periodically in line with updated risk assessments. The risk assessment and monitoring plan are active documents and will remain subject to change throughout the study.

Data will be monitored centrally for quality and completeness by the CTU and every effort will be made to recover data from incomplete pages. The CTU data manager will oversee data entry and initiate processes to resolve data queries where necessary.

All study procedures will be conducted in compliance with the protocol and according to the principles of Good Clinical Practice. Procedures specifically undertaken by the CTU team (e.g. data management, trial management and study monitoring) will be conducted in accordance with CTU SOPs. The participating centres will be required to permit the CTU trial manager or deputy to undertake study-related monitoring to ensure compliance with the approved study protocol and applicable SOPs, providing direct access to source data and documents as requested.

For peer review only - http://bmjopen.bmj.com/site/about/guidelines.xhtml
For peer review only

22.2 Quality assurance

The CI will be responsible for the overall running of the trial. The CTU will coordinate trial-related activities and assist with overall trial management, monitoring and production of progress reports. The CTU will also organise the web-based randomisation, prepare the database, provide double data entry into the database, and oversee safety-reporting activities.

Prior to activating a centre to recruitment, it is necessary for all staff members working on the trial to participate in an induction session. This will be carried out via teleconference or by a site initiation visit. An accreditation checklist will be completed for centres to confirm that pre-activation activities have been completed and all relevant staff members are able to participate. Support will be offered to staff at participating centres to ensure they remain fully aware of trial procedures and requirements. Additional support and training will be offered to centres as appropriate where necessary (e.g. if the recruitment rate is lower than expected).

A Trial Master File will be set up and held securely at the CTU, in accordance with CTU SOPs. CTU will produce and provide the lead site with a Site File. Any updates to essential trial documentation will be circulated to the lead site – it is the responsibility of the site to update the Site File as necessary.

22.3 Trial Management Group (TMG)

The TMG includes a multidisciplinary team of clinicians and researchers who have considerable expertise in all aspects of trial design, conduct, analysis and quality assurance. A PPI representative will also be an active member of the TMG.

22.4 Trial Steering Committee (TSC)

The TSC will be composed of a majority of independent members and provide advice, through its independent Chair, to the trial funder, the trial sponsor, the Chief Investigator, and the host institution, on all appropriate aspects of the project. The TSC will assess the progress of the trial, adherence to the protocol, and the consideration of new information of relevance to the research question. The rights, safety and well-being of the participants are the most important considerations, and should prevail over the interests of science and society. The TSC will ensure appropriate ethical and other approvals are obtained in line with the project plan, and will agree proposals for substantial protocol amendments and provide advice to the sponsor and funder regarding approvals of such amendments. The TSC will provide advice on all aspects of the trial/project.

22.5 Patient and Public Involvement (PPI) – Family Consultation Group

Families of CWA initiated the development of this study by communicating their complex needs and dissatisfaction with current service provision through the Plymouth Autism Network. This Network was set up by the CI in 2011 to bring clinicians, carers, academics and individuals with ASD together to share ideas, research findings and experiences. The challenges facing families of CWA were explored by conducting in-depth interviews and surveying over 90 families regarding their needs and the treatment they received post-diagnosis. Less than 9% of families agreed that current treatment helped with the problems they face. The survey revealed a strong need for interventions which support the whole family. The following comments articulate what families need:

- "Counselling for ALL family members. Help with individual family problems, pressures and strains."
- "They should help improve family life"
- "Help and support for parents coping with sleep deprivation, violence, social ostracism as

For peer review only - http://bmjopen.bmj.com/site/about/guidelines.xhtml

well as working and running a home"

- "There needs to be a comprehensive support service for children and their families."
- "A lot more support to the whole family"
- "The most important thing for any child with ASD is that they are happy within themselves and the family environment"
- "For parents to understand their child properly"

This pilot data led to the development of a research team within the Welcome Lab at the University of Plymouth, which included a Family Consultation Group. The Family Consultation Group worked with the CI, and colleagues to develop and refine the SAFE intervention prior to the current study. These families have also contributed to the current study by working with the SAFE study team to create a recruitment and treatment plan, which will be manageable for families. They have offered advice about how it is best to communicate with families at the start of the study and as it progresses.

The Family Consultation Group are essential members of the team and will work as an advisory group throughout the feasibility study and beyond. They will attend research meetings and present findings at local and national conferences. The Family Consultation Group are viewed as experts in their own lives and the lives of families with similar challenges. For this reason, the SAFE study team will continue to work with them in a supportive manner as collaborators. Their contribution is valuable in the same way as other experts on the team, and we aim to facilitate one another. As stated above, the Family Consultation Group have been active in contributing to the research plan. Their input is of particular value in developing recruitment procedures, designing participant information packs and providing information about potential barriers to retention. The Family Consultation Group have been involved in the preparation and delivery mechanisms for the training programme for research staff and therapists. With their help we aim to ensure that recruited staff work in a sensitive and informed manner with participants. We also value their input in interpreting and reporting data; in particular commenting on possible ways to overcome challenges for the main RCT. In the long-term we will develop other Family Consultation Groups in additional centres for the proposed future Multi-Centre RCT, these families can help by identifying local networks and share their experience with new groups. The current Family Consultation Group are proactive campaigners for change and have extensive knowledge of existing bodies such as the National Autistic Society. They can also provide a family-centred perspective on research outcomes. They are, therefore, well placed to collaborate with the SAFE study team in planning next steps and disseminating findings at local and national levels. Family involvement benefits the research in numerous ways. Involvement has already helped to ensure that the intervention is relevant and accessible for families. The Family Consultation Group has played a vital role in planning the proposed study: in developing acceptable, sensitive and appropriate procedures, materials and training programmes. This project was initiated by, and is supported and informed by their input.

23 ETHICS APPROVAL

The study will be undertaken subject to appropriate Research Ethics Committee (REC) approval and Health Research Authority (HRA) approvals. The trial will be conducted in accordance with the protocol, the principles of the Declaration of Helsinki and ICH GCP. Any amendments of the protocol will be submitted to the Sponsor, HRA and REC for approval.

Substantial amendments that require review by REC will not be implemented until the REC and HRA grant a favourable opinion. All correspondence with the REC and HRA will be retained in the Trial Master File and Site Files. An annual progress report will be submitted to the REC within 30 days of

For peer review only - http://bmjopen.bmj.com/site/about/guidelines.xhtml

the anniversary date on which the original favourable opinion was given, and annually until the trial is declared ended. If the study is ended prematurely, the Chief Investigator will notify the REC, including the reasons for the premature termination. Within one year after the end of the study, the Chief Investigator will submit a final report with the results, including any publications/abstracts, to the REC.

23.1 Protocol compliance

Protocol deviations will be monitored by the CTU and reported to the Chief Investigator and Sponsor as appropriate. Significant deviations from the protocol, which frequently recur are not acceptable and may potentially be classified as a "serious breach".

23.2 Notification of serious breaches of GCP and/or the protocol

A "serious breach" is a breach of the protocol or of the conditions or principles of Good Clinical Practice, which is likely to effect to a significant degree –

- The safety or physical or mental integrity of the subjects of the trial; or
- The scientific value of the trial

The Sponsor will be notified immediately of any case where the above definition applies during the trial period. The Sponsor is responsible for notifying the REC of a serious breach in any study within seven days of the matter coming to their attention.

24 STATEMENTS OF INDEMNITY

This is an NHS-sponsored research trial. If an individual suffers negligent harm as a result of participating in the trial, NHS indemnity covers NHS staff and those people responsible for conducting the trial who have honorary contracts with the relevant NHS Trust. In the case of non-negligent harm, the NHS is unable to agree in advance to pay compensation, but an ex-gratia payment may be considered in the event of a claim. Any harm to participants arising from the design or management of the research is covered by the NHS Litigation Authority. There are no arrangements for the Sponsor to pay compensation in the event of harm to research participants where no legal liability arises.

25 PROGRESSION TO A FULL TRIAL

The decision as to whether to proceed with the development of a future large-scale RCT following this feasibility study will be based on a number of criteria. These include the number of families recruited, participants' willingness to undergo random allocation to the trial intervention or control group, the proportion of participants retained in the study, the extent of families' engagement in the trial intervention, and the acceptability of the intervention. At the end of the study, these factors will be used to decide the case for progressing to a substantive RCT, in conjunction with the TSC.

26 DISSEMINATION POLICY

If the feasibility study demonstrates successful recruitment and an ability to deliver the intervention an important part the dissemination plan is to raise awareness of the need for a larger multi-centre trial. Targeted summaries of the findings and presentations will be disseminated to policy makers. The findings will also be broadly disseminated, but in a manner appropriate to a feasibility study. National conference presentations and published papers will be prepared to inform clinicians, academics and therapists about our feasibility results and generate interest in the future trial. Existing connections including the Association for Family Therapy, the National Autistic Society and the Institute of Family Therapy will be utilised to reach relevant audiences. The qualitative findings will also be published with detailed accounts of the families' reactions to SAFE and their views on its

For peer review only - http://bmjopen.bmj.com/site/about/guidelines.xhtml

usefulness. A summary of study results in plain English will be available on the Peninsula Clinical Trials website: www.plymouth.ac.uk/penctu

27 FINANCE

The SAFE study is being funded by an NIHR RfPB grant (reference number PB-PG-0815-20058), and the Autistica charity (reference number 7239). Finances will be managed by University Hospitals Plymouth NHS Trust.

28 REFERENCES

1. American Psychiatric Association (2013) Diagnostic and Statistical Manual of Mental Disorders, 5th edn. American Psychiatric Association, Washington, DC.
2. Sasson, N. J., Lam, K. S. L., Parlier, M., Daniels, J. L. and Piven, J. (2013) Autism and the broad autism phenotype: familial patterns and intergenerational transmission. Journal of Neurodevelopmental Disorders, 5, 11-18.
3. Dykens, E. M., Fisher, M. H., Taylor, J. L., Lambert, W. and Miodrag, N. (2014). Reducing Distress in Mothers of Children With Autism and Other Disabilities: A Randomized Trial. Pediatrics, 2013- 3164.
4. Hallett , V., Ronald, A., Colvert, E., Ames, C., Woodhouse, E., Lietz, S., … Happe, F. (2013) Exploring anxiety symptoms in a large-scale twin study of children with autism spectrum disorders, their co-twins and controls. Journal of Child Psychology and Psychiatry, 54, 1176– 1185.
5. Brereton, A.V., Tonge, B. J. and Einfeld, S. L. (2006). Psychopathology in children and adolescents with autism compared to young people with intellectual disability. Journal of Autism and Developmental Disorders, 36, 863-870.
6. Kim, J. A., Szatmari, P., Bryson, S. E., Streiner, D. L. and Wilson, F. J. (2000).The prevalence of anxiety and mood problems among children with autism and Asperger syndrome. Autism, 4, 117- 132.
7. Howlin, P. (1997) Interventions for people with autism: recent advances. Advances in Psychiatric Treatment, 3, 94-102.
8. Mazefsky, C. A., Folstein, S. E. and Lainhart, J. E. (2008). Overrepresentation of Mood and Anxiety Disorders in Adults with Autism and Their First Degree Relatives: What Does it Mean? Autism Res ,1, 193-197.
9. Daniels, J. L., Forssen, U., Hultman, M., Cnattingius, S.,Savitz, D. A., Feychting, M and Spraren, P. (2008). Parental Psychiatric Disorders Associated With Autism Spectrum Disorders in the Offspring. Pediatrics, 121, 1357-1162.
10. Siklos, S. and Kerns, K. A. (2008). Assessing Need for Social Support in Parents of Children with Autism and Down Syndrome. J Autism Dev Disord, 36, 921-933.
11. McCrone, P., Dhanasiri, S., Patel, A., Knapp, M. and Lawton-Smith, S. (2008) The cost of mental health care in England to 2026. Executive Summary. London: The King's Fund.
12. Buescher, A. V. S., Cidav, Z., Knapp, M. and Mandell, D. S. (2014) Costs of Autism Spectrum Disorders in the United Kingdom and the United States. JAMA Pediatrics, e1-e8.
13. Osborne, L. A., McHugh, L., Saunders, J. and Reed, P. (2008). Parenting Stress Reduces the Effectiveness of Early Teaching Interventions for Autistic Spectrum Disorders. Journal of Autism and Developmental Disorders, 38, 1092-1103.
14. Pisula, E. (2011). Parenting Stress in Mothers and Fathers of Children with Autism Spectrum Disorders, A Comprehensive Book on Autism Spectrum Disorders, Dr. Mohammad-Reza Mohammadi (Ed.), Available from: http://www.intechopen.com/books/a-comprehensive-

For peer review only - http://bmjopen.bmj.com/site/about/guidelines.xhtml

bookon- autism-spectrum-disorders/parenting-stress-in-mothers-and-fathers-of-children-with-autismspectrum-disorders
15. Bolton, P. F., Pickles, A., Murphy, M. and Rutter, M. (1998). Autism, affective and other psychiatric RfPB Competition 28 Stage 2 - South West Reference: PB-PG-0815-20058 Date submitted: 30 March 2016 disorders: patterns of familial aggregation. Psychological Medicine, 28, 385-395.
16. Demir, T., Dogangun, B., Karacetin, G., Mukaddes, M. N., Demir, D. and Copur, M. (2008). Psychiatric Disorders and Symptoms in Mothers of Children with Autistic Disorder. Neurology, Psychiatry and Brain Research, 15, 1-5.
17. Piven, J. and Palmer, P. (1999). Psychiatric disorder and the broad autism phenotype: evidence from a family study of multiple-incidence autism families. Am J Psychiatry, 156, 557-563.
18. Nikmat, A. W., Ahmad, M., Oon, N. L. and Razali, S. (2008). Stress and psychological wellbeing among parents of children with autism spectrum disorder. ASEAN Journal of Psychiatry. 9,(2) 65-72.
19. McKenzie, R. and Dallos, R. (2015) Autism and Family Dynamics. Manuscript in preparation.
20. Crittenden, P., Dallos, R., Kowloska, K., and Landini, A. (2014). Attachment & Family Therapy. Maidenhead: Open University Press/McGraw-Hill.
21. Bieberich, A. and Morgan, S. B. (1998). Brief report: affective expression in children with autism or Down's syndrome. Journal of Autism and Developmental Disorders, 28, 333-338.
22. Hutman, T., Siller, M. and Sigman, M. (2009). Mothers' narratives regarding their child with autism predict maternal synchronous behavior during play. Journal of Child Psychology and Psychiatry, 50, 1255-1263.
23. Konstantareas, M. K. (1991). Autistic, learning disabled and delayed children's impact on their parents. Canadian Journal of Behavioural Science, 23(3), 358-375.
24. Murphy, M., Bolton, P. F., Pickles, A., Fombonne, E., Piven, J., Rutter, M. (2000). Personality traits of the relatives of autistic probands. Psychol Med. 30,1411-1424.
25. Blackledge, J. T., and Hayes, S. C. (2006). Using Acceptance and Commitment Training in the Support of Parents of Children Diagnosed with Autism. Child & Family Behavior Therapy, 28(1), 1-18.
26. Davis, G. Lead Paediatric Psychologist, Plymouth Hospitals Trust. Personal communication, September 10, 2014.
27. NICE guidelines [CG170] https://www.nice.org.uk/guidance/cg170/chapter/patient-centred-care
28. NICE guidelines [CG128] https://www.nice.org.uk/guidance/cg128
29. Munroe, E. (2011) The Munroe Review of Child protection: Final report, Dept. of Education, HM Stationary Office, UK.
30. Goodman, S. and Trowler, I. (2012) Social work re-claimed: Innovative frameworks for child and social work Practice. London: Jessica Kingsley
31. NHS England (2015). Future in Mind: promoting, protecting and improving our children and young people's mental health and wellbeing. Dept of Health Publication Gateway Ref. No 02939.
32. IAPT | CYP IAPT Children and Young People's Project. (n.d.). Retrieved January 5, 2015, from http://www.iapt.nhs.uk/cyp-iapt/
33. Bromley, J., Hare, D. J., Davison, K. and Emerson, E. (2004). Mothers supporting children with autistic spectrum disorders: Social support, mental health status and satisfaction with services. Autism, 8, 409-423.
34. Stratton, P (2005). Report On The Evidence Base Of Systemic Family Therapy. Association for Family Therapy. RfPB Competition 28 Stage 2 - South West Reference: PB-PG-0815-20058 Date submitted: 30 March 2016

For peer review only - http://bmjopen.bmj.com/site/about/guidelines.xhtml

35. Dallos, R. and Draper, R. (2015 4th Edn) An Introduction to Family Therapy. Maidenhead: Open Univ. Press/McGraw Hill.
36. Monteiro, M. (2016). Family Therapy and the Autism Spectrum: Autism Conversations in Narrative Practice. London: Routledge.
37. Estrada, A. U., and Pinsof, W. F. (1995). The effectiveness of family therapies for selected behavioural disorders of childhood. Journal of Marital and Family Therapy, 21(4), 403-424.
38. Pellicano, E., Dinsmore, A. and Charman, T. (2014). What should autism research focus upon? Community views and priorities from the United Kingdom. Autism, Special Issue Article, 1-15.
39. Wallace, S., Parr, J., & Hardy, A. (2013). One in a hundred: Putting families at the heart of autism research. London: Autistica.
40. Stratton, P., Bland, J., Janes, E. and Lask, J. (2010). Developing an indicator of family function and a practicable outcome measure for systemic family and couple therapy: the SCORE. Journal of Family Therapy, 32, 232-258.
41. SHIFT (2015) Self-Harm Intervention, Family Therapy: a randomised controlled trial of family therapy vs. treatment as usual for young people seen after second or subsequent episodes of self harm. Protocol Grant Number: 470772 HTA Reference Number: 07/33/01.
42. Spitzer, R. L., Kroenke K, Williams, J. B. W. (1999) for the Patient Health Questionnaire Primary Care Study Group. Validation and utility of a self-report version of PRIME-MD: the PHQ Primary Care Study. JAMA, 282, 1737-1744.
43. Blazey, L.(2007). Coding of Attachment-Related Parenting for use with children with Autism (CARP-A). Manchester University.
44. Moss, E., Bureau, J., Cyr, C., Mongeau, C. and St-Laurent, D. (2004). Correlates of attachment at age 3 Construct validity of the Preschool Attachment Classification System. Development Psychology, 40, 323-334.
45. Fonagy P, Luyten P, Moulton-Perkins A, Lee YW, Warren F, Howard S, et al. Development and validation of a self-report measure of mentalizing: The Reflective Functioning Questionnaire. PLOS ONE. 2016;11(7):e0158678. doi:
46. Solomon, J. and George, C. (2011 Eds.) *Disorganised Attachment and Caregiving.* Guilford: New York
47. Ahern, K. J. (1999). Ten tips for reflexive bracketing. Qualitative Health Research, 9, 407- 411.
48. CONSORT extension for Randomized Pilot and Feasibility Trials (http://www.consort-statement.org/randomised-pilot-and-feasibilitytrials).

For peer review only

For peer review only - http://bmjopen.bmj.com/site/about/guidelines.xhtml

29 APPENDICES

APPENDIX 1 – SAFE THERAPY SESSIONS

More detailed information about each of the SAFE therapy sessions is as follows:

Session 1 (3 hours in a community session – multi-family therapy)

Families act as 'consultants' for each other and will:

- Present a typical day in their life and the challenges they face
- Explore the understandings of the nature of autism and associated typical challenges and problems
- Draw their family tree and explore with other families their family networks and sources of support
- View videos of typical problematic family scenarios and share practical and emotional coping strategies
- Develop a relational/attachment model of the interaction in the video
- Discuss how this connects with their own experiences of how problems are related to family life
- Learn about and discuss the Circle of Security model
- Discuss coping strategies in terms of parents' own childhood experiences
- Learn about and discuss family and attachment approaches applicable to autism care

Homework task: Identify and draw a problematic cycle identifying actions, thoughts and feelings.

Session 2 (3 hours in family home – whole family session)

Discussion of homework from session 1, followed by:

- As a family draw a picture of a 'day in our life' - discuss the challenges and opportunities they face as a family
- Discussion of a typical problem they have recently had to deal with
- Map the problem or 'meltdown' they have recently experienced and also an example of where things have gone well in the family
- Draw a family genogram together: focus on transitions, similarities and differences between family members parenting styles, attachment patterns, coping with emotional needs and comfort
- Sculpt (using buttons). Mapping of changes in family relationships, intimacy, connections, family patterns a) prior to diagnosis, b). current family configuration, c). ideal/hoped for family configuration. Use this to discuss needs of all the family members, including the siblings.
- Explore the Circle of Security (CoS) to consider their child and their own needs for safety, comfort and exploration. Discuss how parents' childhood experiences shape parenting styles and emotional responses
- Consider systems the child and family are involved in, school, community, clubs, activities, friendships etc. and sources of support and anxieties these can generate. Relate to CoS

Homework task: Identify when child shows 'typical and autistic' behaviour and times they are not sure. Log their responses.

Session 3 (3 hours in family home - whole family session)

Discussion of homework from session 2 followed by:

For peer review only - http://bmjopen.bmj.com/site/about/guidelines.xhtml

- Explore a recent pattern or meltdown and how they have coped
- Self-Autism Mapping (SAM): The child maps which parts of themselves they see as being the same as a typical child and which are influenced by autism. Exploring how the influence of the autism may alter, reduce, expand in different contexts and over the course of development
- Look at Trans-generational pattern of ‘autistic’ traits. How families responded, link to SAM
- Further discussion of the CoS model. Exploration of parents own attachment histories
- Corrective and replicative scripts. What they have learnt from their own childhood. What they want to do differently and similarly. How scripts relate to their responses to the child’s ‘typical’ and ‘autistic’ behaviour
- Mapping of parents’ experience of being parented. Continuing role that their parents (grandparents play in their lives)
- Role-play core part of a typical current problematic sequence at home and explore different ways of managing, especially the importance of naming emotions, feelings at difficult moments.

Homework task: Identify problematic interaction, note reactions in terms of scripts and feelings of helplessness or anger.

Session 4 (3 hours in family home – whole family session)

Discussion of homework from session 3, followed by:

- Map an example of a recent successful and rewarding interaction and also any problems they have faced
- Child with autism and siblings invited to give a ‘presentation’ of an area of their special interest
- Return to a discussion of SAM and understandings of autism – challenges and opportunities
- Discuss parents’ own childhood histories and how they influence their parenting
- Return to a discussion of the family sculpt to consider transitions and changes they will be facing

Homework task: Keep notes on how they support their child regarding attachment dilemmas as in the Story Stems.

Session 5 (3 hours in a community setting – multi-family therapy)

Representatives of local support groups, trained in SAFE will be present at this meeting.

Discussion of homework from session 4, followed by:

- Feedback about how they have been able to use core techniques
- Reflecting discussion from the therapists about their experience of the sessions
- Revisit examples of difficult patterns of interaction – meltdowns and share what they have learnt with each other
- View video of young adult with Asperger Syndrome reflecting on his childhood and how he has learnt to cope
- Discuss similarities/differences in perceptions, changes in relationships, impact of diagnosis, refer to SAM
- Create a future orientated timeline and discuss problems that may arise and how to overcome them

For peer review only - http://bmjopen.bmj.com/site/about/guidelines.xhtml

- Feedback from parents or carers regarding implementing ideas from the programme
- Future challenges and developmental stages that will face the families.
- On-going support available from local voluntary support workers.
- On-going peer support options.

For peer review only

For peer review only - http://bmjopen.bmj.com/site/about/guidelines.xhtml

APPENDIX 2 – AMENDMENT HISTORY

Amendment Number	Protocol Version Number	Date Issued	Author(s) of Changes	Details of Changes Made
1	2	10th January 2018	Helen Hancocks	Front page – addition of REC reference number and ISRCTN number Section 8.2 – Schema updated to clarify that verbal consent will be taken for clinical details for eligibility and confirmation of legal guardianship to be passed from the NHS site to the RA at Plymouth University alongside contact details.<br>Section 11.3.3.2 - updated to clarify that verbal consent will be taken for clinical details for eligibility and confirmation of legal guardianship and will be passed from the NHS site to the RA at Plymouth University alongside contact details. In addition, a checklist will be provided to the clinical team to ensure that verbal consent to

For peer review only

For peer review only - http://bmjopen.bmj.com/site/about/guidelines.xhtml

				forward these details to the research team at Plymouth University is completed correctly.<br><br>Section 17 – removal of the word intervention to clarify that an SAE may have a causal relationship with either arm of the study not just the SAFE family therapy intervention. General updates to clarify the process of SAE reporting to CTU.
2	3	18th June 2018	Helen Hancocks	Front page, section 2, section 3, section 5, section 11.3.1.1, and 27 updated to reflect a change in the name of the sponsor organisation from Plymouth Hospitals NHS Trust to University Hospitals Plymouth NHS Trust<br>Section 2, 11.3.3.2, 13.2 updated to reflect that Plymouth University has changed its name to University of Plymouth

For peer review only - http://bmjopen.bmj.com/site/about/guidelines.xhtml
For peer review only

				Section 1 and section 2 updated to reflect a change in the named Sponsor contact from Lisa Bowern to Corinna Mossop. Section 5, section 8.2 (a tracked changes version of the schema is included in the application), section 11.3.1, section 11.3.1.2, section 11.3.2.2, 11.3.3.1, 11.3.3.2 updated to include community recruitment methods. Section 8 updated to clarify that the trial will end when the last family completes the qualitative feedback. Section 15 added to clarify that the end point of the study is when the last family completes the qualitative feedback.
Non-substantial Amendment 2	4		Helen Hancocks	Section 11.3.1 updated to include a variety of methods for approaching potential participants.
Substantial amendment 3	5		Helen Hancocks	Protocol updated throughout to amending the follow up visit for the intervention

For peer review only

For peer review only - http://bmjopen.bmj.com/site/about/guidelines.xhtml

				arm from 24 to 22 weeks, and overall follow up for all participants from 32 to 24 weeks (±2 weeks). Section 25: Criteria for progression to full trial clarified from “the proportion of families receiving and completing the trial intervention” tp “the extent of families’ engagement in the trial intervention”

For peer review only

For peer review only - http://bmjopen.bmj.com/site/about/guidelines.xhtml

CONSORT 2010 checklist of information to include when reporting a randomised trial*

Section/Topic	Item No	Checklist item	Reported on page No
**Title and abstract**			
	1a	Identification as a randomised trial in the title	1
	1b	Structured summary of trial design, methods, results, and conclusions (for specific guidance see CONSORT for abstracts)	1
**Introduction**			
Background and objectives	2a	Scientific background and explanation of rationale	3
	2b	Specific objectives or hypotheses	3
**Methods**			
Trial design	3a	Description of trial design (such as parallel, factorial) including allocation ratio	4
	3b	Important changes to methods after trial commencement (such as eligibility criteria), with reasons	4
Participants	4a	Eligibility criteria for participants	4
	4b	Settings and locations where the data were collected	4
Interventions	5	The interventions for each group with sufficient details to allow replication, including how and when they were actually administered	4,5
Outcomes	6a	Completely defined pre-specified primary and secondary outcome measures, including how and when they were assessed	5,6
	6b	Any changes to trial outcomes after the trial commenced, with reasons	None
Sample size	7a	How sample size was determined	N/A
	7b	When applicable, explanation of any interim analyses and stopping guidelines	N/A
Randomisation:			
Sequence generation	8a	Method used to generate the random allocation sequence	N/A
	8b	Type of randomisation; details of any restriction (such as blocking and block size)	5
Allocation concealment mechanism	9	Mechanism used to implement the random allocation sequence (such as sequentially numbered containers), describing any steps taken to conceal the sequence until interventions were assigned	N/A
Implementation	10	Who generated the random allocation sequence, who enrolled participants, and who assigned participants to interventions	5
Blinding	11a	If done, who was blinded after assignment to interventions (for example, participants, care providers, those	5

For peer review only

For peer review only - http://bmjopen.bmj.com/site/about/guidelines.xhtml

		assessing outcomes) and how	
	11b	If relevant, description of the similarity of interventions	N/A
Statistical methods	12a	Statistical methods used to compare groups for primary and secondary outcomes	6
	12b	Methods for additional analyses, such as subgroup analyses and adjusted analyses	6
**Results**			
Participant flow (a diagram is strongly recommended)	13a	For each group, the numbers of participants who were randomly assigned, received intended treatment, and were analysed for the primary outcome	See consort diagram
	13b	For each group, losses and exclusions after randomisation, together with reasons	As above
Recruitment	14a	Dates defining the periods of recruitment and follow-up	As above
	14b	Why the trial ended or was stopped	N/A
Baseline data	15	A table showing baseline demographic and clinical characteristics for each group	6
Numbers analysed	16	For each group, number of participants (denominator) included in each analysis and whether the analysis was by original assigned groups	6,7,8
Outcomes and estimation	17a	For each primary and secondary outcome, results for each group, and the estimated effect size and its precision (such as 95% confidence interval)	N/A
	17b	For binary outcomes, presentation of both absolute and relative effect sizes is recommended	N/A
Ancillary analyses	18	Results of any other analyses performed, including subgroup analyses and adjusted analyses, distinguishing pre-specified from exploratory	
Harms	19	All important harms or unintended effects in each group (for specific guidance see CONSORT for harms)	7
**Discussion**			
Limitations	20	Trial limitations, addressing sources of potential bias, imprecision, and, if relevant, multiplicity of analyses	2 and 11
Generalisability	21	Generalisability (external validity, applicability) of the trial findings	10/11
Interpretation	22	Interpretation consistent with results, balancing benefits and harms, and considering other relevant evidence	10/11
**Other information**			
Registration	23	Registration number and name of trial registry	2
Protocol	24	Where the full trial protocol can be accessed, if available	3
Funding	25	Sources of funding and other support (such as supply of drugs), role of funders	

*We strongly recommend reading this statement in conjunction with the CONSORT 2010 Explanation and Elaboration for important clarifications on all the items. If relevant, we also recommend reading CONSORT extensions for cluster randomised trials, non-inferiority and equivalence trials, non-pharmacological treatments, herbal interventions, and pragmatic trials. Additional extensions are forthcoming: for those and for up to date references relevant to this checklist, see www.consort-statement.org.

For peer review only - http://bmjopen.bmj.com/site/about/guidelines.xhtml

BMJ Open

SAFE, a new therapeutic intervention for families of children with autism: a randomised controlled feasibility trial.

Journal:	*BMJ Open*
Manuscript ID	bmjopen-2020-038411.R1
Article Type:	Original research
Date Submitted by the Author:	06-Aug-2020
Complete List of Authors:	McKenzie, Rebecca; University of Plymouth, Institute of Education<br>Dallos, Rudi; Department of Clinical Psychology, University of Plymouth<br>Stedmon, Jacqui; The University of Plymouth, Clinical Psychology<br>Hancocks, Helen; University of Plymouth, Peninsula Clinical Trials Unit<br>Vickery, Patricia; University of Plymouth, Peninsula Clinical Trials Unit<br>Barton, Andy; South West Research Design Service,<br>Vassallo, Tara; University of Plymouth, Institute of Education<br>Myhill, Craig; University of Plymouth, Institute of Education<br>Chynoweth, Jade; University of Plymouth, Medical statistics<br>Ewings, Paul; Research Design Service, Research Office
**Primary Subject Heading**:	Health services research
Secondary Subject Heading:	Paediatrics, Health services research
Keywords:	Child & adolescent psychiatry < PSYCHIATRY, MENTAL HEALTH, Community child health < PAEDIATRICS

SCHOLARONE™
Manuscripts

For peer review only - http://bmjopen.bmj.com/site/about/guidelines.xhtml

I, the Submitting Author has the right to grant and does grant on behalf of all authors of the Work (as defined in the below author licence), an exclusive licence and/or a non-exclusive licence for contributions from authors who are: i) UK Crown employees; ii) where BMJ has agreed a CC-BY licence shall apply, and/or iii) in accordance with the terms applicable for US Federal Government officers or employees acting as part of their official duties; on a worldwide, perpetual, irrevocable, royalty-free basis to BMJ Publishing Group Ltd ("BMJ") its licensees and where the relevant Journal is co-owned by BMJ to the co-owners of the Journal, to publish the Work in this journal and any other BMJ products and to exploit all rights, as set out in our licence.

The Submitting Author accepts and understands that any supply made under these terms is made by BMJ to the Submitting Author unless you are acting as an employee on behalf of your employer or a postgraduate student of an affiliated institution which is paying any applicable article publishing charge ("APC") for Open Access articles. Where the Submitting Author wishes to make the Work available on an Open Access basis (and intends to pay the relevant APC), the terms of reuse of such Open Access shall be governed by a Creative Commons licence – details of these licences and which Creative Commons licence will apply to this Work are set out in our licence referred to above.

Other than as permitted in any relevant BMJ Author's Self Archiving Policies, I confirm this Work has not been accepted for publication elsewhere, is not being considered for publication elsewhere and does not duplicate material already published. I confirm all authors consent to publication of this Work and authorise the granting of this licence.

For peer review only

SAFE, a new therapeutic intervention for families of children with autism: a randomised controlled feasibility trial

Rebecca McKenzie[1], Rudi Dallos[2], Jacqui Stedmon[2], Helen Hancocks[3], Patricia Jane Vickery[3], Andy Barton[4], Tara Vassallo[1], Craig Myhill[1], Jade Chynoweth[5], Paul Ewings[6]

Author affiliations

1. Institute of Education, University of Plymouth, Plymouth, UK
2. Department of Clinical Psychology, University of Plymouth, Plymouth, UK
3. Peninsula Clinical Trials Unit, University of Plymouth, Plymouth, UK
4. South West Research Design Service. Plymouth, UK
5. Medical Statistics, University of Plymouth, Plymouth, UK
6. Research Office, Research Design Service, Taunton, UK

Correspondence to: Dr Rebecca McKenzie rebecca.mckenzie@plymouth.ac.uk

Key words: Family Therapy, autism, intervention, feasibility, mental health

Word count excluding title page, abstract, references, figures and tables: 5534

ABSTRACT

Objectives: To establish the feasibility of a definitive randomised controlled trial of SAFE, an intervention for families of children with autism.

Design: A randomised, controlled, multi-centred feasibility study.

For peer review only - http://bmjopen.bmj.com/site/about/guidelines.xhtml

Setting: Participants were identified from three NHS diagnosing centres in Plymouth, Cornwall and a community pathway.

Participants: 34 families of a child with autism severity level 1 or 2 between 3-16 years. Four families were lost to follow up.

Interventions: Systemic Autism-related Family Enabling (SAFE) is a manualised five-session intervention delivered over 16 weeks, designed for families of children with autism. SAFE involves families attending 3-hour sessions led by systemic practitioners.

Primary and secondary outcome measures: The proposed primary outcome measure was the Systemic CORE-15 (SCORE-15). Proposed secondary outcome measures: Patient Health Questionnaire (PHQ)-Somatic Anxiety Depressive Symptoms, Coding of Attachment-Related Parenting for use with children with Autism (CARP-A), the Child Behaviour Checklist (CBCL), the Reflective Functioning Questionnaire (RFQ), the Caregiving Helplessness Questionnaire (CGHQ). Outcome measures were collected at baseline and 24 weeks post randomisation.

Results: All Primary Caregivers retained in the study completed the SCORE-15 at both time points. 34 of the target of 36 families were recruited and 88% of families retained. Training for therapists was effective. Feedback revealed willingness to undergo randomisation. There was 100% attendance at appropriate sessions for core family members. The SCORE-15 showed reduction in scores for families receiving SAFE compared to controls suggesting positive change. Qualitative data revealed that families found the study acceptable and families receiving SAFE experienced positive change. Feedback indicated that the SCORE-15 should be retained as a primary measure, but secondary measures should be reduced.

Conclusions: This study indicates that a larger trial of SAFE is feasible. Findings suggest that SAFE can address current gaps in recommended care, can be confidently delivered by NHS staff and has potential as a beneficial treatment.

Trial registration number: ISCTRN83964946 IRAS 213527

Ethics The trial protocol received ethical approval from the South West-Exeter Research Ethics Committee (Ref: 17/SW/0192).

For peer review only

STRENGTHS AND LIMITATIONS OF THIS STUDY

- The study addressed a gap in the available research data, and produced important feasibility information to inform a fully powered randomised controlled trial.
- The study explored the feasibility of using measures of family function and a range of mental health measures.
- Quantitative feasibility data were complemented by qualitative focus groups and interviews.

For peer review only - http://bmjopen.bmj.com/site/about/guidelines.xhtml

- The study explored the feasibility of economic analysis measures in a population, which includes adults and their children with developmental disorders.
- The participants were recruited from two NHS Trusts in adjacent counties in the South West of England, leading to potential bias. A future randomised controlled trial will extend to centres across the UK including Scotland and Wales.

For peer review only

INTRODUCTION

More than 1% of the UK population has a diagnosis of autism [1]. Families of children with a diagnosis of autism present complex needs. Children with autism have impairments in social communication and restricted, repetitive behaviours and interests [2]. Autism is widely accepted to have a genetic component and high autistic traits are disproportionally represented among family members [3]. Mental health problems are experienced by more than 70% of individuals with autism and more than 50% of their parents [4,5]. Parents of children with autism are more likely to be hospitalised for mental disorders than parents of typically developing children [6] and mothers of children with autism are reported to have higher unmet needs, more difficulties coping, and lower satisfaction with service interactions than mothers of children with other disabilities [7].

As families of children with autism often exhibit psychological morbidities alongside autism, costs of services to treat these problems are high [8,9]. Furthermore, untreated or unresponsive mental health problems impose societal costs making it hard for parents to interact effectively with services [10], potentially worsening outcomes for children and exacerbating the substantial economic burden of autism [9]. Explanations for high levels of affective disorders in these families include: stress associated with the condition of autism, genetic factors, and intergenerational family dynamics. Parenting children with autism involves stresses associated with challenging behaviour, communicative difficulties, isolation and atypical attachment behaviour displayed by children [11]. In addition, these parents suffer from poorer quality of life, and higher levels of stress compared to other populations due to societal issues including stigma, unemployment and difficulty accessing support [12,13]. Parents of children with autism report that a consequent lack of psychological wellbeing exacerbates maladaptive behaviour in their children [14], which is likely to result in unhelpful cycles of distress and hopelessness.

Previous research demonstrates that experience of trauma and abuse among women is associated with elevated risk of autism developing in their subsequent offspring [15,16]. Hence mothers of children with autism are more likely than the general population to be coping with previous traumatic events. Families of children with autism can experience positive family life, cope well with difficulties and enjoy good relationships with their children [17], but they represent a high-risk group, for whom treatment is disjointed, costly and inadequate [18,19].

A more joined-up approach is required, focusing on communication within families, strategies for coping with challenging behaviour and associated problems and building on strengths and relationships to alleviate mental health difficulties. The current study should be placed in the context of international calls for improved services and care for families of children with autism at country level [20], alongside National Institute of Health and Care excellence (NICE) guidelines and recommendations [21,22], as well as developments regarding children's service provision proposed by the Munroe Report [23,24]. Families of children with autism themselves highlight the importance of professionals working therapeutically with children and the wider family [6].

For peer review only

For peer review only - http://bmjopen.bmj.com/site/about/guidelines.xhtml

SAFE (Systemic Autism-related Family Enabling) is a systemic family therapy approach designed to address autism-related needs including problem-solving, poor socio-communication, mental health difficulties and problematic behaviour [25]. Systemic Family Therapy is a well-recognised, evidence-based psychotherapeutic approach [26]. Despite evidence that family therapies can provide benefits to children with autism and their parents [27,28] its efficacy for treating this condition has not been subject to a randomised controlled trial. In addition, existing research overwhelmingly shows that families of children with autism want interventions which make real improvements to their daily life and sense of wellbeing [29,30].

Patient and Public Involvement

This study grew from the articulated needs of over 90 families surveyed and interviewed through the Plymouth Autism Network. The main aim was to develop a family-orientated support package and conduct a feasibility study, definitive trial and ultimately implement an intervention to be offered to all families after diagnosis. To achieve this aim in a sustainable manner we included families as partners at every stage of design and application.

Prior to commencement of the trial a pilot study was conducted with families acting as consultants. From the pilot we recruited interested families to form the SAFE Family Consultation Group. Their representative was a co-applicant on the initial bid and was employed as a research assistant. The consultation group were consulted at every stage of the trial including the initial application, developing, refining and administering the intervention, developing trial materials, recruitment, training and dissemination.

The family representative attended all trial management and research meetings and took part in all training. She disseminated to consultation group members and where appropriate, meetings with the wider group were held. The input from the families has been invaluable in engaging participants and ensuring their wellbeing.

Patient and Public Involvement (PPI) was an intrinsic part of this study and our continued positive engagement and partnership with families of children with autism is a strength. The research emerged from the difficulties articulated by families and we worked with them to develop solutions. We feel that this way of working made a substantial impact on the outcomes for the families receiving the intervention and the fact that the feedback from families was overwhelmingly positive. We also believe that PPI ensured a more ethical and thorough study which did not lose sight of the ultimate aim. The input of families throughout provided considerable motivation for the research team in delivering the study despite the challenges faced.

Aims and Objectives

The aim of the study was to establish the feasibility of a definitive randomised controlled trial of SAFE.

For peer review only - http://bmjopen.bmj.com/site/about/guidelines.xhtml

Our objectives were to:

1. Demonstrate ability to identify, recruit and randomise eligible families
2. Verify that proposed outcome measures and follow-up were acceptable, and targets for loss to follow-up were achievable
3. Assess adherence of families to the intervention
4. Gather quantitative data on outcomes to inform the design (and sample size) of the future trial
5. Adapt existing resource use questionnaire and assess the feasibility of preference based instruments for this population to facilitate a future economic evaluation
6. As part of the feasibility of process evaluation, collect data on the families' experience of SAFE and the study itself
7. As part of the feasibility of process evaluation, ensure that proposed training arrangements were effective and scalable
8. Provide operational experience to manage the future trial

METHODS AND ANALYSIS

The study comprised a randomised, controlled, multi-centred feasibility study including families of children with a diagnosis of autism [25].

Participants

Participants were identified from three NHS diagnosing centres in Plymouth and Cornwall via a search of clinical records or recruitment directly after diagnosis. A community recruitment pathway was added after commencement of the study to alleviate the burden on clinical staff and increase participation. Families were recruited by posters in local community venues.

Eligibility was determined by diagnosing clinicians or from clinical records or diagnostic letter as well as discussion with the family. A member of the research team gained consent during the first home visit. Pilot data suggested that SAFE was most effective and accessible for children without severe symptoms or an intellectual impairment. Children with severe communication difficulties found it difficult to engage with some SAFE activities. For this feasibility study, therefore, our target population was families of children with autism severity level 1 or 2 with no intellectual impairment.

Eligibility criteria:

• Family included child with autism, aged 3-16 years
• Diagnosis of Autism Spectrum Disorder (ASD) severity level 1 or 2 as documented in DSM-5 [2]
• Diagnosed within 12 months of consenting to the study
• If other diagnoses were present, ASD must be primary diagnosis

For peer review only - http://bmjopen.bmj.com/site/about/guidelines.xhtml

• Family were willing to comply with study requirements

Exclusion criteria:

• Child with ASD severity level 3 as documented in DSM-5 [2]

• Child with ASD and intellectual impairment

• Serious concomitant illness in child or family, or other circumstances such that they were unable to comply with study requirements

• Families who may have been a risk to safety of research staff

• Insufficient English language, or capacity for parent/child to consent/assent to the study

The intervention

SAFE is a manualised intensive programme of systemic family therapy designed to treat maladaptive autistic symptoms and mental health-related difficulties encountered by families of children with autism (See supplementary file - Appendix 1). SAFE lead therapists were qualified NHS systemic practitioners who received an additional 4-day training course in SAFE principles and delivery. Each lead therapist was accompanied by a support therapist trained in SAFE. SAFE provides an array of therapeutic activities based on attachment theory, established systemic practice and the known visual processing preferences of people with autism [31,32]. SAFE is best seen as a toolkit with a variety of activities which can be applied to family therapy flexibly. Activities include visual tasks, drawing, modelling, role-play and tracking circular patterns. Sessions are led by family need and the therapists and family work collaboratively, often in a playful way, using family resources, therapist expertise and the tools that SAFE provides. SAFE draws heavily from well-documented active and playful approaches in family therapy practice and literature [33]. Each therapy session included two therapists with a minimum of intermediate family therapy level of qualification and 4 days training in SAFE principles. Between weeks 1 and 16, families allocated to the SAFE intervention attended five 3-hour SAFE therapy sessions. Sessions 1 and 5 were multi-family sessions and took place in a community setting. Sessions 2, 3 and 4 were for individual families and took place in a community venue or the family home. Following completion of the therapy programme, families attended a group follow-up session at 24 weeks post-allocation. Trained support workers from local voluntary groups attended this follow-up session and gave the families information about continued support through existing networks.

Support as Usually Employed

Families were typically offered a post-diagnosis follow-up appointment with the diagnosing paediatrician. Parents of children whose symptoms were not severe tended to be directed to local authority parenting classes. In some cases families were also directed to relevant resources, for example, The National Autism Society, Gateway ASD and the NHS Child and Adolescent Mental Health Services. For families where a member was experiencing depression or anxiety, treatment varied and was not linked to autism-related care. Referral was often through the GP with patients receiving cognitive behavioural therapy as part of the improved access for psychological therapies service and or medication.

For peer review only - http://bmjopen.bmj.com/site/about/guidelines.xhtml

Procedure

Families were recruited in four cohorts of between 6 and 12 families (two cohorts from Plymouth and two from Cornwall). Post-baseline, once sufficient families had been recruited to establish a cohort, families were randomised 2:1 to the SAFE intervention plus Support as Usually Employed (SAFE+SUE) or Support as Usually Employed only (SUE). Randomisation was undertaken by a member of the Peninsula Clinical Trials Unit (CTU) programming team by means of a 24-hour web-based system created by the CTU in conjunction with a statistician independent from the trial team, and used random permuted blocks, with stratification by study site. Twenty two families were allocated to SAFE+SUE and 12 families were allocated to SUE (See Figure 1). Clinical staff and research staff collecting outcome data were blinded to allocation. Those in the intervention arm received a preparatory home visit from a therapist.

Outcome measure data were collected at baseline and 24 weeks post-randomisation. Additional data were collected from the intervention participants: post allocation, at each therapy session and at the feedback day (See Figure 1).

Feasibility outcome measures

- Ability to identify, recruit and randomise eligible families
- Acceptability of proposed outcome measures and follow-up schedule to participants, and whether targets for loss to follow-up were achievable
- Adherence of therapists and families to the intervention
- Ability to gather quantitative data on outcomes
- Appropriateness of resource use questionnaires and preference-based instruments for this population
- Effectiveness and scalability of training arrangements

Clinical outcome measures

The proposed primary outcome measure was, the Systemic CORE 15 (SCORE-15) [34]. This is a 15-item paper-based survey made up of three subcategories. For each item scores range from 1-5. The potential range of overall scores is 15 to 75, with a lower score indicating higher family functioning. Based on available literature [35], a meaningful change in family function, from pre to post intervention, would be solidly indicated by a reduction of 3 on overall scores. The SCORE is the primary measure of family functioning employed in Children and Young People's Improving Access to Psychological Therapies national programme, and is the gold standard for assessing the impact of family therapy on quality of life in the UK [36]. Every able family member was asked to complete the SCORE-15 at baseline and 24 weeks post-randomisation.

The SCORE-15 is particularly well-suited as a primary measure for this study as it is a measure for the whole family and, like SAFE, it has a systemic framework based on the premise that relational dynamics are central to the wellbeing of all family members. In addition, the three dimensions of SAFE are well matched to

For peer review only

the needs of this population in that the SCORE-15 is sensitive to change in aspects of mental health and problem solving defined by the three sub-categories measuring: family strengths, resilience and coping (*Strengths and Adaptability*); communication and understanding within the family (*Disrupted Communication*) and changes in ability to manage problems (*Overwhelmed by Difficulties*). The SCORE also correlates with measures of parental satisfaction, mental health, strengths and difficulties and life satisfaction [37].

Scores on the proposed secondary outcome measures, which index changes in child behaviour, child-parent attachment, anxiety and depression:

- Patient Health Questionnaire (PHQ)-Somatic Anxiety Depressive Symptoms completed by parents. This comprises the PHQ-9 measuring depression and the generalised anxiety disorder, GAD-7 measuring anxiety [38].
- Adapted mutuality subscale of the Coding of Attachment-Related Parenting for use with children with Autism (CARP-A) [39] completed by the whole family. The CARP-A is a validated observational measure of a child with autism's attachment behaviour towards their carer.
- The Child Behaviour Checklist [40] completed by the Primary Caregiver. This is a 30-item paper-based survey, which detects emotional and behavioural problems.
- The Reflective Functioning Questionnaire [40] completed by parents, measures ability to understand own and others' mental states.
- Caregiving Helplessness Questionnaire (CGHQ) [41] completed by the parents. This is a 26-item questionnaire designed to assess aspects of disorganised caregiving.

Scores on proposed generic health economic outcome measures also provided data on the feasibility of cost-effectiveness analysis:

- EuroQol 5 dimensions, a standardised generic instrument for measuring health outcome.
- Child Health Utility 9 Dimensions, a paediatric generic preference-based measure of health-related quality of life.
- Resource Use Questionnaire (RUQ), a paper-based questionnaire completed by parent about his/her child's use of healthcare and social resources. The RUQ is designed to identify the NHS and social care resource use for the economic evaluation.

Proposed qualitative outcomes assessed via focus groups:

- Acceptability of SAFE and the trial process for participants and therapists
- Reasons for declining and withdrawing from the study

Additional process measures completed after each SAFE session by therapists and families allocated to SAFE:

- The Client Satisfaction Questionnaire (CSQ-8 completed by families)

For peer review only - http://bmjopen.bmj.com/site/about/guidelines.xhtml

- Helpful Aspects of Therapy Questionnaire - assessing what was helpful/unhelpful in each session (HAT completed by families)
- The Training Checklist and Questionnaire monitoring protocol adherence (TCQ completed by therapists)

Quantitative analysis

Prior to analysis a detailed statistical analysis plan was created and agreed by the research team. For each outcome measure, the relevant scores were calculated and presented descriptively by trial arm.
Where available, published guidelines were used to process, score and summarise the measures including the use of imputation in the event of missing items on a questionnaire. Summary measures were calculated as appropriate, for example, means and SD, medians and ranges, numbers and percentages in categories. The only analysis contrasting the two groups was an interval estimate in the form of a 95% confidence interval (CI) for the primary outcome, the SCORE-15.

Qualitative analysis

Prior to analysis a qualitative analysis plan was agreed. Focus group interviews were conducted for SAFE+SUE and SUE families from each cohort after the 24 week outcome data had been collected for that cohort. Focus groups were audio recorded and transcribed verbatim. Consequent qualitative data was managed using proprietary computer-assisted qualitative data analysis software, NVivo 10, and analysed thematically [42,43]. Summaries of process measures were compiled including quantitative data from Likert scales and thematic analysis of open-ended questions. Subsequently, independent analysis of qualitative data was conducted, including notes and memos, on a subset of the data by two of the authors and consensus reached on overarching themes relating to family experience of SAFE.

Place Figure 1. here Participant Flow Diagram (CONSORT)

RESULTS

Recruitment and randomisation

Of 138 families screened, 106 were eligible and 32 ineligible. Forty-five eligible families expressed interest of which 34 were recruited (Figure 1). Since randomisation only occurred once a complete cohort (between 6 and 12 families at each site) was recruited, the time between consent (when baseline questionnaires were

For peer review only - http://bmjopen.bmj.com/site/about/guidelines.xhtml

For peer review only

completed) and randomisation varied. Data on relevant dates were available for 33 families, for whom there was a median (range) of 21 days (2 to 111) between baseline questionnaire completion and randomisation, with a mean (SD) of 34 (32) days. Nineteen families completed the 24-week assessments for which dates were available. Six of the 19 dates were less than 22 weeks from randomisation due to time constraints, none were more than 26 weeks. All Primary Caregivers were mothers and there were 3 single parent families (2 allocated to SAFE+SUE and 1 allocated to SUE). Baseline demographics were similar for children with autism in both groups (See Table 1). Baseline constitution of families varied and are shown in Table 2.

Table 1. Summary statistics of baseline and demographic characteristics for children with autism

	SAFE + SUE (n=22)	SUE (n=12)	Total (n=34)
Age			
Mean (SD)	9.5 (2.7)	9.4 (2.4)	9.5 (2.5)
Median (range)	*10 (5-14)*	*9 (6-13)*	*10 (5-14)*
Gender n (%)			
Male	17 (77)	9 (75)	26 (76)
Female	5 (22)	3 (25)	8 (24)
Ethnicity n (%)			
White	22 (100)	12 (100)	34 (100)

Table 2. Constitution of families at baseline

Family members in addition to child with autism	No. families SAFE+SUE	No. families SUE	No. families total
Mother (only)	2	1	3
Mother + 1 sibling	2	0	2
Mother + 2 siblings	1	0	1
Mother + 3 siblings	2	0	2
Mother + 4 siblings	2	0	2
Mother + Father	0	4	4
Mother + Father + 1 sibling	6	4	10
Mother + Father + 2 siblings	3	1	4
Mother + Father + 3 siblings	1	1	2
Mother + Father + 4 siblings	0	1	1

For peer review only - http://bmjopen.bmj.com/site/about/guidelines.xhtml

Mother + Father + Grandmother + Grandfather	1	0	1
Mother + Father + Grandma + Grandpa + 1 sib	1	0	1
Mother + 3 siblings + 1 nephew	1	0	1

Acceptability of outcome measures and follow-up schedule and loss to follow-up

One SUE family was lost to follow-up and three SAFE+SUE families were lost to follow up (Figure 1). Mechanisms were in place to report and record serious adverse events (SAEs) related to mental health from consent until participants completed the follow-up or withdrew from the study. No SAEs were reported. For the purposes of the study the core family was taken to be the Primary Caregiver (all mothers) and children with autism. In one case parents were joint Primary Caregivers. Families chose who else would attend sessions and complete data. At baseline, 34 families and 122 family members provided data. At the 24-week assessment, 30 families and 97 family members provided data. Different outcomes are completed by different combinations of family members. The Primary Caregiver provided data on all measures at baseline, and 22 of the 23 other caregivers (fathers) did likewise. There was a small number of caregivers who did not provide data at 24 weeks for some questionnaires. The completion of the SCORE by the children with autism and siblings was optional. Reasons for non-completion by the child with autism were: child was too young (n=4), the visit could not be arranged (n=1), the child did not want to (n=1) and reason unknown (n=1) (see Figure 1).

Adherence of families to the intervention

Full engagement with the SAFE intervention involved five 3-hour therapy sessions. The first and last of which were multi-family sessions for the parents (children could attend if they wished) between weeks 1 and 16, with a final feedback day at 22 weeks. The Primary Caregiver was present for these sessions, except for one mother who was absent for session 1, but the father attended. The child with autism was always present at the individual sessions 2-4. Five children were also present at parent sessions 1 and 5.

Quantitative data on outcomes

Results for the SCORE-15, including expressions of uncertainty (95% CI) for estimates by randomised groups are shown in Table 3. In general there is no specific guidance available on how to handle missing data for the outcome measures used. In what follows, a decision was made to use an individual's total score data if there was no more than one missing item, for which the mean of the other items was imputed. If there were totals for subgroups, these were included if there was no more than one missing item within each subgroup, for which the mean of the other items within the subgroup were imputed.

Table 3. Values for SCORE-15, based on the "total" computed for each dimension and overall for each person. (Reduction in values and positive differences in 95% confidence intervals indicate positive change)

For peer review only - http://bmjopen.bmj.com/site/about/guidelines.xhtml

	Baseline				24 weeks post randomisation				Difference^		
	SAFE+SUE		SUE		SAFE+SUE		SUE				
	Mean	SD	Mean	SD	Mean	SD	Mean	SD	95% CI		N
	Strengths and adaptability										
CWA	11.4	3.3	12.8	3.9	12.1	4.0	12.6	4.8	-3.81	3.29	21
Primary CG	10.0	2.7	9.4	2.3	10.9	3.1	10.6	2.3	-1.96	1.92	30
Other*	11.0	3.6	10.2	3.0	11.1	3.9	11.2	4.6	-1.59	3.87	43
Whole family*	10.8	3.3	10.5	3.2	11.2	3.7	11.3	3.9	-0.89	2.07	94
	Overwhelmed by difficulties										
CWA	12.3	3.9	12.3	5.5	13.5	4.2	14.1	5.5	-2.89	5.58	21
Primary CG	12.4	3.5	12.1	4.0	11.5	2.7	12.7	4.1	-0.69	3.79	30
Other*	12.1	3.9	12.2	3.9	11.7	3.2	13.1	4.2	-0.63	3.86	43
Whole family*	12.2	3.7	12.2	4.2	12.1	3.4	13.2	4.4	-0.03	2.96	94
	Disrupted communication										
CWA	12.8	4.3	11.8	4.0	13.2	4.4	11.4	2.9	-5.02	3.08	21
Primary CG	11.9	4.8	9.6	2.8	10.2	2.6	9.6	2.5	-1.95	2.04	30
Other*	12.9	4.6	12.6	4.2	12.3	3.4	11.2	3.6	-2.91	1.59	43
Whole family*	12.6	4.5	11.5	3.9	11.9	3.6	10.7	3.1	-2.03	0.92	94
	Overall Score										
CWA	36.4	10.1	36.8	11.9	38.8	11.3	38.1	12.0	-9.68	11.22	21
Primary CG	34.3	9.7	31.1	7.6	32.5	7.2	33.0	7.9	-3.49	7.35	30
Other*	36.1	10.2	34.9	9.5	35.1	7.5	35.5	10.4	-3.81	7.24	43
Whole family*	35.7	9.7	34.1	9.5	35.2	8.6	35.2	9.8	-2.09	5.26	94

* scores averaged across all relevant family members

^ difference between SUE and SAFE + SUE at 24-week follow up, adjusting for baseline in ANCOVA; based on individuals with complete data at both baseline and 24-week assessment; no allowance for hierarchy of data

CG = Caregiver

CWA = Child with a diagnosis of autism

For peer review only

Feasibility of using economic evaluation instruments for this population

Completeness of health economics data was achieved in intervention and control groups for the primary care based, community based and mental health based services at 100% response rate at baseline dropping to 82% and 83% at 24 weeks in intervention and control groups, respectively. The outpatient based services had response rates of 68% and 67% at baseline, but completeness improved to 82% and 83% in intervention and control groups respectively at 24 weeks.

For EQ-5D-5L, at baseline the control group had a 100% response rate. At 24 weeks the response rates were 82% and 83% of participants in intervention and control groups, respectively.

As expected CHU-9D responses were largely provided by children with autism (completed by the Primary Caregiver where necessary). Response rate in the intervention group fell from 77% to 73% for children with

For peer review only - http://bmjopen.bmj.com/site/about/guidelines.xhtml

autism but remained constant with three (14%) Primary Caregiver responses at baseline and 24 weeks. In the control group, response rate fell from 83% to 58% in children with autism and 25% to 17% in Primary Caregivers (mothers) from baseline to week 24. Despite indications for increased appropriate service use coupled with reduced cost over time for SAFE+SUE children with autism, the economic evaluation focused only on children with autism, consequently insufficient data was collected for whole family service use and costs.

The families' experience of the study and the SAFE intervention

Qualitative data on the experience of the study were collected from SAFE+SUE and SUE families in family feedback days for each cohort. Key areas revealed by analysis of the data were a) the study was not burdensome, b) that they took part to help others, c) research staff made completing tasks easier and sessions could be organised differently.

The families did not find the completion of CARP-A Lego task and questionnaires onerous:

> *"didn't mind so much doing like the Lego task I mean like it's something that we like to do anyway … but um the questionnaires didn't have a problem with those either"*

Families felt that there were too many questionnaires, but the approachable nature of the research staff made the task easier:

> *"it was all fine … it was A [Research Assistant] that came in did it and she's so friendly and approachable that it made it easy…"*

Families generally took part in the study for altruistic reasons. They wanted to be part of a study which could potentially increase support post-diagnosis. Feedback from SUE families suggested that this helped them to accept their role in the study:

> *" I thought actually this could be a way of helping future parents of diagnosed children to get the support and advice that wasn't there when I got the diagnosis"*

The focus group data also revealed that families felt sessions at home were too distracting, the sessions should be shorter, but more of them and more activities for younger children were needed.

Where families were allocated to the intervention, data on family experience of SAFE were collected via process evaluation measures, the Helpful Aspects of Therapy Questionnaire (HAT) and the Client Satisfaction Questionnaire (CSQ) completed by family members after each therapy session and via the family feedback day. Quantitative data from the HAT indicated that SAFE families found the intervention

For peer review only - http://bmjopen.bmj.com/site/about/guidelines.xhtml

helpful with mean scores for all sessions on the HAT being rated by children and adults as helpful or very helpful. Analysis of all qualitative data from process measures and the feedback day resulted in the following overarching themes: therapist helping reflection, increased understanding, feeling closer, feeling more confident, more able to reflect and problem solve, improved communication and feeling less isolated.

The multi-family sessions made families feel less alone, more able to talk about difficult issues and less judged.

> *"Talking to other parents and feeling listened to and supported"*

> *"Talking about daily issues and sharing these with the other parents, generally feeling that I am not alone"*

Families reported being able to step back and reflect, leading to more confidence in tackling family problems.

> *"Breaking down problem events and looking at causes, outcomes, choices made. Compared a recent to past events and saw progress and maturity in decision making and actions"*

SAFE therapists were seen as allies and the activities helped with family problems leading to increased understanding and improved mental health:

> "*Actually being able to hear that I'm doing OK and actually not as bad as what I thought I was, was nice. It was something that helped me with my mental and emotional state"*

Effectiveness and scalability of training

The therapists completed the Training Checklist Questionnaire (TCQ) after each session. The TCQ was a short questionnaire documenting adherence to planned activities in each session, confidence and ease of delivering activities, rating of the effectiveness of activities and any additional resources required. Focus groups and interviews were also conducted with the therapists after completion of the intervention. The therapists felt that SAFE was an inspirational and effective intervention, was non-pathologising and child centred. The training was motivational and thorough. In 82% of sessions lead therapists reported feeling confident, that the sessions were effective and the activities were easy to deliver. The support therapists, who were less experienced, felt confident, that sessions were effective and activities easy to deliver in 63% of sessions. The therapists felt supervision was very helpful. They highlighted that the gap between training and intervention was too long.

Operational experience moving forward.

For peer review only - http://bmjopen.bmj.com/site/about/guidelines.xhtml

For peer review only

The Chief Investigator (CI) kept the study on time and within budget despite multiple challenges. The support and advice of the Trial Steering Committee (TSC), the PenCTU and the research team facilitated the development of the skill set of the CI in preparation for a definitive trial.

DISCUSSION

To our knowledge, this study is unique in evaluating the feasibility and efficacy of a Family Therapy-based intervention designed specifically for families of children with a diagnosis of autism. The results indicate that a larger trial of SAFE would be feasible. The findings also suggest that offering SAFE for families post-diagnosis is both feasible and potentially efficacious.

Progression to a definitive trial was supported based on pre-determined criteria met in the following ways: a) 34 of the target of 36 families were recruited. Family feedback revealed willingness to undergo randomisation as families felt the study had potential to extend services. b) 88% of families were retained. c) There was 100% attendance at appropriate sessions for core family members. d) For the SCORE-15, complete data were available at both time points and for every dimension for 88% Primary Caregivers, 62% children with autism and 55% other family members. All Primary Caregivers retained in the study completed the SCORE-15 at both time points. e) Collecting health and social care resource utilisation data and health-related quality of life data for children with autism was feasible. f) Process evaluation and feedback day showed that in 82% of sessions lead therapists felt confident, that sessions were effective and easy to deliver. g) The qualitative and process data from families indicates tentative potential for efficacy as SAFE was rated as helpful or very helpful in addressing problems. Qualitative analysis revealed the following themes of positive change: therapist helping reflection, increased understanding, feeling closer, feeling more confident, more able to reflect and problem solve, improved communication and feeling less isolated. A common thread across all qualitative data was increased mental wellbeing among family members receiving SAFE. Although we acknowledge that the current study was not powered to detect a difference, the SCORE-15 showed marked reduction in scores for SAFE+SUE suggesting positive change. A reduction of this magnitude indicates tentative potential proof of efficacy [34,35] (See Figure 2).

Place Figure 2. here.

Additional analysis of the SCORE-15 data revealed that the most marked indications of positive change were experienced by Primary Caregivers with high anxiety as measured by the GAD-7 scale. The

For peer review only - http://bmjopen.bmj.com/site/about/guidelines.xhtml

relationship between anxiety scores and values on the SCORE-15 is important as SAFE seeks to alleviate mental health issues. An indication that a reduction in anxiety and improved family function can be captured by our proposed measures and that data suggests positive change for those participants with acute difficulties receiving the intervention is therefore promising (See Figure 3).

Place Figure 3. here.

The sample size calculation for a subsequent fully powered randomised controlled trial is based on the SCORE-15 for Primary Caregivers, which will be the primary outcome in the full trial. The minimal clinically important difference of 3 is based on the literature [35] and an SD of 8 is based on an SD of 7.3 obtained from the feasibility study. To account for a possible group effect in the intervention group we will use a conservative 0.1 for the intra-group correlation and an assumed mean group size of 6 with variance 2. Employing the conventional two-sided 5% alpha and 90% power, and retaining the 2:1 allocation, gives a requirement for 405 evaluable participants. Using the drop-out experienced in the feasibility study would increase the requirement to 460.

The feasibility study alongside previous research [4,5,6,7] strongly suggests that these families are an at-risk group who experience very limited support post diagnosis. One of the primary aims will be to carry out a definitive trial in order to address international [20] and national recommended good practice in relation to children with autism and their families [NICE guidelines 21,22]. These recommend psychosocial interventions, which improve sensitivity, responsiveness and communication within families. They also call for interventions which prevent challenging behaviour by creating care packages which address co-existing mental health problems, and provide support for families. NICE guidelines associated with child mental health also ask for the provision of psychological therapies including family therapy; the need for parents' psychiatric problems to be treated, for children’s mental health to improve; and management of developmental conditions to be in parallel with mental health interventions.

NICE research recommendations associated with autism call for randomised controlled trials exploring family interventions that are designed to reduce challenging behaviour, alleviate stress and improve quality of life [21]. Our long-term goals are also informed by the Munroe Report [44], which called for children’s services to offer family-centred, therapeutic intervention rather than risk management. Additionally, the current implementation of the Improved Access to Psychological Therapies scheme for children [45] promotes proactive attachment-based and family therapy approaches. SAFE has the potential to address this gap by providing a family-based intervention which is sustainable, extends existing services and is beneficial to families.

For peer review only - http://bmjopen.bmj.com/site/about/guidelines.xhtml

LIMITATIONS

Use of the primary measure the SCORE-15 was limited for children below 7-years and an adapted more visual version will be developed so children below 7-years can contribute. The economic evaluation focused solely on the child with a diagnosis. To ensure sufficient data is collected, a subsequent trial should include longer-term service use and costs for all family members. Families felt there were too many measures and repetition. For any subsequent trial the outcome measures would be reduced and adapted. The Reflective Functioning Questionnaire (RFQ) replicated information from other measures and the Child Behaviour Checklist (CBCL) was lengthy and complex. These measures would not be used in a subsequent trial. We propose the SCORE-15 (measuring perceived change in family functioning) be retained as our primary outcome measure for Primary Caregiver alongside secondary measures being the adapted SCORE-15 (for other family members including the youngest children), PHQ-SADS (anxiety scale), CGHQ (measuring change in caregiver helplessness) and the CARP-A (measuring mutuality between family members).

Recruitment and organisation of SAFE sessions was challenging. There was large variability in the timescales between baseline and start of intervention. In any subsequent trial waiting could be managed by carrying out baseline measurement once enough families have consented for randomisation to occur. Therapists and families preferred shorter sessions in community venues. A recruitment open day, screening tool and appointments for SAFE sessions based on a clinic model with predetermined shorter appointments are proposed for any subsequent trial. Families of children with autism were not eligible if their children had intellectual disability or needed substantial support. Subsequent refinements of the intervention should address this issue to broaden the application of the intervention. Due to the lack of ethnic diversity in Plymouth and Cornwall our participants were all white. This will need to be addressed in any future trial. Given that prevalence differs between ethnic groups [46,47,48] and beliefs and support networks may also differ, it is essential for appropriate implementation across the UK, and internationally, that any future trial reflects ethnic diversity. Any definitive trial will, therefore, include centres which have an ethnically diverse client base.

CONCLUSIONS

To our knowledge this is the first study of Family Therapy as an intervention for families of children with a diagnosis of autism. The proposed primary outcome measure, the SCORE-15, indicated potential benefits for the intervention group compared to controls in coping with problems, developing strengths and communicating within families. Qualitative data showed that family members felt the intervention improved understanding, communication, confidence, mental health, reflection, problem solving and sense of isolation. Attendance and completion of outcome measures was acceptable and NHS therapists were

For peer review only - http://bmjopen.bmj.com/site/about/guidelines.xhtml

effectively skilled after receiving brief SAFE training. This study suggests that SAFE can address current gaps in recommended care post-diagnosis and can and should be subject to a definitive trial. The findings indicate that SAFE can be effectively and confidently delivered by existing NHS staff and has potential to be a feasible and beneficial treatment for these families.

Acknowledgements: The authors would like to thank the SAFE Family Consultation Group for their input and expertise and all of the participating families from whom we learnt so much. The authors would like to thank James Cook, Mary Hosken, Dr Ben Whalley, Dr Antonieta Medina-Lara and Professor Julian Archer for their help in developing and conducting this study. The authors would also like to give special recognition to the dedication, hard work and sensitivity of Helen Hancocks.

Contributors: RM, PJV and HH were responsible for the overall development of the study and trial management. RM, HH, RD, CM, PE, AB, TV and the Family Consultation Group were involved in the conception, administration and conduct of the study. PE, JC and AB provided methodological expertise and advice on quantitative analysis, PE and JC provided statistical expertise and analysis. RD was the lead researcher, with the support of JS, on design of the intervention, supervision of therapists and qualitative analysis. TV, with the support of the Family Consultation Group, and CM advised on design, ethics and data collection particularly from the participant perspective. All authors made substantial contributions to drafting, revision and approval of this document.

Funding: This work was supported by the National Institute for Health Research (NIHR), grant number: PB-PG-0815-20058, and by Autistica, grant number: 7239

Competing interests: Contributors are co-applicants or employed research staff on the SAFE project, which receives funds from both NIHR and Autistica. Professor Rudi Dallos holds joint Intellectual Property rights for the SAFE intervention with the University of Plymouth.

Data sharing statement: Individual participant data that underlie the results reported in this article will be available on request from the lead author and the research Sponsor (University Hospitals Plymouth NHS Trust). De-identified participant data (including text, tables, figures and appendices) will be available from 9 months following publication of the article. Data will be shared with (or access to the data will be provided to) Investigators whose proposed use of the data has been approved by the Chief Investigator and Sponsor. In addition, the study protocol will be available on request. For information regarding submitting proposals and accessing data please contact the lead author rebecca.mckenzie@plymouth.ac.uk

REFERENCES

1. National Autistic Society, *Autism Facts and History*, 2018

For peer review only - http://bmjopen.bmj.com/site/about/guidelines.xhtml

For peer review only

2. *American Psychiatric Association. Diagnostic and Statistical Manual of Mental Disorders,* 5th edn. Washington, DC: American Psychiatric Association, 2013.

3. Sasson NJ, Lam KSL, Parlier M, et al. Autism and the broad autism phenotype: familial patterns and intergenerational transmission. *J Neurodev Disord* 2013;5:11-18.

4. Dykens EM, Fisher MH, Taylor JL, et al. Reducing Distress in Mothers of Children with Autism and Other Disabilities: A Randomized Trial. *Pediatrics* 2014; 2013-3164.

5. Hallett V, Ronald A, Colvert, E, et al. Exploring anxiety symptoms in a large-scale twin study of children with autism spectrum disorders, their co-twins and controls. *J Child Psychol Psychiatry* 2013;54:1176-1185.

6. Daniels JL, Forssen U, Hultman M, et al. Parental Psychiatric Disorders Associated With Autism Spectrum Disorders in the Offspring. *Pediatrics* 2008;121:1357-1162.

7. Siklos S, Kerns KA. Assessing Need for Social Support in Parents of Children with Autism and Down Syndrome. *J Autism Dev Disord* 2008;36:921-933.

8. McCrone P, Dhanasiri S, Patel A, et al. *The cost of mental health care in England to 2026*. Executive Summary, London: The King's Fund. 2008

9. Buescher AVS, Cidav Z, Knapp M, et al. Costs of Autism Spectrum Disorders in the United Kingdom and the United States. *JAMA Pediatr* 2014;e1-e8.

10. Osborne LA, McHugh L, Saunders J, et al. Parenting Stress Reduces the Effectiveness of Early Teaching Interventions for Autistic Spectrum Disorders. *J Autism Dev Disord* 2008;38:1092-1103.

11. Pisula E. *Parenting Stress in Mothers and Fathers of Children with Autism Spectrum Disorders,* A Comprehensive Book on Autism Spectrum Disorders, Dr. Mohammad-Reza Mohammadi (Ed.) 2011. Available from: http://www.intechopen.com/books/a-comprehensive- bookon- autism-spectrum-disorders/parenting-stress-in-mothers-and-fathers-of-children-with-autismspectrum-disorders

12. Vasilopoulou E, Nisbet J. The quality of life of parents of children with autism spectrum disorder: A systematic review. *Res Autism Spectr Disord* 2016;23:36-49.

13. Ludlow A, Skelly C, Rohleder P. Challenges faced by parents of children diagnosed with autism spectrum disorder. *J. Health Psychol* 2012;17(5):702-11.

14. Nikmat AW, Ahmad M, Oon NL, et al. Stress and psychological wellbeing among parents of children with autism spectrum disorder. *ASEAN J of Psychiatr* 2008;9:65-72.

15. Roberts AL, Koenen KC, Lyall K, Ascherio A, Weisskopf MG. Women's posttraumatic stress symptoms and autism spectrum disorder in their children. *Res Autism Spectr Disord* 2014;8(6):608-616.

16. Roberts AL, Lyall K, Rich-Edwards JW, Ascherio A, Weisskopf MG. Maternal exposure to intimate partner abuse before birth is associated with autism spectrum disorder in offspring. *Autism* 2016;20(1):26-36.

17. Markoulakis R, Fletcher P, Bryden P. Seeing the glass half full: benefits to the lived experiences of female primary caregivers of children with autism. *Clin Nurse Spec* 2012;26:48–56.

18. Murphy M, Bolton PF, Pickles A, et al. Personality traits of the relatives of autistic probands. *Psychol Med* 2000;30:1411-1424.

19. Blackledge JT, Hayes SC. Using Acceptance and Commitment Training in the Support of Parents of Children Diagnosed with Autism. *Child Fam Behav Ther* 2006:28:1-18.

20. World Health Organization. WHA67. 8 Comprehensive and coordinated efforts for the management of autism spectrum disorders 2014.

21. NICE guidelines [CG170] https://www.nice.org.uk/guidance/cg170/chapter/patient-centred-care

22. NICE guidelines [CG128] https://www.nice.org.uk/guidance/cg128

For peer review only - http://bmjopen.bmj.com/site/about/guidelines.xhtml

23. Monroe E. *The Munroe Review of Child protection: Final report,* Dept. of Education, HM Stationary Office, UK 2011.
24. Goodman S, Trowler I. *Social work re-claimed: Innovative frameworks for child and social work Practice*. London: Jessica Kingsley 2012.
25. McKenzie R, Dallos R, Stedmon J, Hancocks H, Vickery PJ, Ewings P, Barton A, Vassallo T, Myhill C. SAFE, a new therapeutic intervention for families of children with autism: study protocol for a feasibility randomised controlled trial. *BMJ Open* 2019:9:5:e025006.
26. Stratton P. Report On The Evidence Base Of Systemic Family Therapy. *Association for Family Therapy* 2005.
27. Monteiro M. *Family Therapy and the Autism Spectrum: Autism Conversations in Narrative Practice*. London: Routledge 2016.
28. Estrada AU, Pinsof WF. The effectiveness of family therapies for selected behavioural disorders of childhood. *J Marital Fam Ther* 1995:21: 403-424.
29. Pellicano E, Dinsmore A, Charman T. What should autism research focus upon? Community views and priorities from the United Kingdom. *Autism, Special Issue Article* 2014:1-15.
30. Wallace S, Parr J, Hardy, A. *One in a hundred: Putting families at the heart of autism research*. London: Autistica 2013.
31. Buron KD, Wolfberg P. *Learners on the autism spectrum: Preparing highly qualified educators*. Shawnee: AAPC Publishing, 2008:114–37.
32. Grandin T, Scariano MM. *Emergence: Labeled autistic*. New York, NY: Warner Books, 1986.
33. Dallos R, Draper R. *An introduction to family therapy: Systemic theory and practice*: McGraw-Hill Education, 2015.
34. Stratton P, Bland J, Janes E, *et al*. Developing an indicator of family function and a practicable outcome measure for systemic family and couple therapy: the SCORE. *J Fam Ther* 2010;32:232–58.
35. Carr A, Stratton P. The score family assessment questionnaire: A decade of progress. *Fam. Process* 2017;56(2):285-301.
36. SHIFT Self-Harm Intervention, Family Therapy: a randomised controlled trial of family therapy vs. treatment as usual for young people seen after second or subsequent episodes of self harm. Protocol Grant Number: 470772 HTA Reference Number: 07/33/01. 2015.
37. Stratton P, Lask J, Bland J, Nowotny E, Evans C, Singh R, Janes E, Peppiatt A. Detecting therapeutic improvement early in therapy: validation of the SCORE-15 index of family functioning and change. *J Fam Ther* 2014; 36:1:3-19.
38. Spitzer RL, Kroenke K, Williams JBW. Validation and utility of a self report version of PRIME-MD: the PHQ Primary Care Study. *JAMA* 1999;282:1737–44.
39. Blazey L. *Coding of Attachment-Related Parenting for use with children with Autism (CARP-A)*. Manchester: Manchester University, 2007.
40. Moss E, Bureau JF, Cyr C, *et al*. Correlates of attachment at age 3: construct validity of the preschool attachment classification system. *Dev Psychol* 2004;40:323–34.
41. Solomon J, George C, eds. *Disorganised Attachment and Caregiving*. Guilford: New York, 2011.
42. Braun V, Clarke V. Using thematic analysis in psychology. *Qual Res Psychol* 2006;3:77–101.
43. Green J, Thorogood N. *Qualitative Health Methods for health Research*. London: Sage Publications, 2004.
44. Munroe E. *The Munroe Review of Child protection: Final report,* Dept. of Education, HM Stationary Office, UK 2011.
45. Department of Health. *IAPT Implementation Plan: National Guidelines for Regional Delivery*. 2008. Available at www.iapt.nhs.uk

For peer review only

For peer review only - http://bmjopen.bmj.com/site/about/guidelines.xhtml

46. Keen DV, Reid FD, Arnone D. Autism, ethnicity and maternal immigration. *BJPsych* 2010;196:4:274-81.

47. Mandell DS, Wiggins LD, Carpenter LA, Daniels J, DiGuiseppi C, Durkin MS, Giarelli E, Morrier MJ,. Nicholas JS, Pinto-Martin JA, Shattuck PT. Racial/ethnic disparities in the identification of children with autism spectrum disorders *Am J Public Health* 2009;99:3:493-8.

48. Barnevik–Olsson M, Gillberg C, Fernell E. Prevalence of autism in children born to Somali parents living in Sweden: a brief report *Dev Med Child Neurol* 2008;50:8:598-601.

Figure Legends

Figure 1. Participant Flow Diagram (CONSORT)

Figure 2. Mean change in SCORE-15 subcategories for SAFE+SUE (SAFE) and SUE only (Control) for all family members including children with autism

Figure 3. SCORE-15 total mean change for Caregivers with low and high anxiety (a reduction = positive change; high anxiety > 7 on GAD-7)

For peer review only

For peer review only - http://bmjopen.bmj.com/site/about/guidelines.xhtml

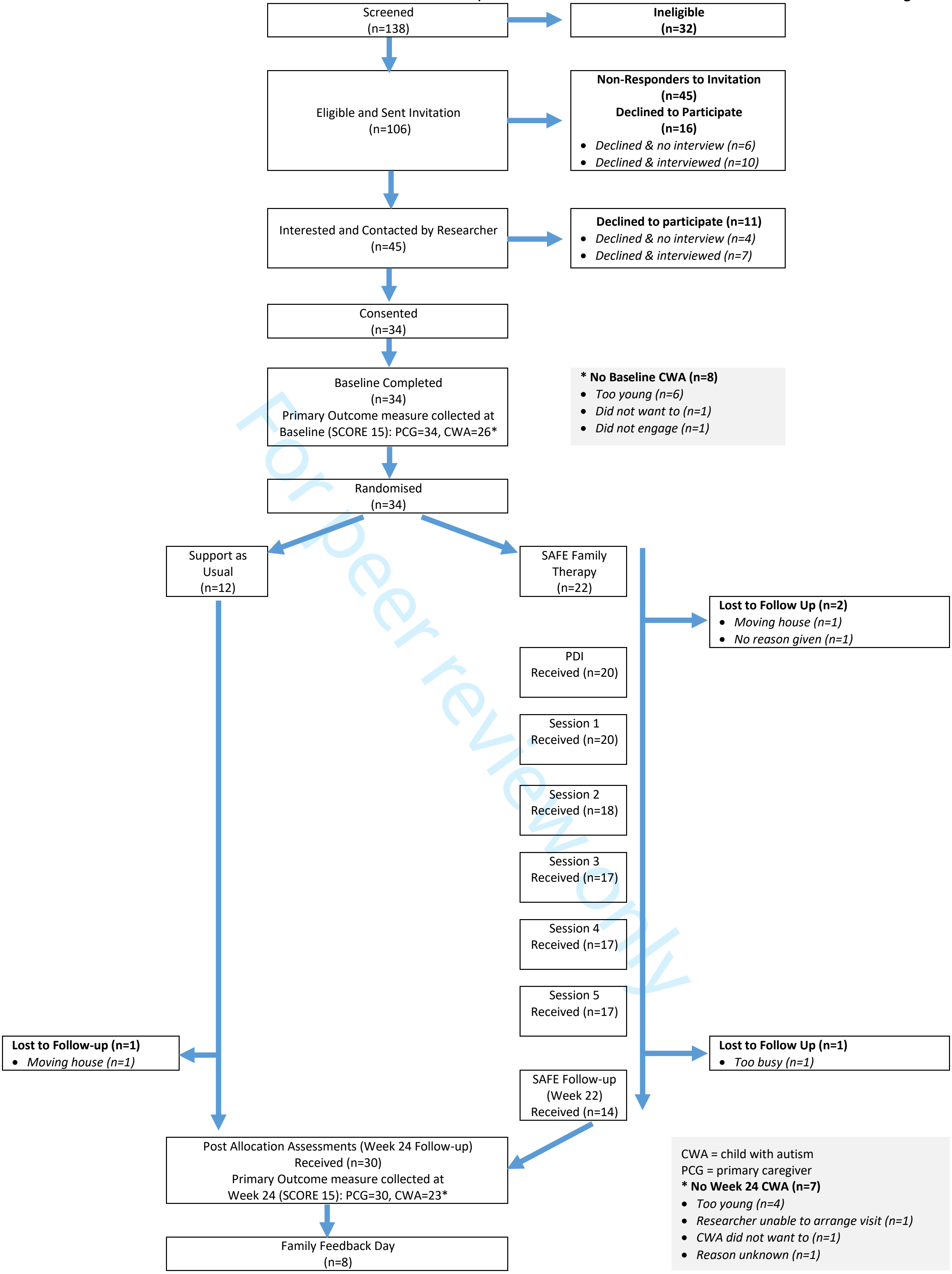

Figure 1. Participant Flow Diagram (CONSORT)

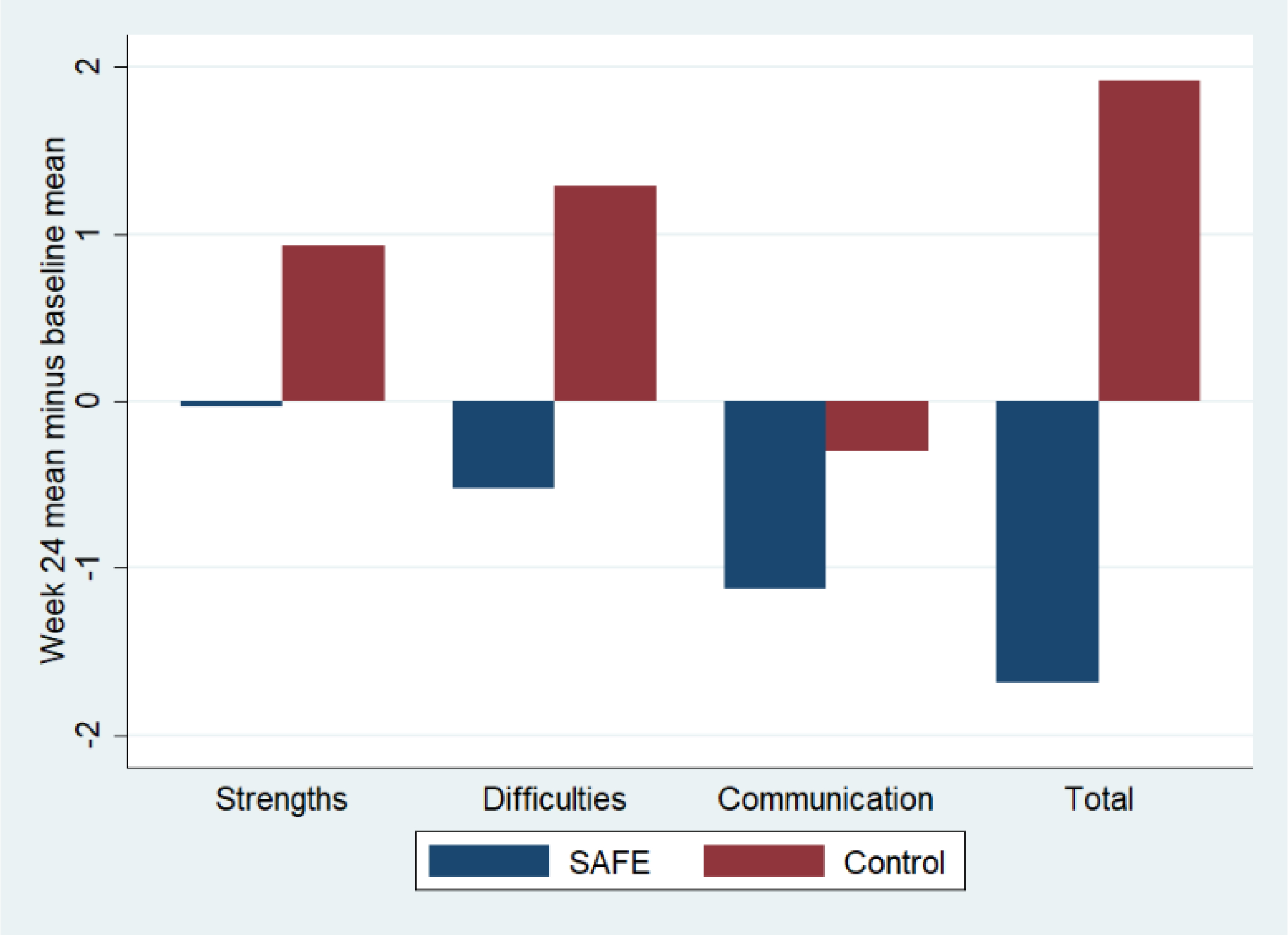

Figure 2. Mean change in SCORE-15 subcategories for SAFE+SUE (SAFE) and SUE only (Control) for all family members including children with autism.

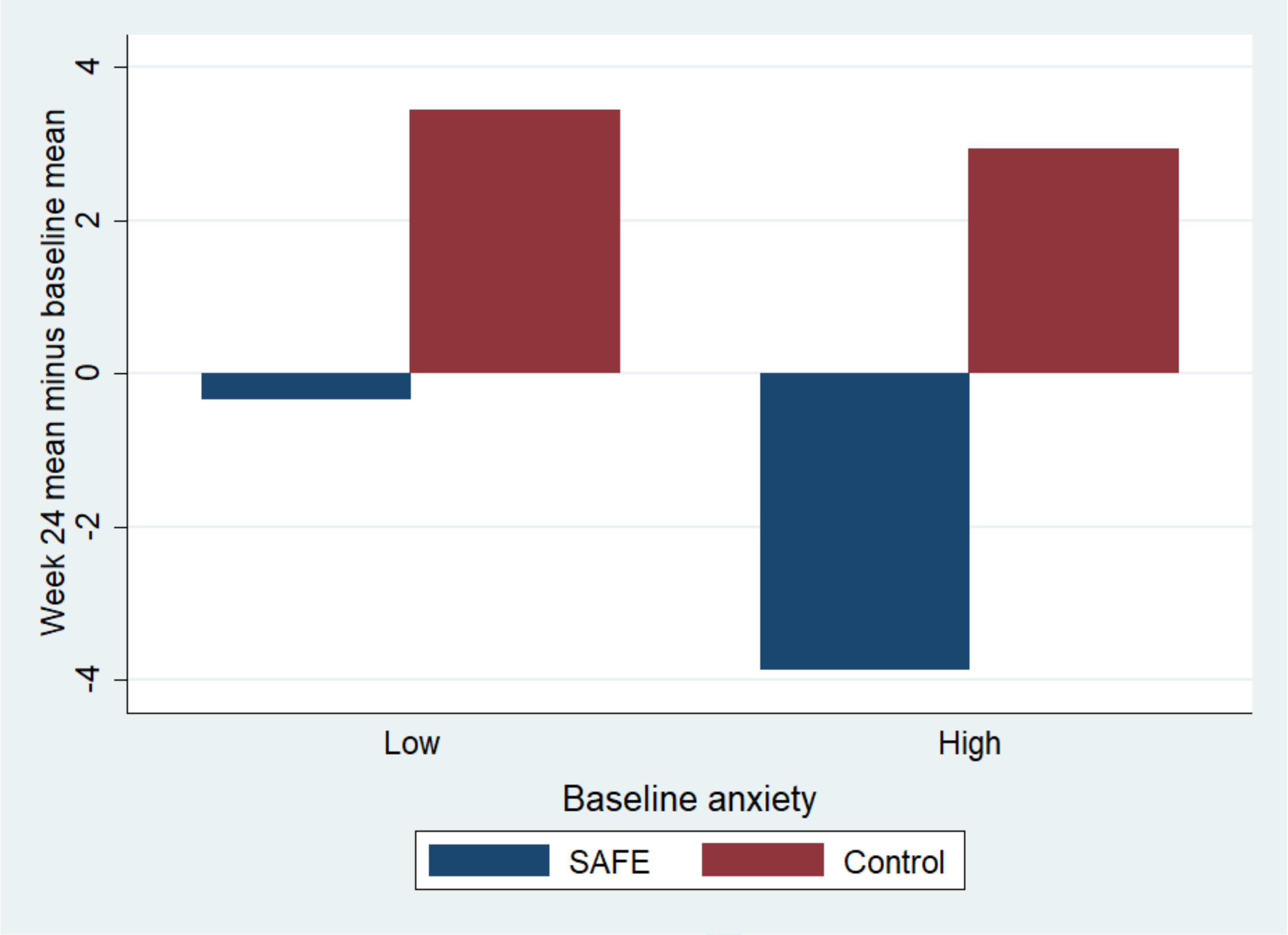

Figure 3. SCORE-15 total mean change for Caregivers with low and high anxiety (a reduction = positive change; high anxiety > 7 on GAD-7).

Appendix 1
Brief Summary of INTERVENTION MANUAL for SAFE (Systemic Autism-related Family Enabling): an early intervention for families of children with autism and Asperger Syndrome

Professor Rudi Dallos and Doctor Rebecca McKenzie

The intervention manual has been developed from the following bases of evidence and clinical experience:

1. Systemic Family Therapy.
Systemic Family Therapy focuses on promoting positive changes in the relationships within families rather than the isolated behavior of individuals in the family (Dallos & Draper, 2015). Difficulties are seen to be maintained or exacerbated by family dynamics including the organization of families, communicational patterns and repetitive interaction sequences of problems. The combination of these is encapsulated in the idea of the formation of ineffective attempted solutions to their problems which rather than offering solutions can aggravate their difficulties. Systemic Family Therapy embodies a range of approaches and techniques for helping families to explore and re-organise their understanding, relationship patterns, emotional connections and problem-solving abilities. It typically involves a therapist and a supervision team working with one family at a time with close monitoring, based on feedback during and at the end of each session exploring the relevance, helpfulness and applicability of the therapy. Where supervision teams are not available a more flexible model - 'in room consultation' can be employed whereby two therapists support each other in a structure where one therapist takes on the role of monitoring the family's reactions as therapy proceeds and periodically offering feedback as a form of live supervision. A development of this orientation is Multi-Family Therapy whereby groups of families work together to assist and facilitate change, effectively acting as consultants for each other. The SAFE program employs both these versions of Systemic Family Therapy with three single family sessions to explore in detail each family's needs; and two group sessions using concepts and techniques from Multi-Family Therapy

2. Multi-Family Therapy
Multi-Family Therapy is a recognised treatment approach which aims to provide a more empowering, flexible and intensive form of family intervention than single family therapy (Asen & Scholz, 2009). In Multi-Family Therapy similar techniques are employed to those used in single family therapy, but families are encouraged to work together, to be proactive in solving their own problems Multi-Family Therapy aims to help families rediscover their own resources by emphasising how families can take an active role in tackling dilemmas and assisting each other. At the same time families are encouraged to use the group setting to explore how problems have affected family life in consultation with other families and to share their solutions and competencies. This involves a power shift from the therapist-client relationship and encourages an empowering peer-support environment. The sharing of experiences and the dynamics of the group are important components of the treatment.

3. Family Models
The SAFE program incorporates a range of concepts and techniques by integrating systemic and attachment based family models, in particular The Circle of Security Intervention (Powell et al., 2014) and Attachment Narrative Therapy developed by co-applicant Professor Rudi Dallos (Dallos, 2006). In the SAFE intervention, these systemic and attachment models have been combined with an emphasis on visual materials and active, play-based approaches designed to be appropriate for families of children with autism.

For peer review only - http://bmjopen.bmj.com/site/about/guidelines.xhtml

4. Autism friendly
SAFE has been developed in collaboration with children with autism and their families to be autism friendly. Given the evidence in the literature for a visual processing style, communication difficulties and restricted interests among people with autism, the advice of families is in line with current research. SAFE incorporates a multi-sensory approach using visual materials which are integrated into therapeutic techniques and play-based activities. SAFE also acknowledges and works with the children's talents and areas of special interest. It also recognizes that parents are often extremely well-informed and prefer an approach which recognizes their competencies and helps them to feel empowered rather than de-skilled by therapy.

SAFE sessions and examples of activities:

SAFE involves 5 x 3-hour sessions outlined in a detailed manual. All sessions are facilitated by two therapists trained in the SAFE intervention model. Around 6 families receive the SAFE intervention as a cohort, supported by two therapists. Sessions 1 and 5 are based on Multi-Family Therapy and all parents from the 6 families attend. Sessions 2, 3 and 4 are for single families and all family members attend including siblings and/or grandparents if the family wish. So the 6 families meet each other at the beginning and the end but the in-between sessions are just for their own family.

SAFE is supported by a detailed manual setting out a structure for each session and also a toolkit of activities which can be used flexibly according to family need and the age and symptoms of the child.

Examples of activities include the following:

Tracking a circularity

Families explore the events of positive and problematic cycles of events, such as 'meltdowns' by breaking these down to explore in detail what happened, including actions, understandings, expectations and how people felt. E.g. what happened first, then what happened next? What did you do? What did Mum do? This can allow families to unpick problematic and successful cycles and initiate positive change.

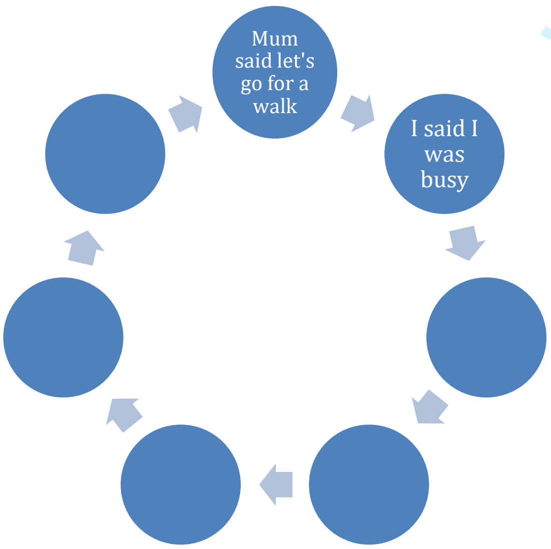

For peer review only

A family genogram

The families are asked to create a genogram (family tree) this is used to explore relationships, perceptions of autism, family narratives and avenues of support and conflict. E.g. who is most supportive to you? Who also has autistic traits? Who is most similar to you? What are the stories you have heard from grandparents etc.

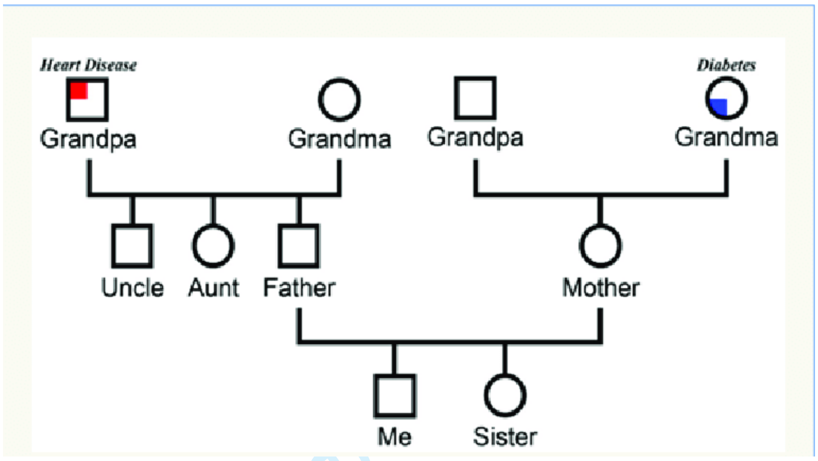

The Circle of Security (COS)

The COS intervention and the graphic designed around it are designed to help parents increase awareness of their children's needs and whether their own responses meet those needs. With increased awareness parents can expand their moment-to-moment parenting choices where needed. The model, therefore, encourages the potential to break the stranglehold of problematic patterns. Discussion can include: the parents as a secure base and haven, procedural memory, parents' attachment needs, implicit responding, children as miscuing.

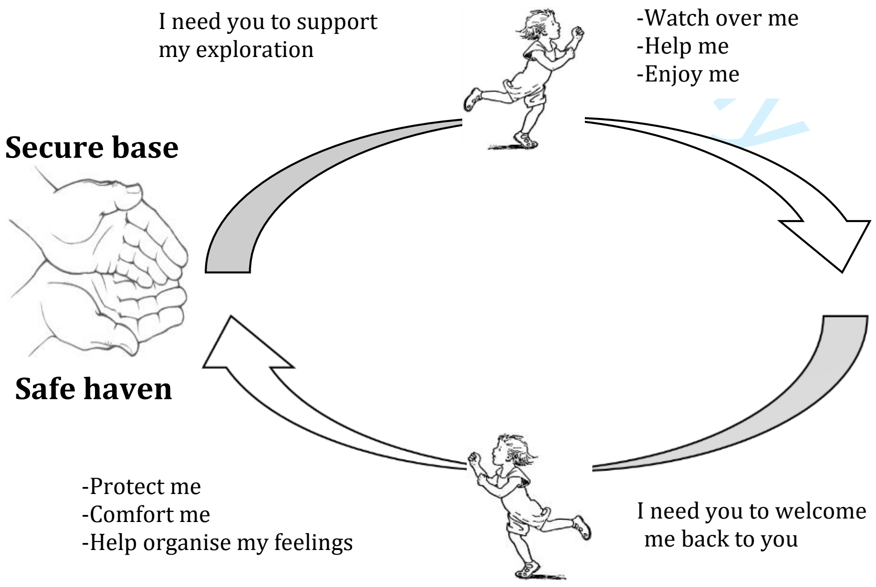

For peer review only - http://bmjopen.bmj.com/site/about/guidelines.xhtml

Externalising and Self-Autism Mapping (SAM)

Children may be invited to make ‘autism’ from PlayDoh. The parents discuss the child’s responses and elaborate with their own views of what they regard as typical as opposed to autistic and how the influence of the autism may alter in different contexts and reduce or expand as the child develops.

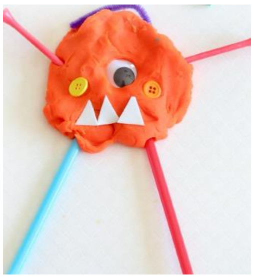

This is developed by inviting the child to engage in mapping the autism (SAM) which is initiated by introducing the child to two figures representing ‘Just me’ and “Me and autism’ Redrawing these figures can involve the child engaged in a discussion of the two figures in terms of what is, and is not affected by autism. At a later date a dotted circle is added and the children can identify aspects of themselves about which they are unsure.

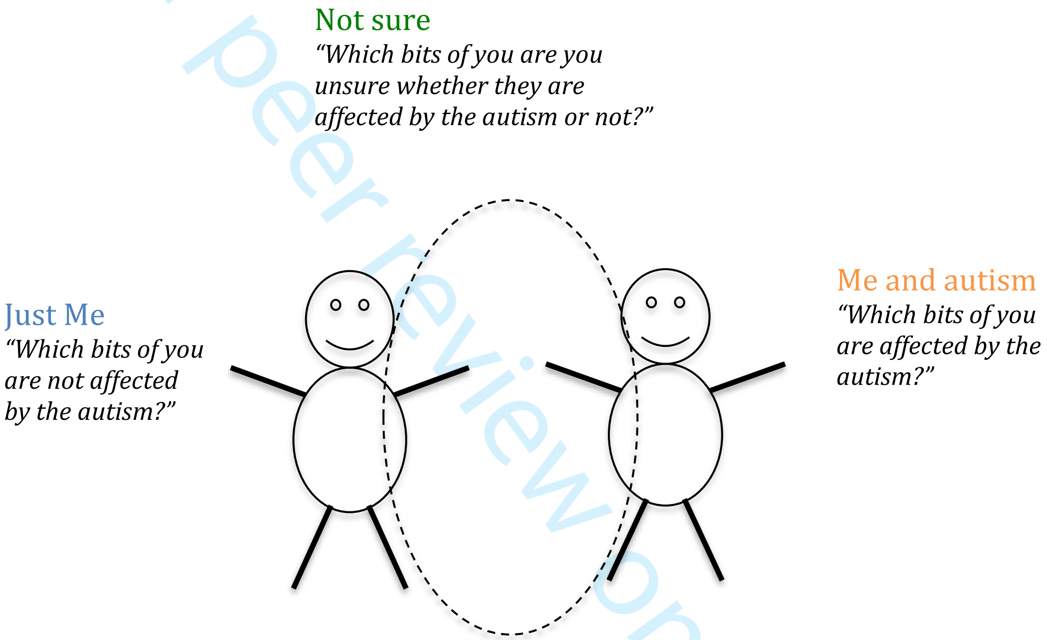

For peer review only

Area of Special Interest (ASI)

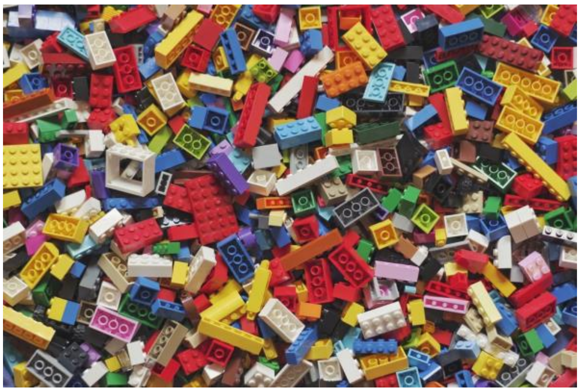

The Child with autism and the siblings are invited to bring an object or present their interest of special interest. This can take and form that the child is comfortable with. The ASI is used to raise the child’s confidence, model communication and explore relationships, perceptions and difficulties. E.g. if mum was a Lego character who would she be? Who would you be? How do the characters manage difficulties? etc.

For peer review only - http://bmjopen.bmj.com/site/about/guidelines.xhtml

Button Sculpt

Families use buttons to map and discuss relationships (how close they are to each other) before and after the diagnosis and how they predict it might be in the future. This helps to the families understand the relationships, feelings and perceptions at key times and also reflect on how these may change and develop.

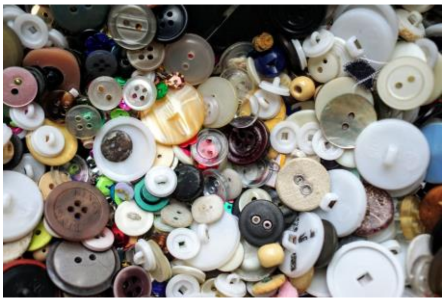

Additional activities

Role play
Often used with tracking to try out new approaches or rehearse successful interactions or strategies.

Reflective conversations
Therapists have a conversation about their thoughts on the family issues and dynamics. The family are invited to listen and then to discuss between themselves and the therapists what was of interest and relevant, what they agreed or disagreed with etc. This is an opportunity to build a sense of openness, support the therapeutic relationships, validate the family, praise successes and also to offer specific suggestions.

Drawing
Family members draw aspects of their life e.g. a typical day or a day at school. Drawing can also be used as part of SAM if the child wishes to draw autism or themselves as part of the activity.

Video feedback activities
Families watch video training materials of other families and use tracking techniques to analyse what is happening, make connections with their own experience and suggest ways forward. This is used in sessions 1 and 5 with the parents. They are asked to watch videos of a child and mum dealing with a meltdown and in the final session a young person with autism describing how he manages emotional and sensory difficulties. These activities are intended to help reflection in a non-challenging way and empower parents to act as consultants to others in similar positions.

For peer review only

Key references

Asen, E., and Scholz, M. (2009) *Multi-Family Therapy: Concepts and Techniques*, London: Routledge

Dallos, R. (2006) *Attachment Narrative Therapy: Integrating Attachment , Systemic and Narrative Therapies.* Maidenhead: Open Univ. Press/McGraw Hill

Dallos, R. and Draper, R. (2015 4th Edn) *An Introduction to Family Therapy*. Maidenhead: Open Univ. Press/McGraw Hill

Powell, B., Cooper, G., Hoffman, K. and Marvin, B. (2014). *The Circle of Security Intervention: Enhancing Attachment in Early – Child Relationships.* London: Guilford Press

CONSORT 2010 checklist of information to include when reporting a randomised trial*

Section/Topic	Item No	Checklist item	Reported on page No
**Title and abstract**			
	1a	Identification as a randomised trial in the title	1
	1b	Structured summary of trial design, methods, results, and conclusions (for specific guidance see CONSORT for abstracts)	1
**Introduction**			
Background and objectives	2a	Scientific background and explanation of rationale	3
	2b	Specific objectives or hypotheses	3
**Methods**			
Trial design	3a	Description of trial design (such as parallel, factorial) including allocation ratio	4
	3b	Important changes to methods after trial commencement (such as eligibility criteria), with reasons	4
Participants	4a	Eligibility criteria for participants	4
	4b	Settings and locations where the data were collected	4
Interventions	5	The interventions for each group with sufficient details to allow replication, including how and when they were actually administered	4,5
Outcomes	6a	Completely defined pre-specified primary and secondary outcome measures, including how and when they were assessed	5,6
	6b	Any changes to trial outcomes after the trial commenced, with reasons	None
Sample size	7a	How sample size was determined	N/A
	7b	When applicable, explanation of any interim analyses and stopping guidelines	N/A
Randomisation:			
Sequence generation	8a	Method used to generate the random allocation sequence	N/A
	8b	Type of randomisation; details of any restriction (such as blocking and block size)	5
Allocation concealment mechanism	9	Mechanism used to implement the random allocation sequence (such as sequentially numbered containers), describing any steps taken to conceal the sequence until interventions were assigned	N/A
Implementation	10	Who generated the random allocation sequence, who enrolled participants, and who assigned participants to interventions	5
Blinding	11a	If done, who was blinded after assignment to interventions (for example, participants, care providers, those	5

For peer review only

		assessing outcomes) and how	
	11b	If relevant, description of the similarity of interventions	N/A
Statistical methods	12a	Statistical methods used to compare groups for primary and secondary outcomes	6
	12b	Methods for additional analyses, such as subgroup analyses and adjusted analyses	6
**Results**			
Participant flow (a diagram is strongly recommended)	13a	For each group, the numbers of participants who were randomly assigned, received intended treatment, and were analysed for the primary outcome	See consort diagram
	13b	For each group, losses and exclusions after randomisation, together with reasons	As above
Recruitment	14a	Dates defining the periods of recruitment and follow-up	As above
	14b	Why the trial ended or was stopped	N/A
Baseline data	15	A table showing baseline demographic and clinical characteristics for each group	6
Numbers analysed	16	For each group, number of participants (denominator) included in each analysis and whether the analysis was by original assigned groups	6,7,8
Outcomes and estimation	17a	For each primary and secondary outcome, results for each group, and the estimated effect size and its precision (such as 95% confidence interval)	N/A
	17b	For binary outcomes, presentation of both absolute and relative effect sizes is recommended	N/A
Ancillary analyses	18	Results of any other analyses performed, including subgroup analyses and adjusted analyses, distinguishing pre-specified from exploratory	
Harms	19	All important harms or unintended effects in each group (for specific guidance see CONSORT for harms)	7
**Discussion**			
Limitations	20	Trial limitations, addressing sources of potential bias, imprecision, and, if relevant, multiplicity of analyses	2 and 11
Generalisability	21	Generalisability (external validity, applicability) of the trial findings	10/11
Interpretation	22	Interpretation consistent with results, balancing benefits and harms, and considering other relevant evidence	10/11
**Other information**			
Registration	23	Registration number and name of trial registry	2
Protocol	24	Where the full trial protocol can be accessed, if available	3
Funding	25	Sources of funding and other support (such as supply of drugs), role of funders	

*We strongly recommend reading this statement in conjunction with the CONSORT 2010 Explanation and Elaboration for important clarifications on all the items. If relevant, we also recommend reading CONSORT extensions for cluster randomised trials, non-inferiority and equivalence trials, non-pharmacological treatments, herbal interventions, and pragmatic trials. Additional extensions are forthcoming: for those and for up to date references relevant to this checklist, see www.consort-statement.org.

For peer review only

BMJ Open

SAFE, a new therapeutic intervention for families of children with autism: a randomised controlled feasibility trial.

Journal:	*BMJ Open*
Manuscript ID	bmjopen-2020-038411.R2
Article Type:	Original research
Date Submitted by the Author:	30-Nov-2020
Complete List of Authors:	McKenzie, Rebecca; University of Plymouth, Institute of Education<br>Dallos, Rudi; Department of Clinical Psychology, University of Plymouth<br>Stedmon, Jacqui; The University of Plymouth, Clinical Psychology<br>Hancocks, Helen; University of Plymouth, Peninsula Clinical Trials Unit<br>Vickery, Patricia; University of Plymouth, Peninsula Clinical Trials Unit<br>Barton, Andy; South West Research Design Service,<br>Vassallo, Tara; University of Plymouth, Institute of Education<br>Myhill, Craig; University of Plymouth, Institute of Education<br>Chynoweth, Jade; University of Plymouth, Medical statistics<br>Ewings, Paul; Research Design Service, Research Office
**Primary Subject Heading**:	Health services research
Secondary Subject Heading:	Paediatrics, Health services research
Keywords:	Child & adolescent psychiatry < PSYCHIATRY, MENTAL HEALTH, Community child health < PAEDIATRICS

SCHOLARONE™
Manuscripts

I, the Submitting Author has the right to grant and does grant on behalf of all authors of the Work (as defined in the below author licence), an exclusive licence and/or a non-exclusive licence for contributions from authors who are: i) UK Crown employees; ii) where BMJ has agreed a CC-BY licence shall apply, and/or iii) in accordance with the terms applicable for US Federal Government officers or employees acting as part of their official duties; on a worldwide, perpetual, irrevocable, royalty-free basis to BMJ Publishing Group Ltd ("BMJ") its licensees and where the relevant Journal is co-owned by BMJ to the co-owners of the Journal, to publish the Work in this journal and any other BMJ products and to exploit all rights, as set out in our licence.

The Submitting Author accepts and understands that any supply made under these terms is made by BMJ to the Submitting Author unless you are acting as an employee on behalf of your employer or a postgraduate student of an affiliated institution which is paying any applicable article publishing charge ("APC") for Open Access articles. Where the Submitting Author wishes to make the Work available on an Open Access basis (and intends to pay the relevant APC), the terms of reuse of such Open Access shall be governed by a Creative Commons licence – details of these licences and which Creative Commons licence will apply to this Work are set out in our licence referred to above.

Other than as permitted in any relevant BMJ Author's Self Archiving Policies, I confirm this Work has not been accepted for publication elsewhere, is not being considered for publication elsewhere and does not duplicate material already published. I confirm all authors consent to publication of this Work and authorise the granting of this licence.

For peer review only

SAFE, a new therapeutic intervention for families of children with autism: a randomised controlled feasibility trial

Rebecca McKenzie[1], Rudi Dallos[2], Jacqui Stedmon[2], Helen Hancocks[3], Patricia Jane Vickery[3], Andy Barton[4], Tara Vassallo[1], Craig Myhill[1], Jade Chynoweth[5], Paul Ewings[6]

Author affiliations

1. Institute of Education, University of Plymouth, Plymouth, UK
2. Department of Clinical Psychology, University of Plymouth, Plymouth, UK
3. Peninsula Clinical Trials Unit, University of Plymouth, Plymouth, UK
4. South West Research Design Service. Plymouth, UK
5. Medical Statistics, University of Plymouth, Plymouth, UK
6. Research Office, Research Design Service, Taunton, UK

Correspondence to: Dr Rebecca McKenzie rebecca.mckenzie@plymouth.ac.uk

Key words: Family Therapy, autism, intervention, feasibility, mental health

Word count excluding title page, abstract, references, figures and tables: 5534

ABSTRACT

Objectives: To establish the feasibility of a definitive randomised controlled trial of SAFE, an intervention for families of children with autism.

Design: A randomised, controlled, multi-centred feasibility study.

For peer review only

Setting: Participants were identified from three NHS diagnosing centres in Plymouth and Cornwall and a community pathway.

Participants: 34 families of a child with a diagnosis of autism severity level 1 or 2 between 3-16 years. Four families were lost to follow up.

Interventions: Systemic Autism-related Family Enabling (SAFE) is a manualised five-session Family Therapy-based intervention delivered over 16 weeks and designed for families of children with autism. SAFE involves families attending five 3-hour sessions led by systemic practitioners.

Primary and secondary outcome measures: The proposed primary outcome measure was the Systemic CORE 15 (SCORE-15). Proposed secondary outcome measures: Patient Health Questionnaire (PHQ)-Somatic Anxiety Depressive Symptoms, the Coding of Attachment-Related Parenting for use with children with Autism (CARP-A), the Child Behaviour Checklist (CBCL), the Reflective Functioning Questionnaire (RFQ) and the Caregiving Helplessness Questionnaire (CGHQ). Outcome measures were collected at baseline and 24 weeks post randomisation.

Results: All Primary Caregivers retained in the study completed the SCORE-15 at both time points. 34 of the target of 36 families were recruited and 88% of families were retained. Training for therapists was effective. Feedback revealed willingness to undergo randomisation. There was 100% attendance at appropriate sessions for core family members. The SCORE-15 showed reduction in scores for families receiving SAFE compared to controls suggesting positive change. Qualitative data also revealed that families found the study acceptable and families receiving SAFE experienced positive change. Feedback indicated that the SCORE-15 should be retained as a primary measure in a future trial, but secondary measures should be reduced.

Conclusions: This study indicates that a larger trial of SAFE is feasible. Findings suggest that SAFE can address current gaps in recommended care, can be confidently delivered by NHS staff and has potential as a beneficial treatment.

Trial registration number: ISCTRN83964946 IRAS 213527

Ethics The trial protocol received ethical approval from the South West-Exeter Research Ethics Committee (Ref: 17/SW/0192).

For peer review only

STRENGTHS AND LIMITATIONS OF THIS STUDY

- The study addressed a gap in the available research data, and produced important feasibility information to inform a fully powered randomised controlled trial.
- The study explored the feasibility of using measures of family function and a range of mental health measures.
- Quantitative feasibility data were complemented by qualitative focus groups and interviews.

For peer review only - http://bmjopen.bmj.com/site/about/guidelines.xhtml

- The study explored the feasibility of economic analysis measures in a population, which includes adults and their children with developmental disorders.
- The participants were recruited from two NHS Trusts in adjacent counties in the South West of England, leading to potential bias. A future randomised controlled trial will extend to centres across the UK including Scotland and Wales.

For peer review only

INTRODUCTION

For peer review only - http://bmjopen.bmj.com/site/about/guidelines.xhtml

More than 1% of the UK population has a diagnosis of autism [1]. Families of children with a diagnosis of autism present complex needs. Children with autism have impairments in social communication and restricted, repetitive behaviours and interests [2]. Autism is widely accepted to have a genetic component and the broader autism phenotype is disproportionally represented among family members [3]. Mental health problems are experienced by more than 70% of individuals with autism and more than 50% of their parents [4,5]. Parents of children with autism are more likely to be hospitalised for mental disorders than parents of typically developing children [6] and mothers of children with autism are reported to have higher unmet needs, more difficulties coping, and lower satisfaction with service interactions than mothers of children with other disabilities [7].

As these families often exhibit psychological morbidities alongside autism in the children, costs of services to treat these problems are high [8,9]. Furthermore, untreated or unresponsive mental health problems impose societal costs making it hard for parents to interact effectively with services [10], potentially worsening outcomes for children and exacerbating the substantial economic burden of autism [9]. Explanations for high levels of affective disorders in these families include: stress associated with the condition of autism, genetic factors, and intergenerational family dynamics. Parenting children with autism involves stresses associated with challenging behaviour, communicative difficulties, isolation and atypical attachment behaviour displayed by children [11]. In addition, these parents suffer from poorer quality of life, and higher levels of stress compared to other populations due to societal issues including stigma, unemployment and difficulty accessing support [12,13]. Parents of children with autism report that a consequent lack of psychological wellbeing exacerbates maladaptive behaviour in their children [14], which is likely to result in unhelpful cycles of distress and hopelessness.

Previous research demonstrates that experience of trauma and abuse among women is associated with elevated risk of autism developing in their subsequent offspring [15,16]. Hence, it is possible that mothers of children with autism are more likely than the general population to be coping with previous traumatic events. Families of children with autism can experience positive family life, cope well with difficulties and enjoy good relationships with their children [17], but they represent a high-risk group, for whom treatment is disjointed, costly and inadequate [18,19].

A more joined-up approach is required, focusing on communication within families, strategies for coping with challenging behaviour and associated problems and building on strengths and relationships to alleviate mental health difficulties. The current study should be placed in the context of international calls for improved services and care for families of children with autism at country level [20], alongside National Institute of Health and Care excellence (NICE) guidelines and recommendations [21,22], as well as developments regarding children's service provision proposed by the Munroe Report [23,24]. Families of children with autism themselves highlight the importance of professionals working therapeutically with children and the wider family [6].

For peer review only

SAFE (Systemic Autism-related Family Enabling) is a systemic family therapy approach designed to address autism-related needs including problem-solving, poor socio-communication, mental health difficulties and challenging behaviour [25]. Systemic Family Therapy is a well-recognised, evidence-based psychotherapeutic approach [26]. Despite evidence that family therapies can provide benefits to children with autism and their parents [27,28] its efficacy for addressing challenges associated with this condition has not been subject to a randomised controlled trial. In addition, existing research overwhelmingly shows that families of children with autism want interventions which make real improvements to their daily life and sense of wellbeing [29,30].

Patient and Public Involvement

This study grew from the articulated needs of over 90 families surveyed and interviewed through the Plymouth Autism Network, specifically the lack of support post-diagnosis and the need for support for the whole family. The main aim was to develop a family-orientated support package and conduct a feasibility study, definitive trial and ultimately implement an intervention to be offered to all families after diagnosis. To achieve this aim in a sustainable manner we included families as partners at every stage of design and application.

Prior to commencement of the trial a pilot study was conducted with families acting as consultants. From the pilot we recruited interested families to form the SAFE Family Consultation Group. Their representative was a co-applicant on the initial bid and was employed as a research assistant. The consultation group were consulted at every stage of the trial including the initial application, developing, refining and administering the intervention, developing trial materials, recruitment, training and dissemination.

The family representative attended all trial management and research meetings and took part in all training. She disseminated to consultation group members and where appropriate, meetings with the wider group were held. The input from the families has been invaluable in engaging participants and ensuring their wellbeing.

Patient and Public Involvement (PPI) was an intrinsic part of this study and our continued positive engagement and partnership with families of children with autism is a strength. The research emerged from the difficulties articulated by families and we worked with them to develop solutions. We feel that this way of working made a substantial impact on the outcomes for the families receiving the intervention and the fact that the feedback from families was overwhelmingly positive. We also believe that PPI ensured a more ethical and thorough study which did not lose sight of the ultimate aim. The input of families throughout provided considerable motivation for the research team in delivering the study despite the challenges faced.

For peer review only - http://bmjopen.bmj.com/site/about/guidelines.xhtml

Aims and Objectives

The aim of the study was to establish the feasibility of a definitive randomised controlled trial of SAFE.

Our objectives were to:

1. Demonstrate ability to identify, recruit and randomise eligible families
2. Verify that proposed outcome measures and follow-up were acceptable, and targets for loss to follow-up were achievable
3. Assess adherence of families to the intervention
4. Gather quantitative data on outcomes to inform the design (and sample size) of the future trial
5. Adapt existing resource use questionnaire and assess the feasibility of preference based instruments for this population to facilitate a future economic evaluation
6. As part of the feasibility of process evaluation, collect data on the families' experience of SAFE and the study itself
7. As part of the feasibility of process evaluation, ensure that proposed training arrangements were effective and scalable
8. Provide operational experience to manage the future trial

METHODS AND ANALYSIS

The study comprised a randomised, controlled, multi-centred feasibility study including families of children with a diagnosis of autism [25].

Participants

Participants were identified from three NHS diagnosing centres in Plymouth and Cornwall via a search of clinical records or recruitment directly after diagnosis. A community recruitment pathway was added after commencement of the study to alleviate the burden on clinical staff and increase participation. Families were recruited by posters in local community venues.

Eligibility was determined by diagnosing clinicians or from clinical records or diagnostic letter as well as discussion with the family. A member of the research team gained consent during the first home visit. Pilot data suggested that SAFE was most effective and accessible for children without severe symptoms or an intellectual impairment. Children with severe communication difficulties found it difficult to engage with some SAFE activities. For this feasibility study, therefore, our target population was families of children with autism severity level 1 or 2 with no intellectual impairment.

Eligibility criteria:

• Family included child with autism, aged 3-16 years
• Diagnosis of Autism Spectrum Disorder (ASD) severity level 1 or 2 as documented in DSM-5 [2]

For peer review only

• Diagnosed within 12 months of consenting to the study
• If other diagnoses were present, ASD must be primary diagnosis
• Family were willing to comply with study requirements

Exclusion criteria:
• Child with ASD severity level 3 as documented in DSM-5 [2]
• Child with ASD and intellectual impairment
• Serious concomitant illness in child or family, or other circumstances such that they were unable to comply with study requirements
• Families who may have been a risk to safety of research staff
• Insufficient English language, or capacity for parent/child to consent/assent to the study

The intervention
SAFE is a manualised intensive programme of systemic family therapy designed to treat maladaptive autistic symptoms and mental health-related difficulties encountered by families of children with autism (See supplementary file - Appendix 1). SAFE lead therapists were qualified NHS systemic practitioners who received an additional 4-day training course in SAFE principles and delivery. Each lead therapist was accompanied by a support therapist trained in SAFE. SAFE provides an array of therapeutic activities based on attachment theory, established systemic practice and the known visual processing preferences of people with autism [31,32]. SAFE is best seen as a toolkit with a variety of activities which can be applied to family therapy flexibly. Activities include visual tasks, drawing, modelling, role-play and tracking circular patterns. Sessions are led by family need and the therapists and family work collaboratively, often in a playful way, using family resources, therapist expertise and the tools that SAFE provides. SAFE draws heavily from well-documented active and playful approaches in family therapy practice and literature [33]. Each therapy session included two therapists with a minimum of intermediate family therapy level of qualification and 4 days training in SAFE principles. Between weeks 1 and 16, families allocated to the SAFE intervention attended five 3-hour SAFE therapy sessions. Sessions 1 and 5 were multi-family sessions and took place in a community setting. Sessions 2, 3 and 4 were for individual families and took place in a community venue or the family home. Following completion of the therapy programme, families attended a group follow-up session at 24 weeks post-allocation. Trained support workers from local voluntary groups attended this follow-up session and gave the families information about continued support through existing networks.

Support as Usually Employed
Families were typically offered a post-diagnosis follow-up appointment with the diagnosing paediatrician. Parents of children whose symptoms were not severe tended to be directed to local authority parenting classes. In some cases families were also directed to relevant resources, for example, The National Autism Society, Gateway ASD and the NHS Child and Adolescent Mental Health Services. For families where a member was experiencing depression or anxiety, treatment varied and was not linked to autism-related

For peer review only

care. Referral was often through the GP with patients receiving cognitive behavioural therapy as part of the improved access for psychological therapies service and or medication.

Procedure

Families were recruited in four cohorts of between 6 and 12 families (two cohorts from Plymouth and two from Cornwall). Post-baseline, once sufficient families had been recruited to establish a cohort, families were randomised 2:1 to the SAFE intervention plus Support as Usually Employed (SAFE+SUE) or Support as Usually Employed only (SUE). Randomisation was undertaken by a member of the Peninsula Clinical Trials Unit (CTU) programming team by means of a 24-hour web-based system created by the CTU in conjunction with a statistician independent from the trial team, and used random permuted blocks, with stratification by study site. Twenty two families were allocated to SAFE+SUE and 12 families were allocated to SUE (See Figure 1). Clinical staff and research staff collecting outcome data were blinded to allocation. Those in the intervention arm received a preparatory home visit from a therapist.

Outcome measure data were collected at baseline and 24 weeks post-randomisation. Additional data were collected from the intervention participants: post allocation, at each therapy session and at the feedback day (See Figure 1).

Feasibility outcome measures

- Ability to identify, recruit and randomise eligible families
- Acceptability of proposed outcome measures and follow-up schedule to participants, and whether targets for loss to follow-up were achievable
- Adherence of therapists and families to the intervention
- Ability to gather quantitative data on outcomes
- Appropriateness of resource use questionnaires and preference-based instruments for this population
- Effectiveness and scalability of training arrangements

Clinical outcome measures

The proposed primary outcome measure was, the Systemic CORE 15 (SCORE-15) [34]. This is a 15-item paper-based survey made up of three subcategories. For each item scores range from 1-5. The potential range of overall scores is 15 to 75, with a lower score indicating higher family functioning. Based on available literature [35], a meaningful change in family function, from pre to post intervention, would be solidly indicated by a reduction of 3 on overall scores. The SCORE is the primary measure of family functioning employed in Children and Young People's Improving Access to Psychological Therapies national programme, and is the gold standard for assessing the impact of family therapy on quality of life in the UK [36]. Every able family member was asked to complete the SCORE-15 at baseline and 24 weeks post-randomisation.

For peer review only

The SCORE-15 is particularly well-suited as a primary measure for this study as it is a measure for the whole family and, like SAFE, it has a systemic framework based on the premise that relational dynamics are central to the wellbeing of all family members. In addition, the three dimensions of SAFE are well matched to the needs of this population in that the SCORE-15 is sensitive to change in aspects of mental health and problem solving defined by the three sub-categories measuring: family strengths, resilience and coping (*Strengths and Adaptability*); communication and understanding within the family (*Disrupted Communication*) and changes in ability to manage problems (*Overwhelmed by Difficulties*). The SCORE also correlates with measures of parental satisfaction, mental health, strengths and difficulties and life satisfaction [37].

Scores on the proposed secondary outcome measures, which index changes in child behaviour, child-parent attachment, anxiety and depression:

- Patient Health Questionnaire (PHQ)-Somatic Anxiety Depressive Symptoms completed by parents. This comprises the PHQ-9 measuring depression and the generalised anxiety disorder, GAD-7 measuring anxiety [38].
- Adapted mutuality subscale of the Coding of Attachment-Related Parenting for use with children with Autism (CARP-A) [39] completed by the whole family. The CARP-A is a validated observational measure of a child with autism's attachment behaviour towards their carer. The measure was adapted to form a family Lego 'building your house' task.
- The Child Behaviour Checklist [40] completed by the Primary Caregiver. This is a 30-item paper-based survey, which detects emotional and behavioural problems.
- The Reflective Functioning Questionnaire [40] completed by parents, measures ability to understand own and others' mental states.
- Caregiving Helplessness Questionnaire (CGHQ) [41] completed by the parents. This is a 26-item questionnaire designed to assess aspects of disorganised caregiving.

Scores on proposed generic health economic outcome measures also provided data on the feasibility of cost-effectiveness analysis:

- EuroQol 5 dimensions, a standardised generic instrument for measuring health outcome.
- Child Health Utility 9 Dimensions, a paediatric generic preference-based measure of health-related quality of life.
- Resource Use Questionnaire (RUQ), a paper-based questionnaire completed by parent about his/her child's use of healthcare and social resources. The RUQ is designed to identify the NHS and social care resource use for the economic evaluation.

Proposed qualitative outcomes assessed via focus groups:

- Acceptability of SAFE and the trial process for participants and therapists
- Reasons for declining and withdrawing from the study

For peer review only - http://bmjopen.bmj.com/site/about/guidelines.xhtml

Additional process measures completed after each SAFE session by therapists and families allocated to SAFE:

- The Client Satisfaction Questionnaire (CSQ-8 completed by families)
- Helpful Aspects of Therapy Questionnaire - assessing what was helpful/unhelpful in each session (HAT completed by families)
- The Training Checklist and Questionnaire monitoring protocol adherence (TCQ) completed by therapists)

Quantitative analysis

Prior to analysis a detailed statistical analysis plan was created and agreed by the research team. For each outcome measure, the relevant scores were calculated and presented descriptively by trial arm. Where available, published guidelines were used to process, score and summarise the measures including the use of imputation in the event of missing items on a questionnaire. Summary measures were calculated as appropriate, for example, means and SD, medians and ranges, numbers and percentages in categories. The only analysis contrasting the two groups was an interval estimate in the form of a 95% confidence interval (CI) for the primary outcome, the SCORE-15.

Qualitative analysis

Prior to analysis a qualitative analysis plan was agreed. Focus group interviews were conducted for SAFE+SUE and SUE families from each cohort after the 24 week outcome data had been collected for that cohort. Focus groups were audio recorded and transcribed verbatim. Consequent qualitative data was managed using proprietary computer-assisted qualitative data analysis software, NVivo 10, and analysed thematically [42,43]. Summaries of process measures were compiled including quantitative data from Likert scales and thematic analysis of open-ended questions. Subsequently, independent analysis of qualitative data was conducted, including notes and memos, on a subset of the data by two of the authors and consensus reached on overarching themes relating to family experience of SAFE.

Place Figure 1. here Participant Flow Diagram (CONSORT)

RESULTS

Recruitment and randomisation

Of 138 families screened, 106 were eligible and 32 ineligible. Forty-five eligible families expressed interest of which 34 were recruited (Figure 1). Since randomisation only occurred once a complete cohort (between 6 and 12 families at each site) was recruited, the time between consent (when baseline questionnaires were completed) and randomisation varied. Data on relevant dates were available for 33 families, for whom there

For peer review only - http://bmjopen.bmj.com/site/about/guidelines.xhtml

was a median (range) of 21 days (2 to 111) between baseline questionnaire completion and randomisation, with a mean (SD) of 34 (32) days. Nineteen families completed the 24-week assessments for which dates were available. Six of the 19 dates were less than 22 weeks from randomisation due to time constraints, none were more than 26 weeks. All Primary Caregivers were mothers and there were 3 single parent families (2 allocated to SAFE+SUE and 1 allocated to SUE). Baseline demographics were similar for children with autism in both groups (See Table 1). Baseline constitution of families varied and are shown in Table 2.

Table 1. Summary statistics of baseline and demographic characteristics for children with autism

	SAFE + SUE (n=22)	SUE (n=12)	Total (n=34)
Age			
Mean (SD)	9.5 (2.7)	9.4 (2.4)	9.5 (2.5)
Median (range)	*10 (5-14)*	*9 (6-13)*	*10 (5-14)*
Gender n (%)			
Male	17 (77)	9 (75)	26 (76)
Female	5 (22)	3 (25)	8 (24)
Ethnicity n (%)			
White	22 (100)	12 (100)	34 (100)

Table 2. Constitution of families at baseline

Family members in addition to child with autism	No. families SAFE+SUE	No. families SUE	No. families total
Mother (only)	2	1	3
Mother + 1 sibling	2	0	2
Mother + 2 siblings	1	0	1
Mother + 3 siblings	2	0	2
Mother + 4 siblings	2	0	2
Mother + Father	0	4	4
Mother + Father + 1 sibling	6	4	10
Mother + Father + 2 siblings	3	1	4
Mother + Father + 3 siblings	1	1	2
Mother + Father + 4 siblings	0	1	1
Mother + Father + Grandmother + Grandfather	1	0	1
Mother + Father + Grandma + Grandpa + 1 sib	1	0	1

For peer review only - http://bmjopen.bmj.com/site/about/guidelines.xhtml

Mother + 3 siblings + 1 nephew	1	0	1

Acceptability of outcome measures and follow-up schedule and loss to follow-up

One SUE family was lost to follow-up and three SAFE+SUE families were lost to follow up (Figure 1). Mechanisms were in place to report and record serious adverse events (SAEs) related to mental health from consent until participants completed the follow-up or withdrew from the study. No SAEs were reported. For the purposes of the study the core family was taken to be the Primary Caregiver (all mothers) and children with autism. In one case parents were joint Primary Caregivers. Families chose who else would attend sessions and complete data. At baseline, 34 families and 122 family members provided data. At the 24-week assessment, 30 families and 97 family members provided data. Different outcomes are completed by different combinations of family members. The Primary Caregiver provided data on all measures at baseline, and 22 of the 23 other caregivers (fathers) did likewise. There was a small number of caregivers who did not provide data at 24 weeks for some questionnaires. The completion of the SCORE by the children with autism and siblings was optional. Reasons for non-completion by the child with autism were: child was too young (n=4), the visit could not be arranged (n=1), the child did not want to (n=1) and reason unknown (n=1) (see Figure 1).

Adherence of families to the intervention

Full engagement with the SAFE intervention involved five 3-hour therapy sessions. The first and last of which were multi-family sessions for the parents (children could attend if they wished) between weeks 1 and 16, with a final feedback day at 22 weeks. The Primary Caregiver was present for these sessions, except for one mother who was absent for session 1, but the father attended. The child with autism was always present at the individual sessions 2-4. Five children were also present at parent sessions 1 and 5.

Quantitative data on outcomes

Results for the SCORE-15, including expressions of uncertainty (95% CI) for estimates by randomised groups are shown in Table 3. In general there is no specific guidance available on how to handle missing data for the outcome measures used. In what follows, a decision was made to use an individual's total score data if there was no more than one missing item, for which the mean of the other items was imputed. If there were totals for subgroups, these were included if there was no more than one missing item within each subgroup, for which the mean of the other items within the subgroup were imputed.

Table 3. Values for SCORE-15, based on the "total" computed for each dimension and overall for each person. (Reduction in values and positive differences in 95% confidence intervals indicate positive change)

	Baseline	24 weeks post randomisation	Difference^

For peer review only - http://bmjopen.bmj.com/site/about/guidelines.xhtml

	SAFE+SUE		SUE		SAFE+SUE		SUE				
	Mean	SD	Mean	SD	Mean	SD	Mean	SD	95% CI		N
	Strengths and adaptability										
CWA	11.4	3.3	12.8	3.9	12.1	4.0	12.6	4.8	-3.81	3.29	21
Primary CG	10.0	2.7	9.4	2.3	10.9	3.1	10.6	2.3	-1.96	1.92	30
Other*	11.0	3.6	10.2	3.0	11.1	3.9	11.2	4.6	-1.59	3.87	43
Whole family*	10.8	3.3	10.5	3.2	11.2	3.7	11.3	3.9	-0.89	2.07	94
	Overwhelmed by difficulties										
CWA	12.3	3.9	12.3	5.5	13.5	4.2	14.1	5.5	-2.89	5.58	21
Primary CG	12.4	3.5	12.1	4.0	11.5	2.7	12.7	4.1	-0.69	3.79	30
Other*	12.1	3.9	12.2	3.9	11.7	3.2	13.1	4.2	-0.63	3.86	43
Whole family*	12.2	3.7	12.2	4.2	12.1	3.4	13.2	4.4	-0.03	2.96	94
	Disrupted communication										
CWA	12.8	4.3	11.8	4.0	13.2	4.4	11.4	2.9	-5.02	3.08	21
Primary CG	11.9	4.8	9.6	2.8	10.2	2.6	9.6	2.5	-1.95	2.04	30
Other*	12.9	4.6	12.6	4.2	12.3	3.4	11.2	3.6	-2.91	1.59	43
Whole family*	12.6	4.5	11.5	3.9	11.9	3.6	10.7	3.1	-2.03	0.92	94
	Overall Score										
CWA	36.4	10.1	36.8	11.9	38.8	11.3	38.1	12.0	-9.68	11.22	21
Primary CG	34.3	9.7	31.1	7.6	32.5	7.2	33.0	7.9	-3.49	7.35	30
Other*	36.1	10.2	34.9	9.5	35.1	7.5	35.5	10.4	-3.81	7.24	43
Whole family*	35.7	9.7	34.1	9.5	35.2	8.6	35.2	9.8	-2.09	5.26	94

* scores averaged across all relevant family members

^ difference between SUE and SAFE + SUE at 24-week follow up, adjusting for baseline in ANCOVA; based on individuals with complete data at both baseline and 24-week assessment; no allowance for hierarchy of data

CG = Caregiver
CWA = Child with a diagnosis of autism

For peer review only

Feasibility of using economic evaluation instruments for this population

Completeness of health economics data was achieved in intervention and control groups for the primary care based, community based and mental health based services at 100% response rate at baseline dropping to 82% and 83% at 24 weeks in intervention and control groups, respectively. The outpatient based services had response rates of 68% and 67% at baseline, but completeness improved to 82% and 83% in intervention and control groups respectively at 24 weeks.

For EQ-5D-5L, at baseline the control group had a 100% response rate. At 24 weeks the response rates were 82% and 83% of participants in intervention and control groups, respectively.

As expected CHU-9D responses were largely provided by children with autism (completed by the Primary Caregiver where necessary). Response rate in the intervention group fell from 77% to 73% for children with autism but remained constant with three (14%) Primary Caregiver responses at baseline and 24 weeks. In the control group, response rate fell from 83% to 58% in children with autism and 25% to 17% in Primary

For peer review only - http://bmjopen.bmj.com/site/about/guidelines.xhtml

Caregivers (mothers) from baseline to week 24. Despite indications for increased appropriate service use coupled with reduced cost over time for SAFE+SUE children with autism, the economic evaluation focused only on children with autism, consequently insufficient data was collected for whole family service use and costs.

The families' experience of the study and the SAFE intervention

Qualitative data on the experience of the study were collected from SAFE+SUE and SUE families in family feedback days for each cohort. Key areas revealed by analysis of the data were a) the study was not burdensome, b) that they took part to help others, c) research staff made completing tasks easier and sessions could be organised differently.

The families did not find the completion of CARP-A Lego task and questionnaires onerous:

> *"didn't mind so much doing like the Lego task I mean like it's something that we like to do anyway … but um the questionnaires didn't have a problem with those either"*

Families felt that there were too many questionnaires, but the approachable nature of the research staff made the task easier:

> *"it was all fine … it was A [Research Assistant] that came in did it and she's so friendly and approachable that it made it easy…"*

Families generally took part in the study for altruistic reasons. They wanted to be part of a study which could potentially increase support post-diagnosis. Feedback from SUE families suggested that this helped them to accept their role in the study:

> *" I thought actually this could be a way of helping future parents of diagnosed children to get the support and advice that wasn't there when I got the diagnosis"*

The focus group data also revealed that families felt sessions at home were too distracting, the sessions should be shorter, but more of them and more activities for younger children were needed.

Where families were allocated to the intervention, data on family experience of SAFE were collected via process evaluation measures, the Helpful Aspects of Therapy Questionnaire (HAT) and the Client Satisfaction Questionnaire (CSQ) completed by family members after each therapy session and via the family feedback day. Quantitative data from the HAT indicated that SAFE families found the intervention helpful with mean scores for all sessions on the HAT being rated by children and adults as helpful or very helpful. Analysis of all qualitative data from process measures and the feedback day resulted in the following

For peer review only - http://bmjopen.bmj.com/site/about/guidelines.xhtml

overarching themes: therapist helping reflection, increased understanding, feeling closer, feeling more confident, more able to reflect and problem solve, improved communication and feeling less isolated.

The multi-family sessions made families feel less alone, more able to talk about difficult issues and less judged.

> *"Talking to other parents and feeling listened to and supported"*

> *"Talking about daily issues and sharing these with the other parents, generally feeling that I am not alone"*

Families reported being able to step back and reflect, leading to more confidence in tackling family problems.

> *"Breaking down problem events and looking at causes, outcomes, choices made. Compared a recent to past events and saw progress and maturity in decision making and actions"*

SAFE therapists were seen as allies and the activities helped with family problems leading to increased understanding and improved mental health:

> "*Actually being able to hear that I'm doing OK and actually not as bad as what I thought I was, was nice. It was something that helped me with my mental and emotional state"*

Effectiveness and scalability of training

The therapists completed the Training Checklist Questionnaire (TCQ) after each session informing the potential for future scalability and establishing that training methods were effective. The TCQ was a short questionnaire documenting adherence to planned activities in each session, confidence and ease of delivering activities, rating of the effectiveness of activities and any additional resources required. Focus groups and interviews were also conducted with the therapists after completion of the intervention. The therapists felt that SAFE was an inspirational and effective intervention, was non-pathologising and child centred. The training was motivational and thorough. In 82% of sessions lead therapists reported feeling confident, that the sessions were effective and the activities were easy to deliver. The support therapists, who were less experienced, felt confident, that sessions were effective and activities easy to deliver in 63% of sessions. The therapists felt supervision was very helpful. They highlighted that the gap between training and intervention was too long.

Operational experience moving forward.

The Chief Investigator (CI) kept the study on time and within budget despite multiple challenges. The support and advice of the Trial Steering Committee (TSC), the PenCTU and the research team facilitated the development of the skill set of the CI in preparation for a definitive trial.

For peer review only - http://bmjopen.bmj.com/site/about/guidelines.xhtml

DISCUSSION

To our knowledge, this study is unique in evaluating the feasibility and efficacy of a Family Therapy-based intervention designed specifically for families of children with a diagnosis of autism. The results indicate that a larger trial of SAFE would be feasible. The findings also suggest that offering SAFE for families post-diagnosis is both feasible and potentially efficacious.

Progression to a definitive trial was supported based on pre-determined criteria met in the following ways: a) 34 of the target of 36 families were recruited. Family feedback revealed willingness to undergo randomisation as families felt the study had potential to extend services. b) 88% of families were retained. c) There was 100% attendance at appropriate sessions for core family members. d) For the SCORE-15, complete data were available at both time points and for every dimension for 88% Primary Caregivers, 62% children with autism and 55% other family members. All Primary Caregivers retained in the study completed the SCORE-15 at both time points. e) Collecting health and social care resource utilisation data and health-related quality of life data for children with autism was feasible. f) Process evaluation and feedback day showed that in 82% of sessions lead therapists felt confident, that sessions were effective and easy to deliver. g) The qualitative and process data from families indicates tentative potential for efficacy as SAFE was rated as helpful or very helpful in addressing problems. Qualitative analysis revealed the following themes of positive change: therapist helping reflection, increased understanding, feeling closer, feeling more confident, more able to reflect and problem solve, improved communication and feeling less isolated. A common thread across all qualitative data was increased mental wellbeing among family members receiving SAFE. Although we acknowledge that the current study was not powered to detect a difference, the SCORE-15 showed marked reduction in scores for SAFE+SUE suggesting positive change. A reduction of this magnitude indicates tentative potential proof of efficacy [34,35] (See Figure 2).

Place Figure 2. here

Additional analysis of the SCORE-15 data revealed that the most marked indications of positive change were experienced by Primary Caregivers with high anxiety as measured by the GAD-7 scale. The relationship between anxiety scores and values on the SCORE-15 is important as SAFE seeks to alleviate mental health issues. An indication that a reduction in anxiety and improved family function can be captured by our proposed measures and that data suggests positive change for those participants with acute difficulties receiving the intervention is therefore promising (See Figure 3).

Place Figure 3. here

For peer review only - http://bmjopen.bmj.com/site/about/guidelines.xhtml

The sample size calculation for a subsequent fully powered randomised controlled trial is based on the SCORE-15 for Primary Caregivers, which will be the primary outcome in the full trial. The minimal clinically important difference of 3 is based on the literature [35] and an SD of 8 is based on an SD of 7.3 obtained from the feasibility study. To account for a possible group effect in the intervention group we will use a conservative 0.1 for the intra-group correlation and an assumed mean group size of 6 with variance 2. Employing the conventional two-sided 5% alpha and 90% power, and retaining the 2:1 allocation, gives a requirement for 405 evaluable participants. Using the drop-out experienced in the feasibility study would increase the requirement to 460.

The feasibility study alongside previous research [4,5,6,7] strongly suggests that these families are an at-risk group who experience very limited support post diagnosis. One of the primary aims will be to carry out a definitive trial in order to address international [20] and national recommended good practice in relation to children with autism and their families [NICE guidelines 21,22]. These recommend psychosocial interventions, which improve sensitivity, responsiveness and communication within families. They also call for interventions which prevent challenging behaviour by creating care packages which address co-existing mental health problems, and provide support for families. NICE guidelines associated with child mental health also ask for the provision of psychological therapies including family therapy; the need for parents' psychiatric problems to be treated, for children's mental health to improve; and management of developmental conditions to be in parallel with mental health interventions.

NICE research recommendations associated with autism call for randomised controlled trials exploring family interventions that are designed to reduce challenging behaviour, alleviate stress and improve quality of life [21]. Our long-term goals are also informed by the Munroe Report [44], which called for children's services to offer family-centred, therapeutic intervention rather than risk management. Additionally, the current implementation of the Improved Access to Psychological Therapies scheme for children [45] promotes proactive attachment-based and family therapy approaches. SAFE has the potential to address this gap by providing a family-based intervention which is sustainable, extends existing services and is beneficial to families.

LIMITATIONS

Use of the primary measure the SCORE-15 was limited for children below 7-years and an adapted more visual version will be developed so children below 7-years can contribute. The economic evaluation focused solely on the child with a diagnosis. To ensure sufficient data is collected, a subsequent trial should include longer-term service use and costs for all family members. Families felt there were too many measures and repetition. For any subsequent trial the outcome measures would be reduced and adapted. The Reflective

For peer review only

Functioning Questionnaire (RFQ) replicated information from other measures and the Child Behaviour Checklist (CBCL) was lengthy and complex. These measures would not be used in a subsequent trial. We propose the SCORE-15 (measuring perceived change in family functioning) be retained as our primary outcome measure for Primary Caregiver alongside secondary measures being the adapted SCORE-15 (for other family members including the youngest children), PHQ-SADS (anxiety scale), CGHQ (measuring change in caregiver helplessness) and the CARP-A (measuring mutuality between family members).

Recruitment and organisation of SAFE sessions was challenging. There was large variability in the timescales between baseline and start of intervention. In any subsequent trial waiting could be managed by carrying out baseline measurement once enough families have consented for randomisation to occur. Therapists and families preferred shorter sessions in community venues, predetermined shorter appointments are proposed for any subsequent trial. Challenges anticipated in recruiting for the definitive main trial will be addressed by using a community pathway for recruitment alongside using clinical nurses to aid recruitment in clinical settings. In the current study the community pathway was introduced later in the study and did boost recruitment. We will also encourage recruitment and clarity for families by developing a recruitment open day, screening tool and appointment system for SAFE sessions based on a clinic model influenced by potential benefits of family-centred service delivery [46]. In the current study families of children with autism were not eligible if their children had intellectual disability or needed substantial support. Subsequent refinements of the intervention should address this issue to broaden the application of the intervention. Due to the lack of ethnic diversity in Plymouth and Cornwall our participants were all white. This will need to be addressed in any future trial. Given that prevalence differs between ethnic groups [47,48,49] and beliefs and support networks may also differ, it is essential for appropriate implementation across the UK, and internationally, that any future trial reflects ethnic diversity. Any definitive trial will, therefore, include centres which have an ethnically diverse client base.

CONCLUSIONS

To our knowledge this is the first study of Family Therapy as an intervention for families of children with a diagnosis of autism. The proposed primary outcome measure, the SCORE-15, indicated potential benefits for the intervention group compared to controls in coping with problems, developing strengths and communicating within families. Qualitative data showed that family members felt the intervention improved understanding, communication, confidence, mental health, reflection, problem solving and sense of isolation. Attendance and completion of outcome measures was acceptable and NHS therapists were effectively skilled after receiving brief SAFE training. This study suggests that SAFE can address current gaps in recommended care post-diagnosis and can and should be subject to a definitive trial. The findings indicate that SAFE can be effectively and confidently delivered by existing NHS staff and has potential to be a feasible and beneficial treatment for these families.

For peer review only - http://bmjopen.bmj.com/site/about/guidelines.xhtml

For peer review only

Acknowledgements: The authors would like to thank the SAFE Family Consultation Group for their input and expertise and all of the participating families from whom we learnt so much. The authors would like to thank James Cook, Mary Hosken, Dr Ben Whalley, Dr Antonieta Medina-Lara and Professor Julian Archer for their help in developing and conducting this study. The authors would also like to give special recognition to the dedication, hard work and sensitivity of Helen Hancocks.

Contributors: RM, PJV and HH were responsible for the overall development of the study and trial management. RM, HH, RD, CM, PE, AB, TV and the Family Consultation Group were involved in the conception, administration and conduct of the study. PE, JC and AB provided methodological expertise and advice on quantitative analysis, PE and JC provided statistical expertise and analysis. RD was the lead researcher, with the support of JS, on design of the intervention, supervision of therapists and qualitative analysis. TV, with the support of the Family Consultation Group, and CM advised on design, ethics and data collection particularly from the participant perspective. All authors made substantial contributions to drafting, revision and approval of this document.

Funding: This work was supported by the National Institute for Health Research (NIHR), grant number: PB-PG-0815-20058, and by Autistica, grant number: 7239

Competing interests: Contributors are co-applicants or employed research staff on the SAFE project, which receives funds from both NIHR and Autistica. Professor Rudi Dallos holds joint Intellectual Property rights for the SAFE intervention with the University of Plymouth.

Data sharing statement: Individual participant data that underlie the results reported in this article will be available on request from the lead author and Peninsula Clinical Trials Unit (PenCTU) after deidentification (text, tables, figures and appendices). In addition, the study protocol will be available on request. The data will be available from 9 months until 36 months following publication of the article. Access will be given to investigators whose proposed use of the data has been approved by an independent learned intermediary identified for this purpose. Proposals may be submitted up to 36 months following article publication. For information regarding submitting proposals and accessing data please contact the lead author rebecca.mckenzie@plymouth.ac.uk and the PenCTU penctu@plymouth.ac.uk

REFERENCES

1. National Autistic Society, *Autism Facts and History*, 2018
2. *American Psychiatric Association. Diagnostic and Statistical Manual of Mental Disorders,* 5th edn. Washington, DC: American Psychiatric Association, 2013.
3. Sasson NJ, Lam KSL, Parlier M, et al. Autism and the broad autism phenotype: familial patterns and intergenerational transmission. *J Neurodev Disord* 2013;5:11-18.

For peer review only - http://bmjopen.bmj.com/site/about/guidelines.xhtml

4. Dykens EM, Fisher MH, Taylor JL, et al. Reducing Distress in Mothers of Children with Autism and Other Disabilities: A Randomized Trial. *Pediatrics* 2014; 2013-3164.
5. Hallett V, Ronald A, Colvert, E, et al. Exploring anxiety symptoms in a large-scale twin study of children with autism spectrum disorders, their co-twins and controls. *J Child Psychol Psychiatry* 2013;54:1176-1185.
6. Daniels JL, Forssen U, Hultman M, et al. Parental Psychiatric Disorders Associated With Autism Spectrum Disorders in the Offspring. *Pediatrics* 2008;121:1357-1162.
7. Siklos S, Kerns KA. Assessing Need for Social Support in Parents of Children with Autism and Down Syndrome. *J Autism Dev Disord* 2008;36:921-933.
8. McCrone P, Dhanasiri S, Patel A, et al. *The cost of mental health care in England to 2026*. Executive Summary, London: The King's Fund. 2008
9. Buescher AVS, Cidav Z, Knapp M, et al. Costs of Autism Spectrum Disorders in the United Kingdom and the United States. *JAMA Pediatr* 2014;e1-e8.
10. Osborne LA, McHugh L, Saunders J, et al. Parenting Stress Reduces the Effectiveness of Early Teaching Interventions for Autistic Spectrum Disorders. *J Autism Dev Disord* 2008;38:1092-1103.
11. Pisula E. *Parenting Stress in Mothers and Fathers of Children with Autism Spectrum Disorders,* A Comprehensive Book on Autism Spectrum Disorders, Dr. Mohammad-Reza Mohammadi (Ed.) 2011. Available from: http://www.intechopen.com/books/a-comprehensive- bookon- autism-spectrum-disorders/parenting-stress-in-mothers-and-fathers-of-children-with-autismspectrum-disorders
12. Vasilopoulou E, Nisbet J. The quality of life of parents of children with autism spectrum disorder: A systematic review. *Res Autism Spectr Disord* 2016;23:36-49.
13. Ludlow A, Skelly C, Rohleder P. Challenges faced by parents of children diagnosed with autism spectrum disorder. *J. Health Psychol* 2012;17(5):702-11.
14. Nikmat AW, Ahmad M, Oon NL, et al. Stress and psychological wellbeing among parents of children with autism spectrum disorder. *ASEAN J of Psychiatr* 2008;9:65-72.
15. Roberts AL, Koenen KC, Lyall K, Ascherio A, Weisskopf MG. Women's posttraumatic stress symptoms and autism spectrum disorder in their children. *Res Autism Spectr Disord* 2014;8(6):608-616.
16. Roberts AL, Lyall K, Rich-Edwards JW, Ascherio A, Weisskopf MG. Maternal exposure to intimate partner abuse before birth is associated with autism spectrum disorder in offspring. *Autism* 2016;20(1):26-36.
17. Markoulakis R, Fletcher P, Bryden P. Seeing the glass half full: benefits to the lived experiences of female primary caregivers of children with autism. *Clin Nurse Spec* 2012;26:48–56.
18. Murphy M, Bolton PF, Pickles A, et al. Personality traits of the relatives of autistic probands. *Psychol Med* 2000;30:1411-1424.
19. Blackledge JT, Hayes SC. Using Acceptance and Commitment Training in the Support of Parents of Children Diagnosed with Autism. *Child Fam Behav Ther* 2006:28:1-18.
20. World Health Organization. WHA67. 8 Comprehensive and coordinated efforts for the management of autism spectrum disorders 2014.
21. NICE guidelines [CG170] https://www.nice.org.uk/guidance/cg170/chapter/patient-centred-care
22. NICE guidelines [CG128] https://www.nice.org.uk/guidance/cg128
23. Monroe E. *The Munroe Review of Child protection: Final report,* Dept. of Education, HM Stationary Office, UK 2011.
24. Goodman S, Trowler I. *Social work re-claimed: Innovative frameworks for child and social work Practice*. London: Jessica Kingsley 2012.

For peer review only

25. McKenzie R, Dallos R, Stedmon J, Hancocks H, Vickery PJ, Ewings P, Barton A, Vassallo T, Myhill C. SAFE, a new therapeutic intervention for families of children with autism: study protocol for a feasibility randomised controlled trial. *BMJ Open* 2019:9:5:e025006.
26. Stratton P. Report On The Evidence Base Of Systemic Family Therapy. *Association for Family Therapy* 2005.
27. Monteiro M. *Family Therapy and the Autism Spectrum: Autism Conversations in Narrative Practice*. London: Routledge 2016.
28. Estrada AU, Pinsof WF. The effectiveness of family therapies for selected behavioural disorders of childhood. *J Marital Fam Ther* 1995:21: 403-424.
29. Pellicano E, Dinsmore A, Charman T. What should autism research focus upon? Community views and priorities from the United Kingdom. *Autism, Special Issue Article* 2014:1-15.
30. Wallace S, Parr J, Hardy, A. *One in a hundred: Putting families at the heart of autism research*. London: Autistica 2013.
31. Buron KD, Wolfberg P. *Learners on the autism spectrum: Preparing highly qualified educators*. Shawnee: AAPC Publishing, 2008:114–37.
32. Grandin T, Scariano MM. *Emergence: Labeled autistic*. New York, NY: Warner Books, 1986.
33. Dallos R, Draper R. *An introduction to family therapy: Systemic theory and practice*: McGraw-Hill Education, 2015.
34. Stratton P, Bland J, Janes E, *et al*. Developing an indicator of family function and a practicable outcome measure for systemic family and couple therapy: the SCORE. *J Fam Ther* 2010;32:232–58.
35. Carr A, Stratton P. The score family assessment questionnaire: A decade of progress. *Fam. Process* 2017;56(2):285-301.
36. SHIFT Self-Harm Intervention, Family Therapy: a randomised controlled trial of family therapy vs. treatment as usual for young people seen after second or subsequent episodes of self harm. Protocol Grant Number: 470772 HTA Reference Number: 07/33/01. 2015.
37. Stratton P, Lask J, Bland J, Nowotny E, Evans C, Singh R, Janes E, Peppiatt A. Detecting therapeutic improvement early in therapy: validation of the SCORE-15 index of family functioning and change. *J Fam Ther* 2014; 36:1:3-19.
38. Spitzer RL, Kroenke K, Williams JBW. Validation and utility of a self report version of PRIME-MD: the PHQ Primary Care Study. *JAMA* 1999;282:1737–44.
39. Blazey L. *Coding of Attachment-Related Parenting for use with children with Autism (CARP-A)*. Manchester: Manchester University, 2007.
40. Moss E, Bureau JF, Cyr C, *et al*. Correlates of attachment at age 3: construct validity of the preschool attachment classification system. *Dev Psychol* 2004;40:323–34.
41. Solomon J, George C, eds. *Disorganised Attachment and Caregiving*. Guilford: New York, 2011.
42. Braun V, Clarke V. Using thematic analysis in psychology. *Qual Res Psychol* 2006;3:77–101.
43. Green J, Thorogood N. *Qualitative Health Methods for health Research*. London: Sage Publications, 2004.
44. Munroe E. *The Munroe Review of Child protection: Final report,* Dept. of Education, HM Stationary Office, UK 2011.
45. Department of Health. *IAPT Implementation Plan: National Guidelines for Regional Delivery*. 2008. Available at www.iapt.nhs.uk
46. Trivette CM, Dunst CJ, Hamby DW. Influences of family-systems intervention practices on parent-child interactions and child development. Topics in early childhood special education. 2010 May;30(1):3-19.
47. Keen DV, Reid FD, Arnone D. Autism, ethnicity and maternal immigration. *BJPsych* 2010;196:4:274-81.

For peer review only - http://bmjopen.bmj.com/site/about/guidelines.xhtml

48. Mandell DS, Wiggins LD, Carpenter LA, Daniels J, DiGuiseppi C, Durkin MS, Giarelli E, Morrier MJ,. Nicholas JS, Pinto-Martin JA, Shattuck PT. Racial/ethnic disparities in the identification of children with autism spectrum disorders *Am J Public Health* 2009;99:3:493-8.
49. Barnevik–Olsson M, Gillberg C, Fernell E. Prevalence of autism in children born to Somali parents living in Sweden: a brief report *Dev Med Child Neurol* 2008;50:8:598-601.

Figure Captions

Figure 1. Participant Flow Diagram (CONSORT).

Figure 2. Mean change in SCORE-15 subcategories for SAFE+SUE (SAFE) and SUE only (Control) for all family members, including children with autism, who completed the SCORE-15 at both baseline and at 24 weeks.

Figure 3. SCORE-15 total mean change for Caregivers with low and high anxiety (a reduction = positive change; high anxiety > 7 on GAD-7).

For peer review only

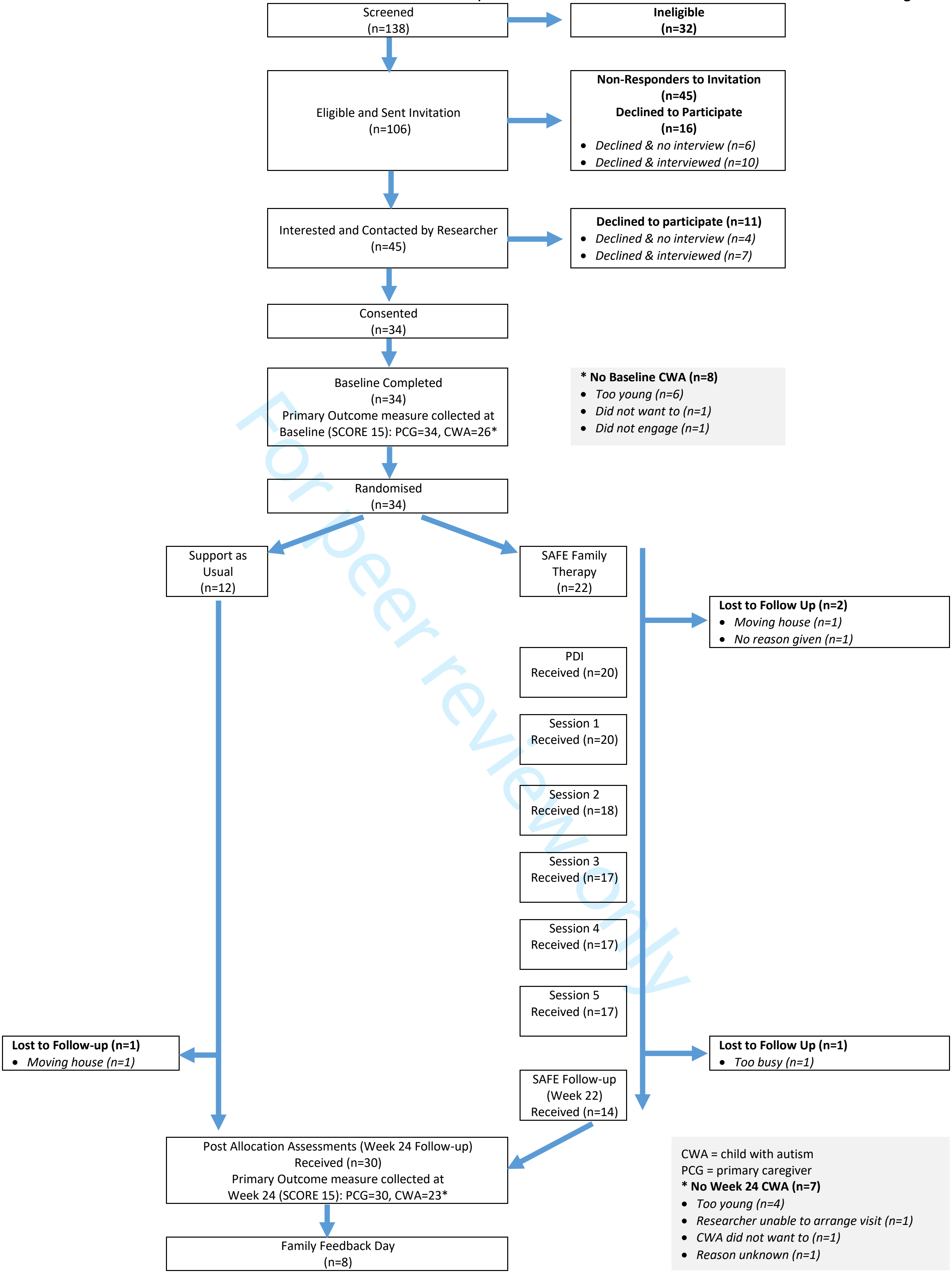

Figure 1. Participant Flow Diagram (CONSORT)

For peer review only

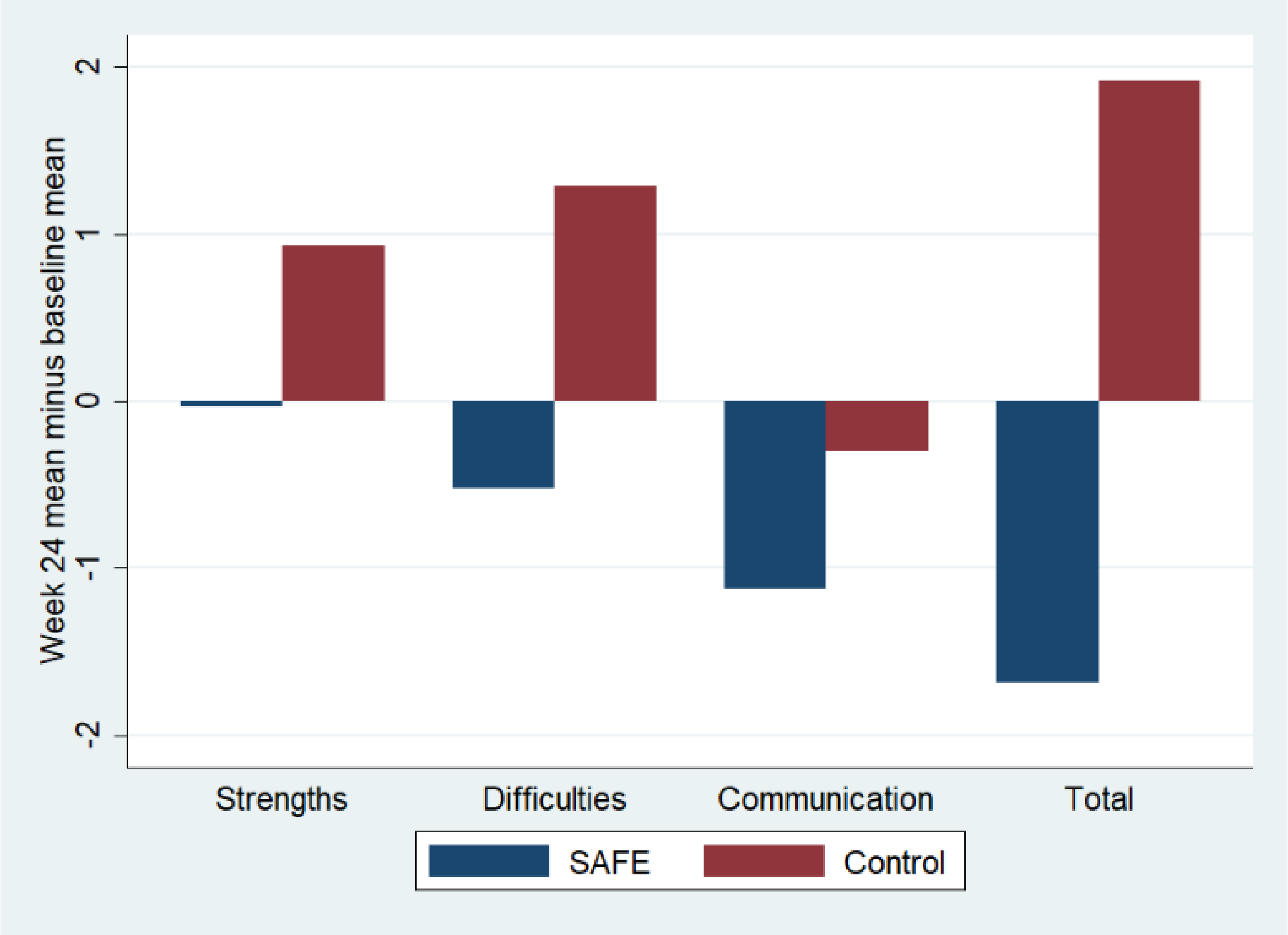

Figure 2. Mean change in SCORE-15 subcategories for SAFE+SUE (SAFE) and SUE only (Control) for all family members, including children with autism, who completed the SCORE-15 at both baseline and at 24 weeks.

For peer review only

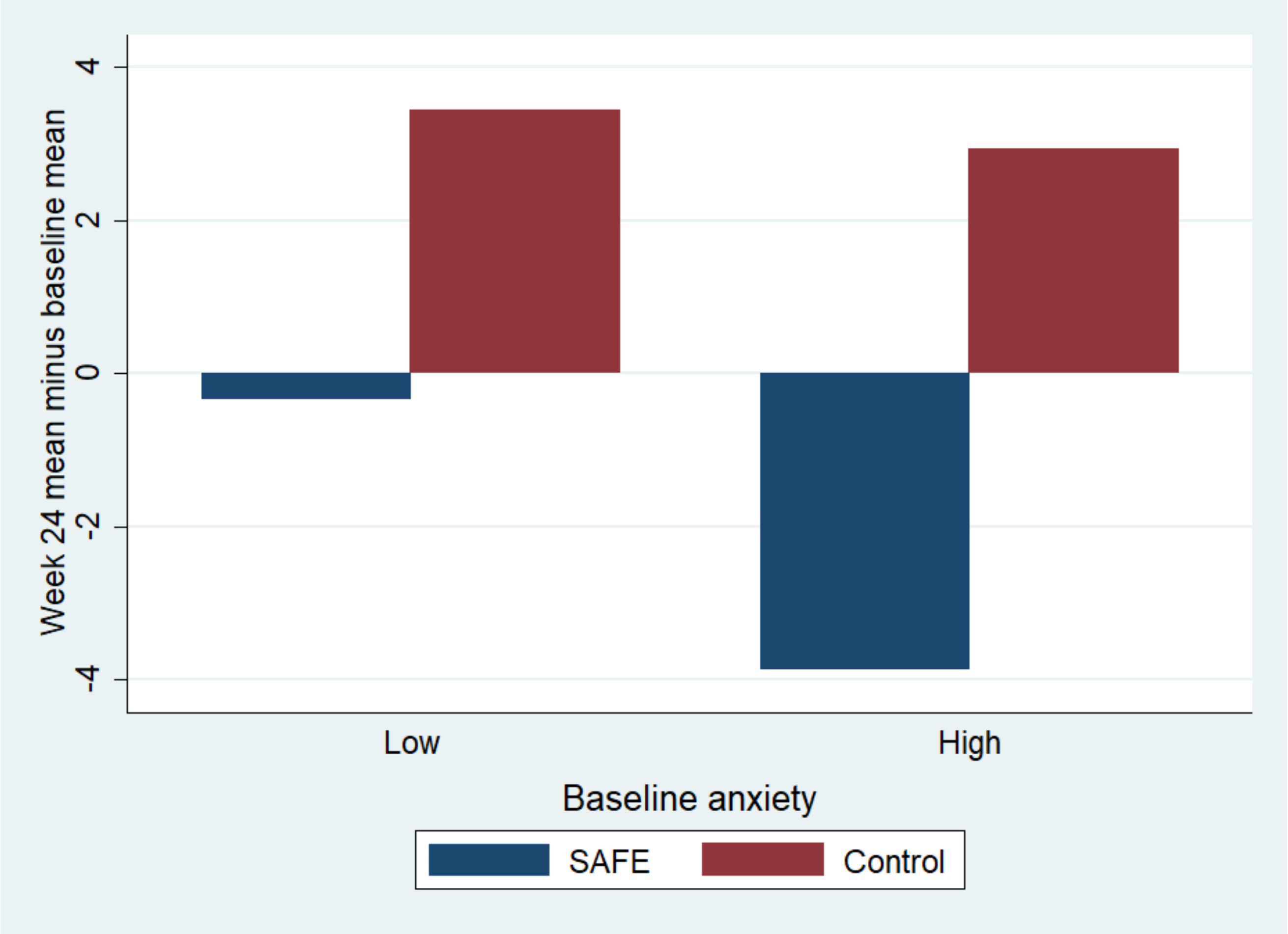

Figure 3. SCORE-15 total mean change for Caregivers with low and high anxiety (a reduction = positive change; high anxiety > 7 on GAD-7).

Appendix 1
Brief Summary of INTERVENTION MANUAL for SAFE (Systemic Autism-related Family Enabling): an early intervention for families of children with autism and Asperger Syndrome

Professor Rudi Dallos and Doctor Rebecca McKenzie

The intervention manual has been developed from the following bases of evidence and clinical experience:

1. Systemic Family Therapy.

Systemic Family Therapy focuses on promoting positive changes in the relationships within families rather than the isolated behavior of individuals in the family (Dallos & Draper, 2015). Difficulties are seen to be maintained or exacerbated by family dynamics including the organization of families, communicational patterns and repetitive interaction sequences of problems. The combination of these is encapsulated in the idea of the formation of ineffective attempted solutions to their problems which rather than offering solutions can aggravate their difficulties. Systemic Family Therapy embodies a range of approaches and techniques for helping families to explore and re-organise their understanding, relationship patterns, emotional connections and problem-solving abilities. It typically involves a therapist and a supervision team working with one family at a time with close monitoring, based on feedback during and at the end of each session exploring the relevance, helpfulness and applicability of the therapy. Where supervision teams are not available a more flexible model - 'in room consultation' can be employed whereby two therapists support each other in a structure where one therapist takes on the role of monitoring the family's reactions as therapy proceeds and periodically offering feedback as a form of live supervision. A development of this orientation is Multi-Family Therapy whereby groups of families work together to assist and facilitate change, effectively acting as consultants for each other. The SAFE program employs both these versions of Systemic Family Therapy with three single family sessions to explore in detail each family's needs; and two group sessions using concepts and techniques from Multi-Family Therapy

2. Multi-Family Therapy

Multi-Family Therapy is a recognised treatment approach which aims to provide a more empowering, flexible and intensive form of family intervention than single family therapy (Asen & Scholz, 2009). In Multi-Family Therapy similar techniques are employed to those used in single family therapy, but families are encouraged to work together, to be proactive in solving their own problems Multi-Family Therapy aims to help families rediscover their own resources by emphasising how families can take an active role in tackling dilemmas and assisting each other. At the same time families are encouraged to use the group setting to explore how problems have affected family life in consultation with other families and to share their solutions and competencies. This involves a power shift from the therapist-client relationship and encourages an empowering peer-support environment. The sharing of experiences and the dynamics of the group are important components of the treatment.

3. Family Models

The SAFE program incorporates a range of concepts and techniques by integrating systemic and attachment based family models, in particular The Circle of Security Intervention (Powell et al., 2014) and Attachment Narrative Therapy developed by co-applicant Professor Rudi Dallos (Dallos, 2006). In the SAFE intervention, these systemic and attachment models have been combined with an emphasis on visual materials and active, play-based approaches designed to be appropriate for families of children with autism.

For peer review only - http://bmjopen.bmj.com/site/about/guidelines.xhtml

4. Autism friendly

SAFE has been developed in collaboration with children with autism and their families to be autism friendly. Given the evidence in the literature for a visual processing style, communication difficulties and restricted interests among people with autism, the advice of families is in line with current research. SAFE incorporates a multi-sensory approach using visual materials which are integrated into therapeutic techniques and play-based activities. SAFE also acknowledges and works with the children's talents and areas of special interest. It also recognizes that parents are often extremely well-informed and prefer an approach which recognizes their competencies and helps them to feel empowered rather than de-skilled by therapy.

SAFE sessions and examples of activities:

SAFE involves 5 x 3-hour sessions outlined in a detailed manual. All sessions are facilitated by two therapists trained in the SAFE intervention model. Around 6 families receive the SAFE intervention as a cohort, supported by two therapists. Sessions 1 and 5 are based on Multi-Family Therapy and all parents from the 6 families attend. Sessions 2, 3 and 4 are for single families and all family members attend including siblings and/or grandparents if the family wish. So the 6 families meet each other at the beginning and the end but the in-between sessions are just for their own family.

SAFE is supported by a detailed manual setting out a structure for each session and also a toolkit of activities which can be used flexibly according to family need and the age and symptoms of the child.

Examples of activities include the following:

Tracking a circularity

Families explore the events of positive and problematic cycles of events, such as 'meltdowns' by breaking these down to explore in detail what happened, including actions, understandings, expectations and how people felt. E.g. what happened first, then what happened next? What did you do? What did Mum do? This can allow families to unpick problematic and successful cycles and initiate positive change.

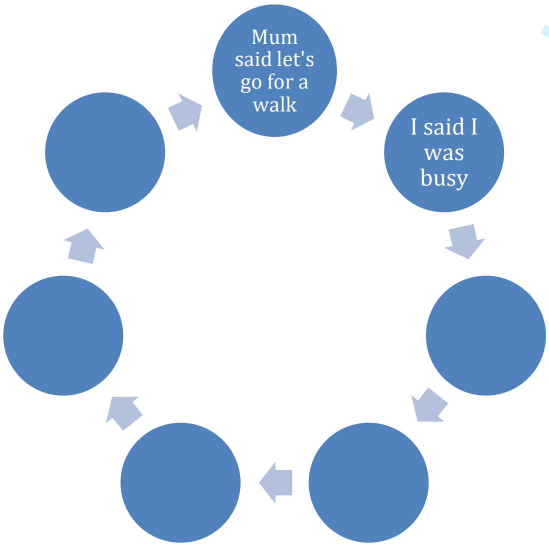

For peer review only - http://bmjopen.bmj.com/site/about/guidelines.xhtml

A family genogram

The families are asked to create a genogram (family tree) this is used to explore relationships, perceptions of autism, family narratives and avenues of support and conflict. E.g. who is most supportive to you? Who also has autistic traits? Who is most similar to you? What are the stories you have heard from grandparents etc.

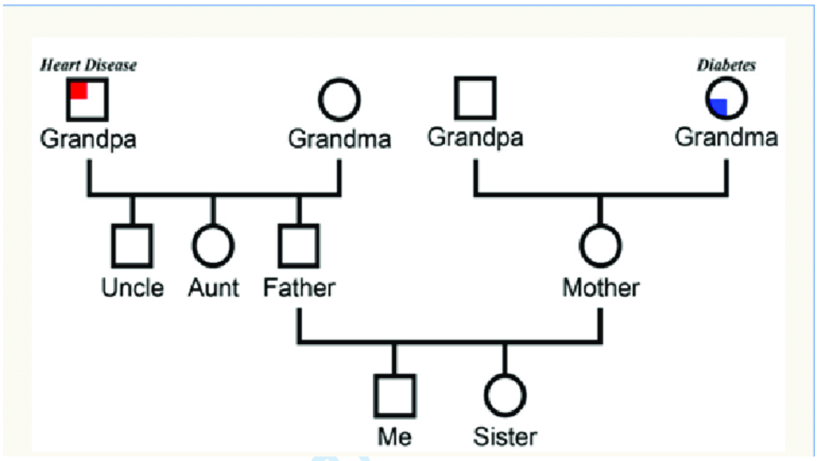

The Circle of Security (COS)

The COS intervention and the graphic designed around it are designed to help parents increase awareness of their children's needs and whether their own responses meet those needs. With increased awareness parents can expand their moment-to-moment parenting choices where needed. The model, therefore, encourages the potential to break the stranglehold of problematic patterns. Discussion can include: the parents as a secure base and haven, procedural memory, parents' attachment needs, implicit responding, children as miscuing.

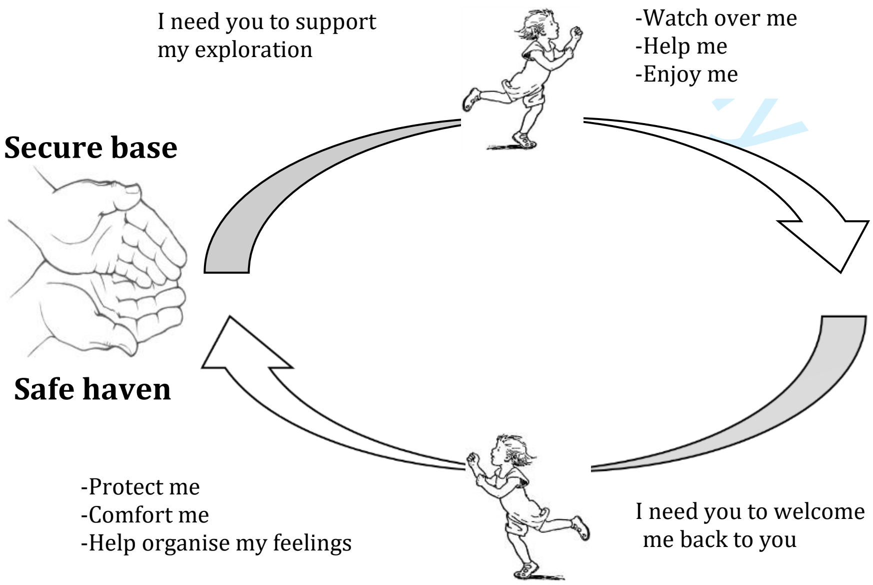

For peer review only - http://bmjopen.bmj.com/site/about/guidelines.xhtml

Externalising and Self-Autism Mapping (SAM)

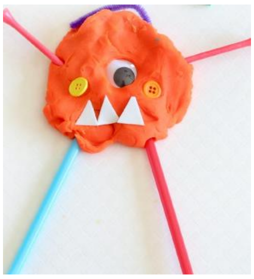

Children may be invited to make 'autism' from PlayDoh. The parents discuss the child's responses and elaborate with their own views of what they regard as typical as opposed to autistic and how the influence of the autism may alter in different contexts and reduce or expand as the child develops.

This is developed by inviting the child to engage in mapping the autism (SAM) which is initiated by introducing the child to two figures representing 'Just me' and "Me and autism' Redrawing these figures can involve the child engaged in a discussion of the two figures in terms of what is, and is not affected by autism. At a later date a dotted circle is added and the children can identify aspects of themselves about which they are unsure.

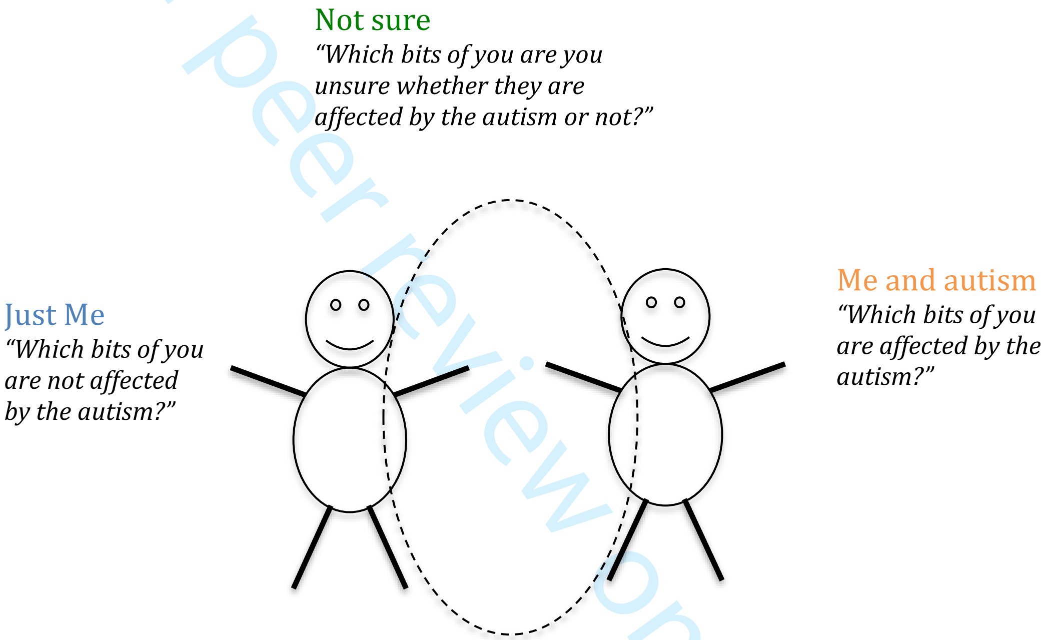

Area of Special Interest (ASI)

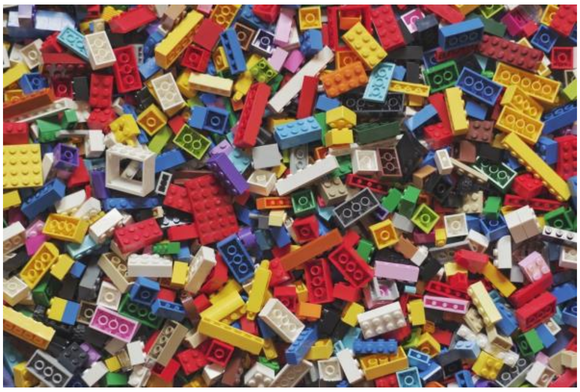

The Child with autism and the siblings are invited to bring an object or present their interest of special interest. This can take and form that the child is comfortable with. The ASI is used to raise the child's confidence, model communication and explore relationships, perceptions and difficulties. E.g. if mum was a Lego character who would she be? Who would you be? How do the characters manage difficulties? etc.

For peer review only - http://bmjopen.bmj.com/site/about/guidelines.xhtml

Button Sculpt

Families use buttons to map and discuss relationships (how close they are to each other) before and after the diagnosis and how they predict it might be in the future. This helps to the families understand the relationships, feelings and perceptions at key times and also reflect on how these may change and develop.

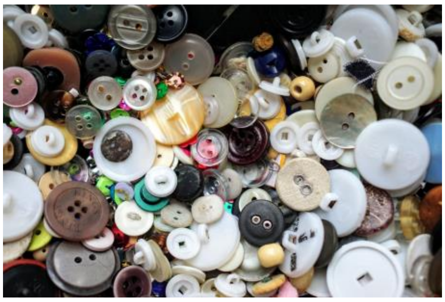

Additional activities

Role play
Often used with tracking to try out new approaches or rehearse successful interactions or strategies.

Reflective conversations
Therapists have a conversation about their thoughts on the family issues and dynamics. The family are invited to listen and then to discuss between themselves and the therapists what was of interest and relevant, what they agreed or disagreed with etc. This is an opportunity to build a sense of openness, support the therapeutic relationships, validate the family, praise successes and also to offer specific suggestions.

Drawing
Family members draw aspects of their life e.g. a typical day or a day at school. Drawing can also be used as part of SAM if the child wishes to draw autism or themselves as part of the activity.

Video feedback activities
Families watch video training materials of other families and use tracking techniques to analyse what is happening, make connections with their own experience and suggest ways forward. This is used in sessions 1 and 5 with the parents. They are asked to watch videos of a child and mum dealing with a meltdown and in the final session a young person with autism describing how he manages emotional and sensory difficulties. These activities are intended to help reflection in a non-challenging way and empower parents to act as consultants to others in similar positions.

For peer review only

Key references

Asen, E., and Scholz, M. (2009) *Multi-Family Therapy: Concepts and Techniques*, London: Routledge

Dallos, R. (2006) *Attachment Narrative Therapy: Integrating Attachment , Systemic and Narrative Therapies.* Maidenhead: Open Univ. Press/McGraw Hill

Dallos, R. and Draper, R. (2015 4th Edn) *An Introduction to Family Therapy*. Maidenhead: Open Univ. Press/McGraw Hill

Powell, B., Cooper, G., Hoffman, K. and Marvin, B. (2014). *The Circle of Security Intervention: Enhancing Attachment in Early – Child Relationships.* London: Guilford Press

CONSORT 2010 checklist of information to include when reporting a randomised trial*

Section/Topic	Item No	Checklist item	Reported on page No
**Title and abstract**			
	1a	Identification as a randomised trial in the title	1
	1b	Structured summary of trial design, methods, results, and conclusions (for specific guidance see CONSORT for abstracts)	1
**Introduction**			
Background and objectives	2a	Scientific background and explanation of rationale	3
	2b	Specific objectives or hypotheses	3
**Methods**			
Trial design	3a	Description of trial design (such as parallel, factorial) including allocation ratio	4
	3b	Important changes to methods after trial commencement (such as eligibility criteria), with reasons	4
Participants	4a	Eligibility criteria for participants	4
	4b	Settings and locations where the data were collected	4
Interventions	5	The interventions for each group with sufficient details to allow replication, including how and when they were actually administered	4,5
Outcomes	6a	Completely defined pre-specified primary and secondary outcome measures, including how and when they were assessed	5,6
	6b	Any changes to trial outcomes after the trial commenced, with reasons	None
Sample size	7a	How sample size was determined	N/A
	7b	When applicable, explanation of any interim analyses and stopping guidelines	N/A
Randomisation:			
Sequence generation	8a	Method used to generate the random allocation sequence	N/A
	8b	Type of randomisation; details of any restriction (such as blocking and block size)	5
Allocation concealment mechanism	9	Mechanism used to implement the random allocation sequence (such as sequentially numbered containers), describing any steps taken to conceal the sequence until interventions were assigned	N/A
Implementation	10	Who generated the random allocation sequence, who enrolled participants, and who assigned participants to interventions	5
Blinding	11a	If done, who was blinded after assignment to interventions (for example, participants, care providers, those	5

For peer review only

For peer review only - http://bmjopen.bmj.com/site/about/guidelines.xhtml

		assessing outcomes) and how	
	11b	If relevant, description of the similarity of interventions	N/A
Statistical methods	12a	Statistical methods used to compare groups for primary and secondary outcomes	6
	12b	Methods for additional analyses, such as subgroup analyses and adjusted analyses	6
**Results**			
Participant flow (a diagram is strongly recommended)	13a	For each group, the numbers of participants who were randomly assigned, received intended treatment, and were analysed for the primary outcome	See consort diagram
	13b	For each group, losses and exclusions after randomisation, together with reasons	As above
Recruitment	14a	Dates defining the periods of recruitment and follow-up	As above
	14b	Why the trial ended or was stopped	N/A
Baseline data	15	A table showing baseline demographic and clinical characteristics for each group	6
Numbers analysed	16	For each group, number of participants (denominator) included in each analysis and whether the analysis was by original assigned groups	6,7,8
Outcomes and estimation	17a	For each primary and secondary outcome, results for each group, and the estimated effect size and its precision (such as 95% confidence interval)	N/A
	17b	For binary outcomes, presentation of both absolute and relative effect sizes is recommended	N/A
Ancillary analyses	18	Results of any other analyses performed, including subgroup analyses and adjusted analyses, distinguishing pre-specified from exploratory	
Harms	19	All important harms or unintended effects in each group (for specific guidance see CONSORT for harms)	7
**Discussion**			
Limitations	20	Trial limitations, addressing sources of potential bias, imprecision, and, if relevant, multiplicity of analyses	2 and 11
Generalisability	21	Generalisability (external validity, applicability) of the trial findings	10/11
Interpretation	22	Interpretation consistent with results, balancing benefits and harms, and considering other relevant evidence	10/11
**Other information**			
Registration	23	Registration number and name of trial registry	2
Protocol	24	Where the full trial protocol can be accessed, if available	3
Funding	25	Sources of funding and other support (such as supply of drugs), role of funders	

*We strongly recommend reading this statement in conjunction with the CONSORT 2010 Explanation and Elaboration for important clarifications on all the items. If relevant, we also recommend reading CONSORT extensions for cluster randomised trials, non-inferiority and equivalence trials, non-pharmacological treatments, herbal interventions, and pragmatic trials. Additional extensions are forthcoming: for those and for up to date references relevant to this checklist, see www.consort-statement.org.

For peer review only - http://bmjopen.bmj.com/site/about/guidelines.xhtml
